# Identification of tumor mutation burden-related hub genes and the underlying mechanism in melanoma

**DOI:** 10.7150/jca.53697

**Published:** 2021-03-01

**Authors:** Chuan Zhang, Dan Dang, Chenlu Liu, Yuqian Wang, Xianling Cong

**Affiliations:** 1 Department of Dermatology, China-Japan Union Hospital of Jilin University, Changchun 130033, People's Republic of China.; 2 Department of Pediatric Surgery, the First Hospital of Jilin University, Changchun 130021, People's Republic of China.; 3 Department of Neonatology, the First Hospital of Jilin University, Changchun 130021, People's Republic of China.; 4 Department of Tissue Bank, China-Japan Union Hospital of Jilin University, Changchun, 130033, People's Republic of China.; 5 Scientific Research Center, China-Japan Union Hospital of Jilin University, Changchun 130033, People's Republic of China.

**Keywords:** tumor mutation burden, WGCNA, tumor-infiltrating immune cells, biomarker, ceRNA

## Abstract

**Background:** Tumor mutation burden (TMB) has emerged as an important predictive factor for drug resistance in cancers; however, the specific mechanism underlying TMB function in melanoma remains elusive.

**Methods:** Data on somatic mutations, RNA sequencing (RNA-seq), miRNA sequencing (miRNA-seq), and clinical characteristics for 472 melanoma patients were extracted from the TCGA cohort. RNA-seq data of melanoma cell lines were obtained from the Cancer Cell Line Encyclopedia, and sensitivity of cell lines to therapeutic agents is available in the Cancer Therapeutics Response Portal. TMB was calculated based on somatic mutation data. Differentially expressed gene analysis, weighted gene co-expression network analysis, protein-protein interaction networks, Minimal Common Oncology Data Elements, and survival analysis were leveraged to determine TMB-related hub genes. Competing endogenous RNA (ceRNA) networks were constructed to explore the molecular mechanisms underlying hub gene function. The influence of key genes on drug sensitivity was analyzed to investigate their clinical significance.

**Results:** Elevated TMB levels were significantly correlated with improved survival outcomes. In addition, six tumor-infiltrating immune cells, including naive B cells, regulatory T cells, memory resting CD4 T cells, memory B cells, activated mast cells, and resting NK cells, were significantly overexpressed in the low-TMB group relative to the high-TMB group. Furthermore, we identified *FLNC, NEXN,* and *TNNT3* as TMB-related hub genes, and constructed their ceRNA networks, including five miRNAs (has-miR-590-3p, has-miR-374b-5p, has-miR-3127-5p, has-miR-1913, and has-miR-1291) and 31 lncRNAs (*FAM66C, MIAT, NR2F2AS1*, etc.). Finally, we observed that TMB-related genes were associated with distinct therapeutic responses to AKT/mTOR pathway inhibitors.

**Conclusions:** We identified three TMB-associated key genes, established their ceRNA networks, and investigated their influence on therapeutic responses, which could provide insights into future precision medicine.

## Introduction

Cutaneous melanoma is the deadliest type of skin cancer 1, and its morbidity remains increasing annually, especially in the Caucasian population 2, 3. Immunotherapies, including CTLA-4 and PD1/PDL1 inhibitors, are the preferred treatments for advanced melanoma 4-6. However, approximately half of melanoma patients treated with immunotherapies will develop primary or acquired resistance 7-10, which poses a major challenge for improving therapies. There are no highly accurate predictive biomarkers of therapy resistance, and there are limited effective treatment options available once resistance develops 11. Therefore, it is imperative to identify novel predictive biomarkers for melanoma treatment to guide clinical decision-making.

Tumor mutation burden (TMB) is defined as the total number of variants in the whole length of exons, and it is regarded as a novel predictor of response to immunotherapy. TMB levels are correlated with advantageous immune-related prognosis in patients with breast cancer 12 and lung cancer 13, and TMB combined with CpGs can predict objective responses to PD-1/PD-L1 inhibition blockade 14. Nevertheless, the detailed mechanism of TMB function in melanoma remains elusive.

With advances in gene sequencing technology, a wealth of gene databases, such as the Cancer Genome Atlas (TCGA) 15 and Gene Expression Omnibus (GEO), are emerging. Meanwhile, a series of bioinformatics tools, including weighted gene co-expression network analysis (WGCNA) 16, CYBERSORT 17, gene set enrichment analysis (GSEA) 18, and least absolute shrinkage and selection operator (LASSO), are emerging that can help to process such big data. The combination of these databases and bioinformatics means has produced numerous scientific achievements.

In this study, we aimed to explore the mechanism underlying TMB function in melanoma. To this end, we analyzed RNA-seq data and corresponding phenotypic data using bioinformatics methods to identify TMB-related hub genes, investigate their competing endogenous RNA networks, and investigate the effect of TMB on the immune microenvironment and drug sensitivity. This study provides new insights into the molecular mechanism of melanoma development and provides a reference for clinical decision-making in melanoma treatment.

## Materials and Methods

### Data Acquisition and Genome-Wide Mutation Profiling

Data on somatic mutations, RNA-seq, miRNA-seq, and clinical characteristics for 472 patients with melanoma were obtained from the TCGA cohort using the GDC tool.

R package “maftools” 19, which could count frequencies of various variant classifications and distributions of different types of variant genes, was utilized to analyze somatic mutation data.

### Comprehensive investigation into effect of TMB on clinical features

TMB was calculated using the following formula: TMB = (total amount of truncating mutation × 1.5 + total amount of non-truncating mutation × 1.0)/ whole length of exons 20. The truncating mutations include nonsense, frame-shift deletion, frame-shift insertion, and splice-site mutations, while non-truncating mutations refer to missense, in-frame deletion, in-frame insertion, and nonstop mutations. Truncating mutations were allocated a greater weight because of their stronger deleterious influence on gene expression than that of non-truncating mutations.

We performed differentially expressed genes (DEG) analysis based on RNA-seq data using R package “edgeR” 21, which implements a series of statistical methods including empirical Bayes estimation, exact tests, generalized linear models, and quasi-likelihood tests. DEG analysis was performed between the low- and high-TMB groups, with a cutoff value of the median TMB value. Selection criteria for DEGs were as follows: |logFC| > 1 and *P* < 0.05.

Functional annotation of DEGs was conducted using DAVID bioinformatics resources 22, which provides a comprehensive set of functional annotation tools for researchers to comprehend the biological meaning behind specific gene sets.

Relationships between TMB and clinical features, including clinical stage, pathological type, and survival, were analyzed to determine the clinical significance of TMB.

### Effect of TMB on tumor immune microenvironment

Since TMB is associated with immunity, we sought to investigate the relationship between TMB and tumor-infiltrating immune cells (TIICs), which could be estimated using CYBERSORT. CIBERSORT is an in silico algorithm that enables precise estimation of immune cell fractions using RNA-seq profiles for bulk samples 17. The accuracy of CIBERSORT has been demonstrated by immunohistochemistry and flow cytometry. The operating parameters used in the present study were as follows: B-mode, disable quantile normalization, and permutation for significance analysis 100. We filtered out the samples with *P*> 0.05 to increase the accuracy of the estimated results.

### Exploration of the TMB-related Hub Genes and underlying molecular mechanism

Weighted Gene Co-expression Network Analysis (WGCNA), a systematic algorithm 16 for weighted correlation network analysis, serves for identifying modules of highly correlated genes and relating modules to TMB. RNA-seq data for 56499 genes from 472 melanoma patients were normalized into TPM format. After removing 35312 genes with extremely low expression values, 21187 genes were used to construct a weighted correlation network and identify modules. TMB values for patients were used as external clinical traits and related to modules.

The protein-protein interaction (PPI) networks were constructed based on genes in the most TMB-relevant module, using the STRING database (version 11.0) of known and predicted protein-protein interactions 23, which now covers 24584628 proteins from 5090 organisms. The users only need to submit a list of gene symbols and species, and the website provides interaction relationships among submitted proteins. These interactions include direct (physical) and indirect (functional) associations.

The PPI network was then exported to a local folder in the TSV file format, and further analyzed using Minimal Common Oncology Data Elements (MCODE) 24, a Cytoscape plug-in (version 3.8.0) 25, which identified clusters (highly interconnected regions) in a network. The setting parameters were as follows: Degree Cutoff 2, Node Score Cutoff 0.2, K-Core 2, and Max Depth 100.

To further clarify the molecular mechanism underlying TMB-related hub genes, we constructed a competing endogenous RNA (ceRNA) network. The ceRNA theory suggests that any RNA transcript that harbors MREs can sequester miRNAs from other targets sharing the same MREs, thereby regulating their expression 26, 27. Importantly, the ability of two transcripts to cross-regulate each other can be bioinformatically predicted based on the MREs that they have in common 26. First, we retrieved five miRNA-related databases (miRWalk, miRanda, miRDB, RNA22, and Targetscan) to obtain hub gene-associated miRNAs. The miRNAs that were presented in at least four databases were isolated and further assessed using the log-rank test. Then, pairing relationships between miRNAs and lncRNAs were obtained by retrieving the lncBase v2.0 database and subsequently performing the log-rank test. At this point, the ceRNA network was completed.

### Correlation between Hub Genes and Drug Sensitivity

Since TMB was reported as a novel predictor of response to immunotherapy, we investigated associations between TMB-related hub genes and sensitivity of melanoma cells to therapeutic drugs. Data on melanoma cell lines were obtained from two large-scale cancer profiling studies: the Cancer Cell Line Encyclopedia (CCLE) 28-29, which profiles gene expression in cancer cells, and the Cancer Therapeutics Response Portal (CTRP) 30, which characterizes the response of cancer cell lines to a collection of drugs. We categorized melanoma cell lines into low- and high-expression groups based on median RNA expression values, and compared sensitivity to therapeutic drugs in the high- versus low- group. The IC50 of each drug was used as a measure of drug response.

### Statistics

All statistical analyses were completed using R software (Version 4.0.1). The normal distribution of continuous variables was evaluated using the Shapiro-Wilk test, and the homogeneity of variance was evaluated using Bartlett's test. According to the data homogeneity of variance and normal distribution, either the independent sample t test or Wilcoxon signed rank test was chosen. The log-rank test was used to evaluate survival significance. Spearman's correlation coefficient was used to assess the correlation between two continuous variables. The correlation intensity was divided into five grades based on the absolute value of the partial correlation coefficient: 0.00-0.19 corresponded to very weak, 0.20-0.39 to weak, 0.40-0.59 to moderate, 0.60-0.79 to strong, and 0.80-1.0 to very strong 31. *P* < 0.05 was considered statistically significant.

## Results

### Genome-wide mutation profiling in melanoma

**Figure 1:**
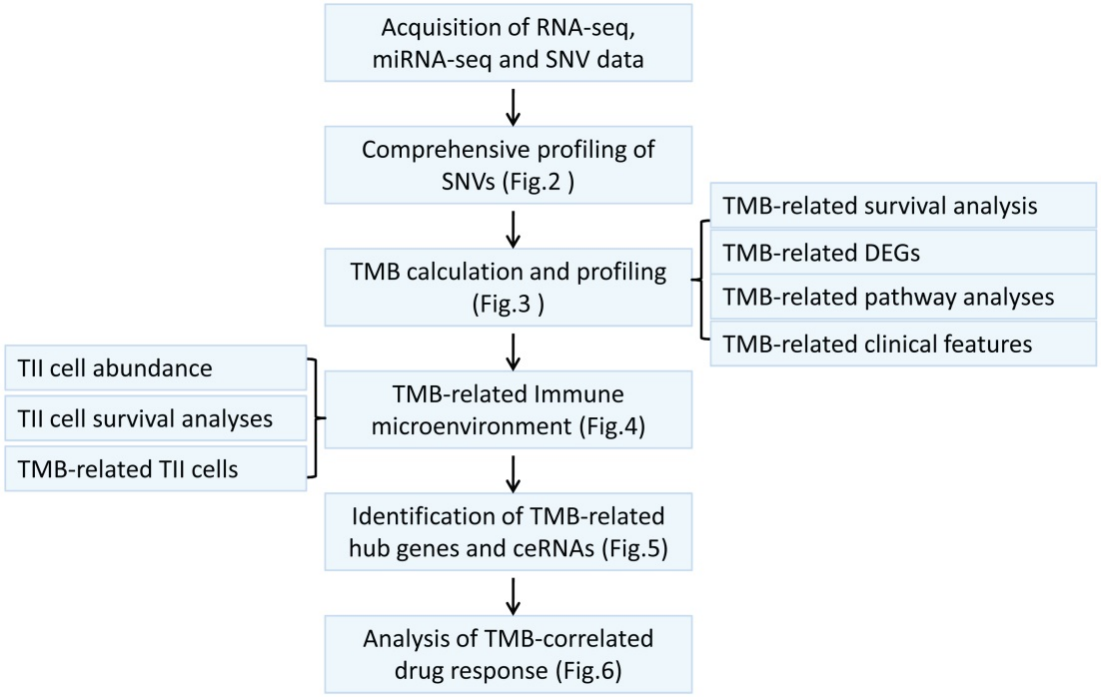
The workflow of this study.

**Figure 2:**
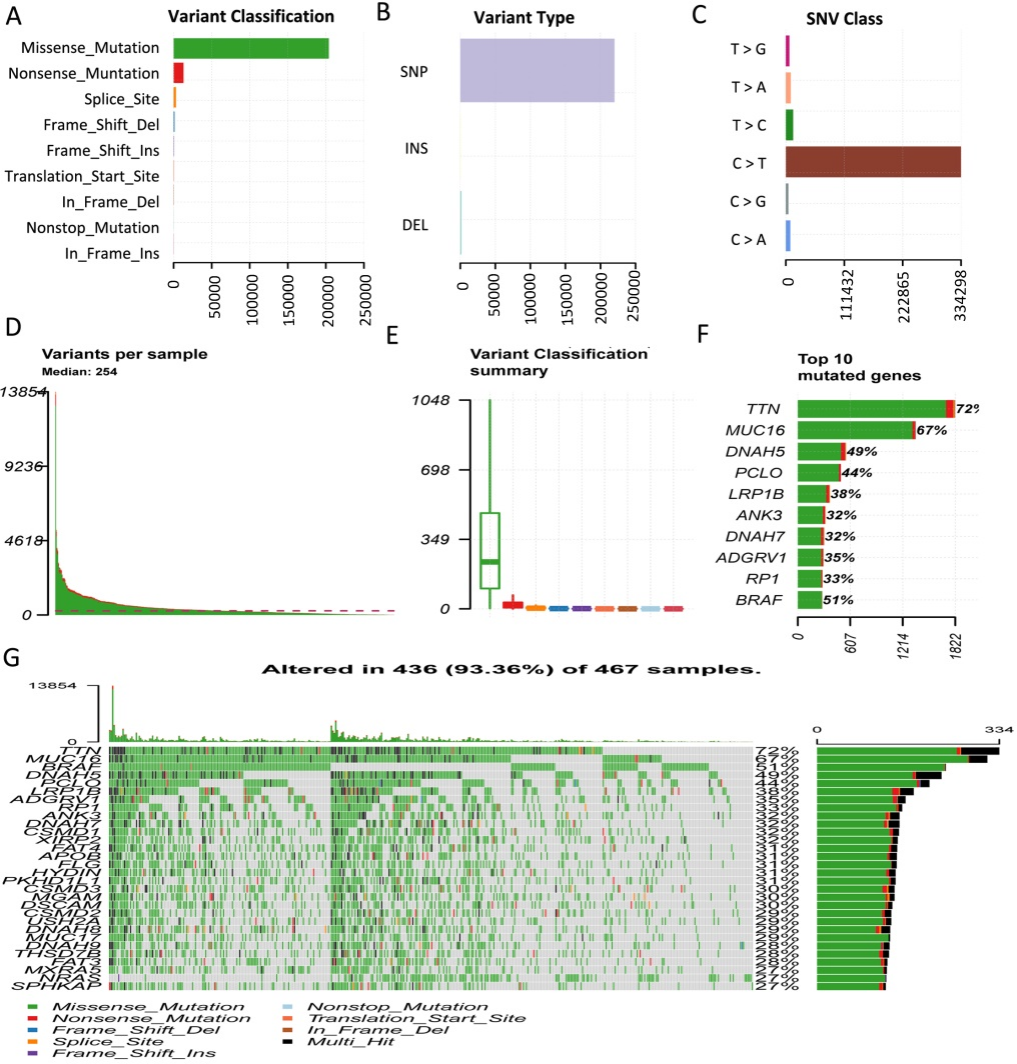
Comprehensive profiling of somatic mutation data. (A) Variant classification of melanoma is displayed, and missense mutation is the most frequent mutation. (B) SNP constitutes the vast majority in variant types. (C) C > T is the most common type of SNV class. (D) The amount of mutated bases in each patient is shown, with a median value of 254. (E) Variant classification summary. (F) The top 10 mutated genes are shown. (G) Waterfall plot shows distribution of all variant classifications in all patients. Different colors with specific annotations at the bottom represent different variant classifications. SNV: single nucleotide variation.

Considering somatic mutations as the molecular basis of TMB, we first characterized genome-wide variations by analyzing somatic mutation data of melanoma. An overview of the analytical strategy is shown in Figure 1. We observed that missense mutations, nonsense mutations, and splice sites were the top three frequent variation types (Figure 2A), and single-nucleotide polymorphism (SNP) constituted the vast majority of variant types (Figure 2B). C > T was the most common type of single nucleotide variation (SNV) class (Figure 2C). Moreover, we displayed the number of mutated bases in each of the patients, with a median value of 254 (Figure 2D). The top 10 mutated genes in melanoma were *TTN* (72%), *MUC16* (67%), *DNAH5* (49%), *PCLO* (44%), *LRP1B* (38%), *ANK3* (32%), *DNAH7* (32%), *ADGRV1* (35%), *RP1* (33%), and *BRAF* (51%) (Figure 2F). The waterfall plot showed distribution of all variant classifications in all patients (Figure 2G).

### Comprehensive investigation of the role of TMB in clinical traits

**Figure 3:**
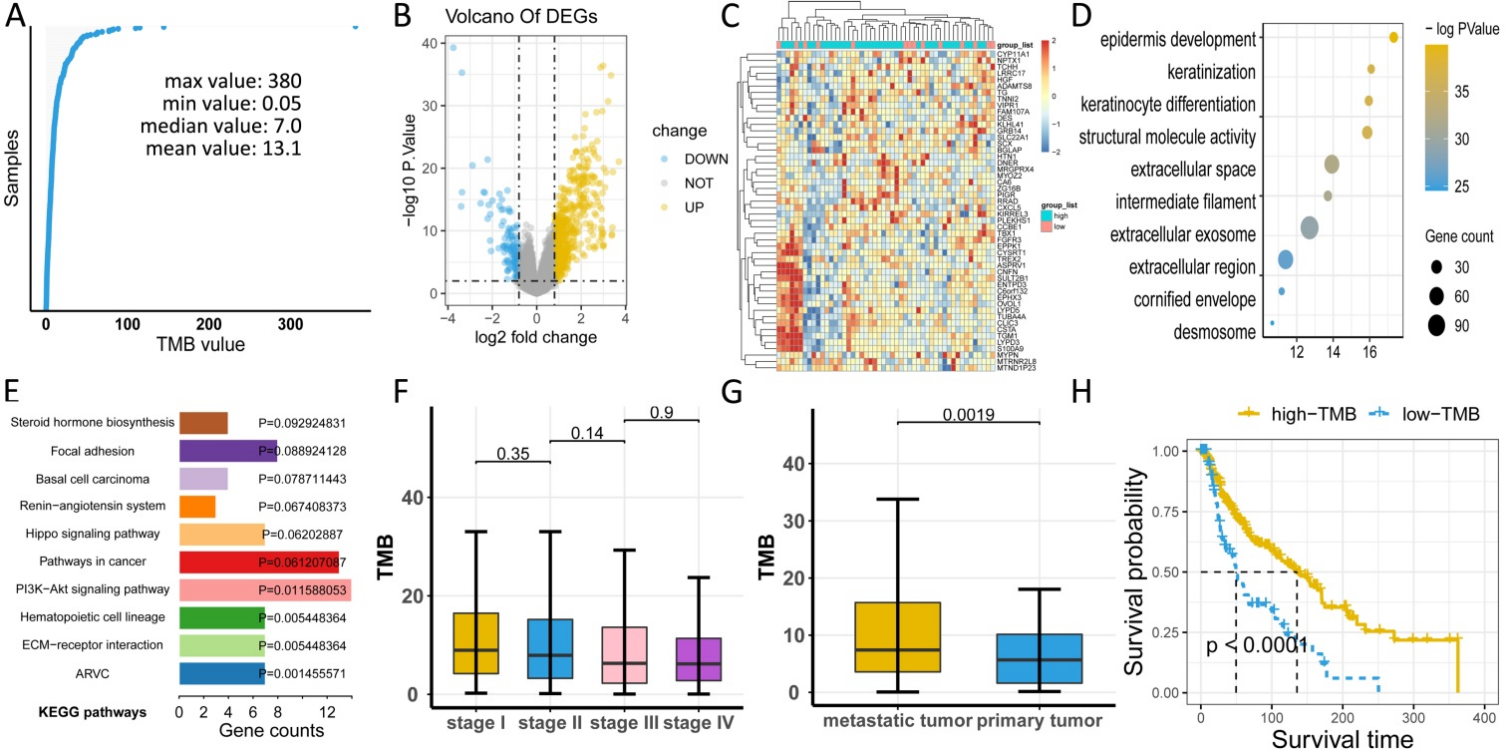
Comprehensive profiling of TMB in melanoma. (A) Distribution of TMB values in patients. (B) High-TMB group shows 443 DEGs compared to low-TMB group. (C) Heatmap of DEGs displays distinct expression levels between high- and low-TMB groups. (D) Gene oncology (GO) analyses of DEGs. (E) KEGG pathways of DEGs. (F) TMB has no relationship with tumor stage (*P*> 0.05). (G) TMB value is significantly higher in metastatic melanoma than in primary melanoma (*P* < 0.05). (H) High-TMB is significantly associated with advantageous survival outcomes. TMB: tumor mutation burden; DEGs: differentially expressed genes.

To uncover the clinical implications of mutations in melanoma, we first calculated the TMB value in all patients, obtaining a median value of 7.0 and a mean value of 13.1 (Figure 3A, Table S1). Through different expression analyses, we observed 443 DEGs (370 upregulated and 73 downregulated) in the high-TMB group versus the low-TMB group (Figure 3B, Table S2). The top 10 upregulated genes were *FGFR3, TCHH, CNFN, TGM1, SULT2B1, ENTPD3, SLC22A1, TBX1, VIPR1,*and* TREX2*, while the top ten downregulated genes were* ZG16B, TG, ADAMTS8, PIGR, KLHL41, DES, CA6, MRGPRX4, RRAD,* and *SCX* (*P*< 0.05, |logFC| >1, Table S1). The heatmap of DEGs showed distinct expression levels between high- and low-TMB groups (Figure 3C). Functional enrichment analysis demonstrated that TMB-related pathways include epidermal development, keratinization, and keratinocyte differentiation (Figure 3D, Table S3). Meanwhile, the top three KEGG pathways were steroid hormone biosynthesis, focal adhesion, and basal cell carcinoma (Figure 3E, Table S1). TMB was significantly correlated with sample pathological types (*P* < 0.05, Figure 3G), but not with the tumor stage (*P* > 0.05, Figure 3F). Consistent with previous findings, high-TMB was significantly associated with improved survival (*P* < 0.0001, Figure 3H).

### Investigation of the TMB-related TIICs

**Figure 4:**
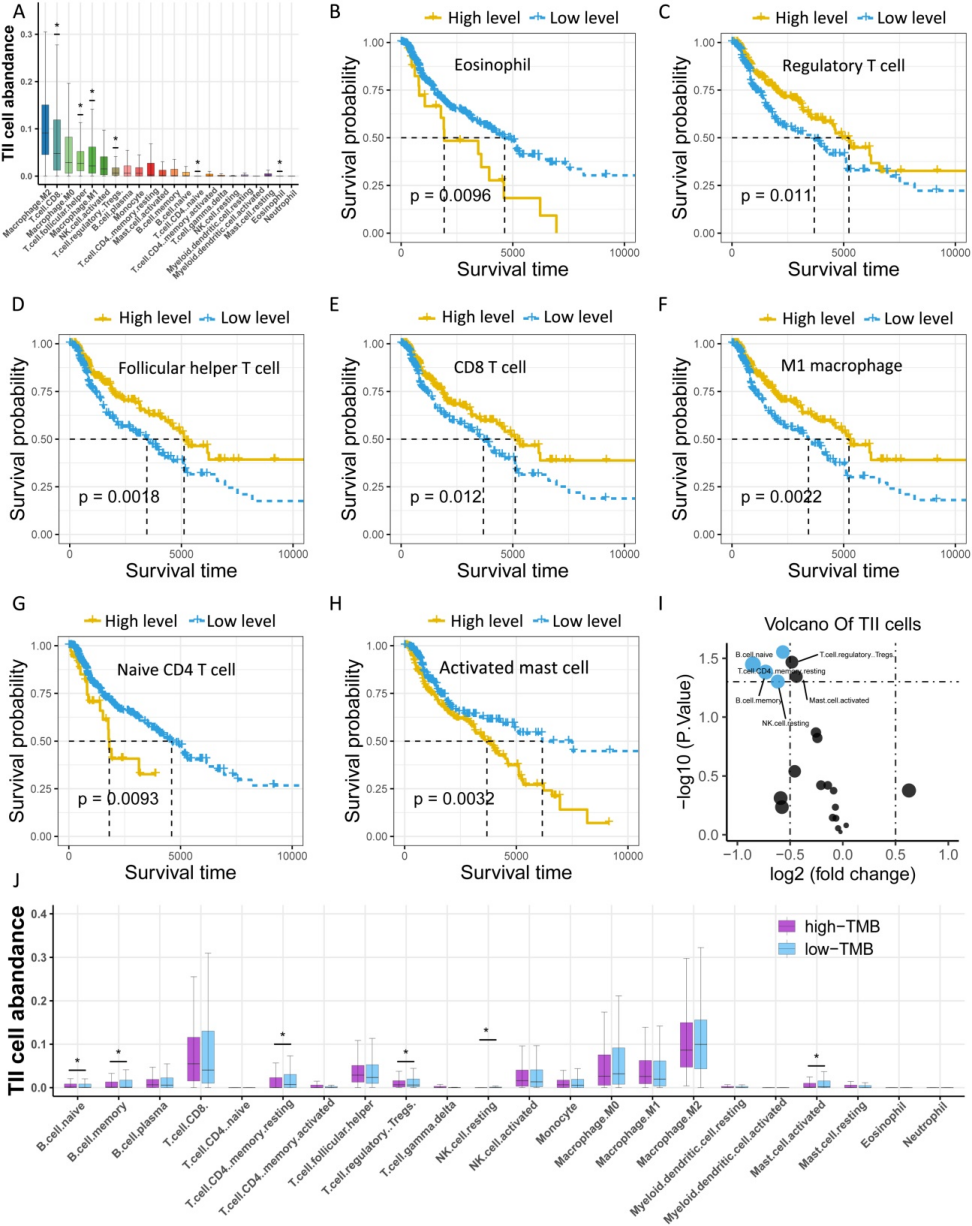
Investigation of the TMB-related TIICs. (A) Distribution of 22 types of TllCs in melanoma. Asterisk (*) represents the specific type of immune cells which is significantly correlated with survival. (B-H) Kaplan-Meier curves of relevant TIICs. (I) Volcano plot of differentially expressed TIICs between low- and high-TMB groups. (J) Box plots demonstrate differential expression levels of TIICs between low- and high-TMB groups. Asterisk (*) indicated a statistical significance. TIICs: tumor infiltrating immune cells.

Considering that TMB is associated with immunity, we investigated the abundance of tumor-infiltrating immune cells (TIICs) in melanoma using CYBERSORT, and found that M2 macrophages, CD8 T cells, and M0 macrophages were the top three TIICs with the highest abundance (Figure 4A, Table S4). Moreover, seven types of TIICs were significantly related to survival, including eosinophils, regulatory T (Treg) cells, T follicular helper cells, CD8 T cells, M1 macrophages, naive CD4 T cells, and activated mast cells (Figure 4B-H). Furthermore, we sought to clarify the association of TMB with TIICs by categorizing samples into low- and high-TMB groups and performed differential expression analysis of TIICs. The findings revealed six types of immune cells that were significantly upregulated in the low-TMB group compared to the high-TMB group, including naive B cells, memory resting CD4 T cells, Treg cells, memory B cells, activated mast cells, and resting NK cells (Figure 4I-J).

### Identification and Validation of TMB-related Hub Genes and ceRNAs

**Figure 5:**
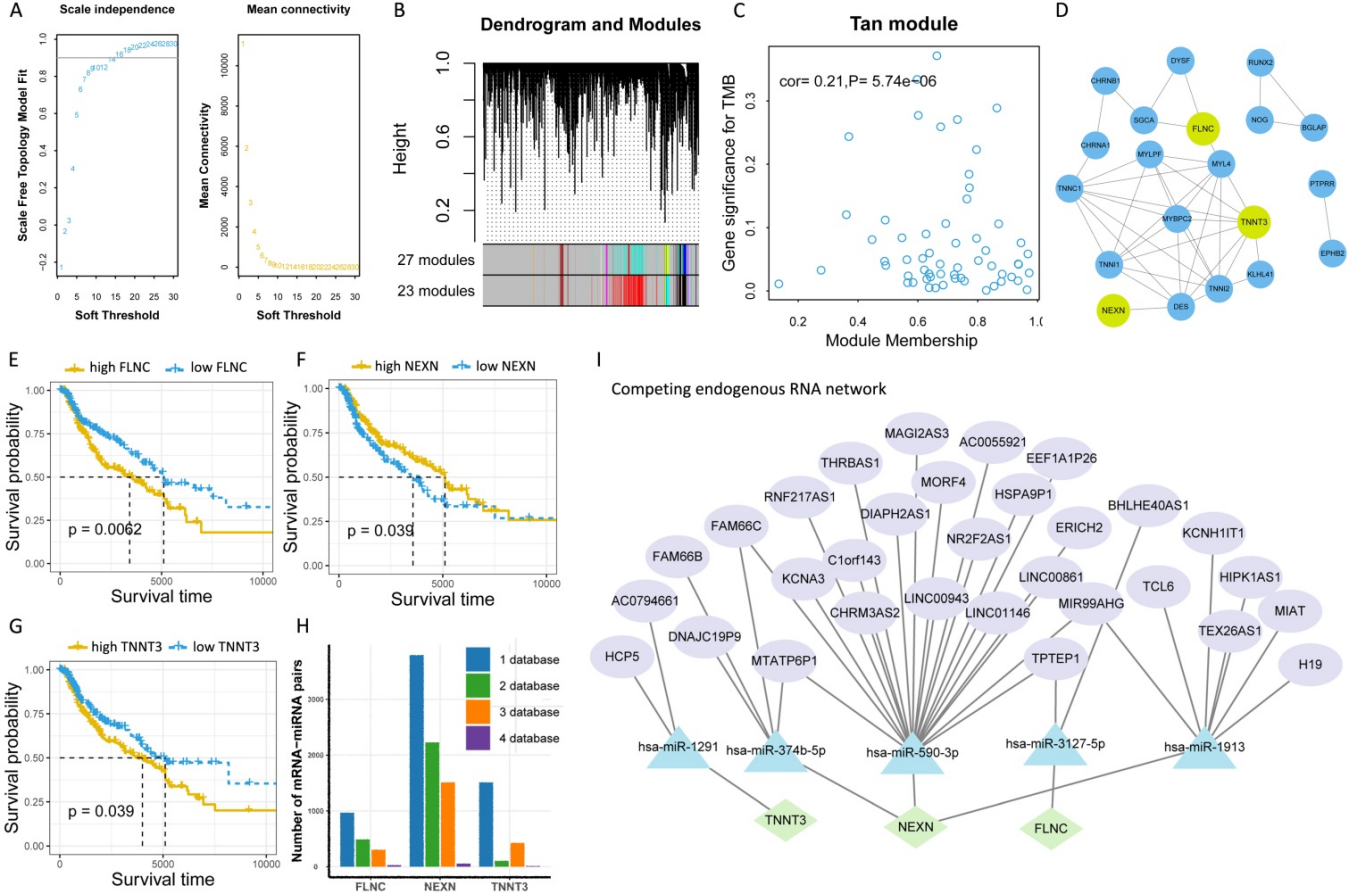
Identification of TMB-related hub genes and ceRNAs. (A) Determination of soft threshold used for WGCNA. (B) All genes are clustered into 23 modules, and different colors mean different modules. (C) Scatter plot displays the genes in Tan Module (59 genes). (D) Fifty-nine genes in Tan Module are further analyzed using PPI network. (E-G) Three genes *(FLNC, TNNT3, NEXN)* are eventually identified as hub TMB-related genes using WGCNA, PPI and log-rank test.(H) Five online databases (miRWalk, miRanda, miRDB, RNA22, and Targetscan) are utilized to search for hub gene-related miRNAs, and mRNA-miRNA pairs which occur in at least four databases are selected as candidate mRNA-miRNA pairs. (I) Hub genes-associated ceRNAs are displayed.

To further explore the potential molecular mechanism underlying TMB, we next identified TMB-related hub genes by performing WGCNA based on RNA-seq data and TMB data. We first acquired 21187 genes by filtering out 35312 extremely low-expressed genes from 56499 genes, and then constructed a weighted correlation network. To build a scale-free network, we determined β = 14 as the soft-threshold power (Figure 5A). A hierarchical clustering tree was established using dynamic hybrid cutting. Each leaf on the tree represented a single gene, and genes with close correlation formed a branch of the tree, representing a gene module (Figure 5B). Among the 23 modules, the Tan module was the most related to TMB (cor =0.21, *P* = 5.74e-10, Figure 5C). Fifty-nine genes in the Tan module were further analyzed using the PPI network and MCODE (Figure 5D), and the genes in the biggest cluster were assessed using the log-rank test. Eventually, three genes (*FLNC, NEXN,* and *TNNT3*) were identified as TMB-related hub genes (Figure 5E-G).

To further clarify the molecular mechanism underlying TMB-related hub genes, we constructed a competing endogenous RNA (ceRNA) network. Five miRNA-mRNA pairs (*NEXN* and has-miR-590-3p, *NEXN* and has-miR-374b-5p, *FLNC* and has-miR-3127-5p, *FLNC* and has-miR-1913, *TNNT3* and has-miR-1291), and 36 pairs of miRNA-lncRNA were determined to be competing endogenous RNAs. At this point, the ceRNA network was completed, consisting of 3 mRNAs, 5 miRNAs, and 31 lncRNAs (Figure 5I, Table S5).

### TMB-related gene expression was reflective of drug responses

**Figure 6:**
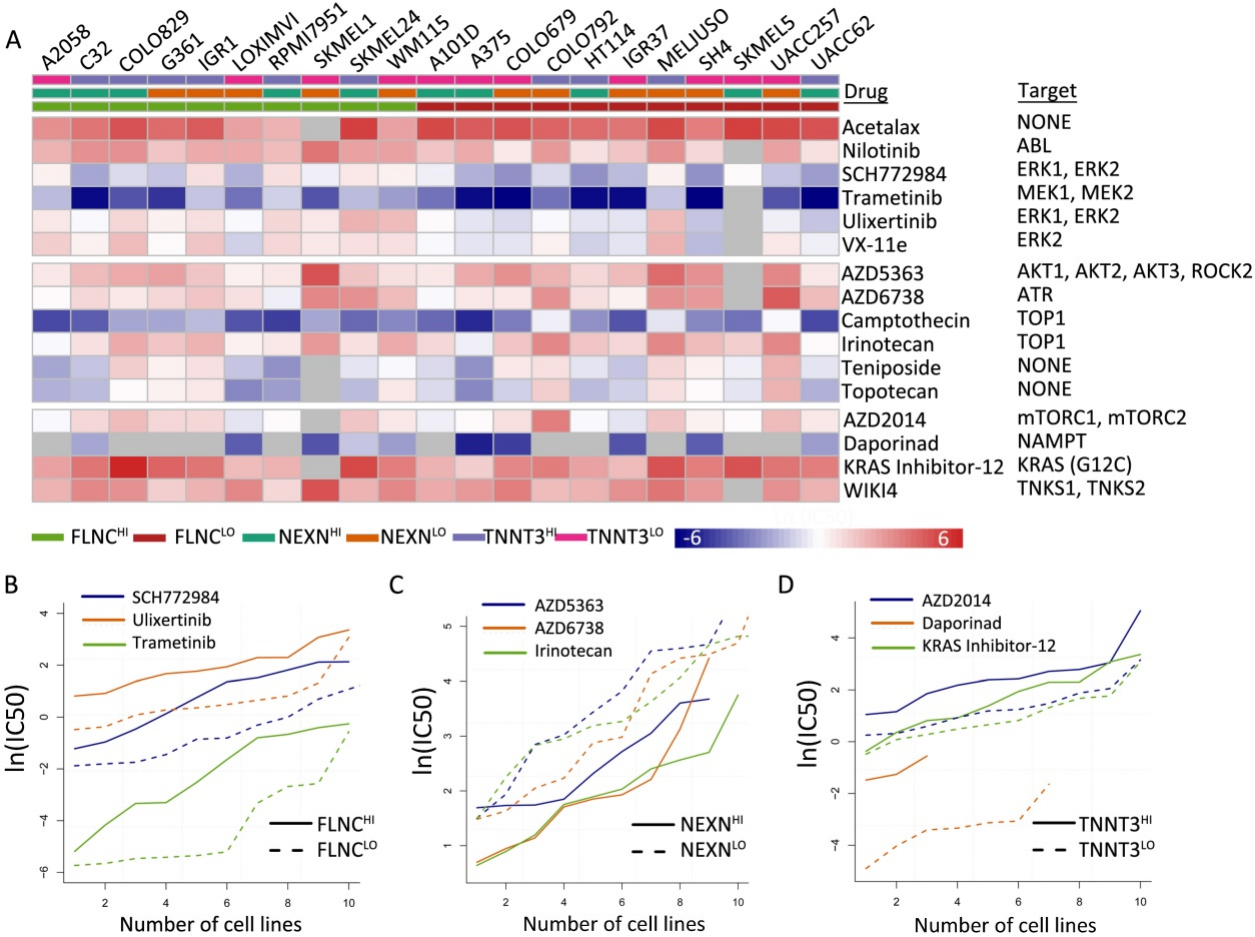
TMB-related gene expression is reflective of drug responses. (A) Heatmap shows differential drug sensitivity of the indicated drugs in different groups. (B) *TNNT3^HI^* cells are more sensitive to AKT/mTOR pathway inhibitors. (C) *NEXN^HI^* cells are more sensitive to AKT/mTOR pathway inhibitors. (D) *FLCN^LO^*melanoma cell lines are more sensitive to MEK/ERK pathway inhibitors.

We identified 16 types of therapeutic agents that were significantly correlated with hub gene expression levels (Figure 6A, Table S6). Consistent with previous results, which demonstrated that elevated *NEXN* expression level was reflective of improved survival outcomes while reduced *FLNC* or *TNNT3* levels were associated with beneficial survival (Figure 6B-D), we observed that *NEXN^HI^* cells were more sensitive to AKT/mTOR pathway inhibitors (Figure 6C), whereas *FLCN^LO^* or *TNNT3^LO^*melanoma cell lines were more sensitive to MEK/ERK pathway inhibitors or AKT/mTOR pathway inhibitors, respectively (Figure 6B, D). Interestingly, *NEXN^HI^* cells were more sensitive to AKT/mTOR pathway inhibitors, whereas *TNNT3^LO^* cells were significantly correlated with AKT/mTOR pathway inhibitors.

## Discussion

Resistance to immunotherapy in melanoma poses an urgent challenge, while TMB has emerged as a prospective predictor of whether cancer patients respond to immunotherapy or not 32. The present study demonstrates that melanoma is indeed of high heterogenicity, and high levels of TMB are significantly related to improved survival in melanoma. In addition, TMB-related TIICs, genes, and ceRNAs were determined to unravel the potential mechanism underlying the role of TMB in melanoma. Moreover, we observed that TMB-related genes were associated with distinct therapeutic responses. Our findings reveal that TMB plays an important role in melanoma and provides insights into future precision medicine.

Melanoma is a carcinoma with high mutational burden and heterogenicity. *TTN, MUC16, DNAH5, PCLO, LRP1B, ANK3, DNAH7, ADGRV1, RP1*, and *BRAF* were the top 10 mutated genes in melanoma. Considering its high heterogenicity, it makes sense that molecular targeted therapies are not broadly effective in melanoma 33-34. Previous studies have observed that missense mutations are the most frequent type of bladder urothelial carcinoma 35. Consistent with this, we also have revealed that missense mutations are most frequently observed, and C>T occurs more frequently than other single-nucleotide variants in melanoma.

We also determined *FLNC, NEXN,* and* TNNT3* as TMB-related hub genes and exposed the underlying molecular mechanism. *FLNC* has been reported to correlate with cardiomyopathy 36, 37, but its role in melanoma is unknown. *NEXN* can control actin polymerization in smooth muscle 38, and its implication on melanoma has not been reported. In addition, *TNNT3* is a risk factor for breast cancer 39, while its role in melanoma is unclear. Our findings add strength to the evidence that *FLNC, NEXN,* and* TNNT3* could be potential therapeutic targets. The role of has-miR-590-3p in cancer remains controversial, with its beneficial effect in glioblastoma multiforme 40 and an unfavorable impact on epithelial ovarian cancer 41. Herein, we observe that has-miR-590-3p could promote the progression of melanoma by inhibiting *NEXN*. has-miR-374b-5p can suppress bladder 42, ovarian 43, and pancreatic cancers 44, while it can promote gastric cancer cell invasion and metastasis 45. We also showed thathas-miR-374b-5p is a potentially cancerous molecule because of blocking the expression of *NEXN*. Currently, there is no literature addressing the role of miR-374b-5p in melanoma. Similarly, has-miR-3127-5p, has-miR-1913, and has-miR-1291 have critical effects on various cancers, but their implications in melanoma have not been reported so far. Collectively, these hub genes and ceRNAs could be utilized as potential therapeutic targets in melanoma immunotherapies.

Moreover, we reveal that the levels of naive B cells, Treg cells, memory resting CD4 T cells, memory B cells, activated mast cells, and resting NK cells are significantly higher in the low-TMB group than in the high-TMB group. Intriguingly, we observed that high levels of Treg cells were linked to improved survival in melanoma, which seems to contradict the previous finding that Treg cells could mitigate antitumor immune responses 46. Treg cells have been associated with both favorable and poor prognoses in various human cancers 47-49. Moreover, Treg subpopulations are heterogeneous, and different Treg subpopulations may have opposing effects on tumor progression 50-53. Meanwhile, Treg cells in the melanoma tumor microenvironment are driven by CD8^+^ T cells 54, which indicates that patients with elevated Treg cells could bear augmented CD8^+^ T cells simultaneously, thus having an improved survival. Memory B cells are considered to be major targets for effective immunotherapy in relapsing multiple sclerosis 55, buttheir role in immunotherapy for melanoma is elusive. Presently, the roles of naive B cells, memory resting CD4 T cells, and memory B cells in the development of melanoma are not well understood. Our findings would provide guidance for designing future studies to further clarify this issue.

This study has important implications for both the prognosis and treatment of melanoma. The findings herein reveal that melanoma is a tumor of high heterogenicity, which supports the idea that immunotherapies should be utilized as the first-line therapy for advanced melanoma. Second, we observed that naive B cells, Treg cells, memory resting CD4 T cells, memory B cells, activated mast cells, and resting NK cells were upregulated in low-TMB populations, which could shed light into the immune mechanism underlying the development of melanoma. Furthermore, three TMB-associated genes and their ceRNAs were identified, which could be utilized as candidate predictive biomarkers and therapeutic targets. In addition, we revealed that TMB-related genes were associated with distinct therapeutic responses to AKT/mTOR pathway inhibitors.

This study also has limitations and requires further research. First, the ceRNA networks were constructed based on a comprehensive analysis of data from online databases, such as the relationship between *TNNT3* and has-miR-1291, which requires further *in vitro* and *in vivo* experiments to verify these findings. Secondly, although TMB-related genes reflect distinct therapeutic responses to AKT/mTOR pathway inhibitors, the specific molecular mechanism is elusive, and remains to be explored in later experiments.

In conclusion, to comprehensively investigate the role of TMB in melanoma, by analyzing data on RNA-seq, somatic mutations, and clinical characteristics for skin melanoma using a series of bioinformatics approaches, we reveal that TMB has a substantial effect on melanoma. TMB as well as TMB-related hub genes and their corresponding ceRNAs, could serve as candidate predictive biomarkers and therapeutic targets. Our study provides the opportunity to develop more effective immunotherapy strategies aimed at treating melanoma.

## Supplementary Material

Supplementary tables.
