# Supplementary material for: Identification of tumor mutation burden-related hub genes and the underlying mechanism in melanoma
Source: J Cancer. 2021 Mar 1;12(8):2440–9. doi: 10.7150/jca.53697 (PMC7974884; doi:10.7150/jca.53697)
Supplement: Supplementary file 1 — Supplementary tables. [file jcav12p2440s1.zip › Table-S3-DAVID.pdf]

Category	Term
GOTERM_BP_DIRECT	GO:0008544~epidermis development
GOTERM_BP_DIRECT	GO:0031424~keratinization
GOTERM_BP_DIRECT	GO:0030216~keratinocyte differentiation
GOTERM_MF_DIRECT	GO:0005198~structural molecule activity
GOTERM_CC_DIRECT	GO:0005615~extracellular space
GOTERM_CC_DIRECT	GO:0005882~intermediate filament
GOTERM_CC_DIRECT	GO:0070062~extracellular exosome
GOTERM_CC_DIRECT	GO:0005576~extracellular region
GOTERM_CC_DIRECT	GO:0001533~cornified envelope
GOTERM_CC_DIRECT	GO:0030057~desmosome
GOTERM_BP_DIRECT	GO:0018149~peptide cross-linking
GOTERM_BP_DIRECT	GO:0061436~establishment of skin barrier
GOTERM_MF_DIRECT	GO:0004252~serine-type endopeptidase activity
GOTERM_CC_DIRECT	GO:0005578~proteinaceous extracellular matrix
GOTERM_BP_DIRECT	GO:0006508~proteolysis
GOTERM_MF_DIRECT	GO:0005200~structural constituent of cytoskeleton
GOTERM_CC_DIRECT	GO:0005886~plasma membrane
GOTERM_BP_DIRECT	GO:0016337~single organismal cell-cell adhesion
GOTERM_BP_DIRECT	GO:0030855~epithelial cell differentiation
GOTERM_CC_DIRECT	GO:0005887~integral component of plasma membrane
GOTERM_MF_DIRECT	GO:0004867~serine-type endopeptidase inhibitor activity
GOTERM_CC_DIRECT	GO:0045095~keratin filament
GOTERM_MF_DIRECT	GO:0086083~cell adhesive protein binding involved in bundle of His cell-P
GOTERM_BP_DIRECT	GO:0086073~bundle of His cell-Purkinje myocyte adhesion involved in cell
GOTERM_MF_DIRECT	GO:0005509~calcium ion binding
GOTERM_BP_DIRECT	GO:0006958~complement activation, classical pathway
GOTERM_BP_DIRECT	GO:0010951~negative regulation of endopeptidase activity
GOTERM_BP_DIRECT	GO:0045109~intermediate filament organization
GOTERM_BP_DIRECT	GO:0006955~immune response
GOTERM_CC_DIRECT	GO:0009986~cell surface
GOTERM_BP_DIRECT	GO:0043588~skin development
GOTERM_CC_DIRECT	GO:0005916~fascia adherens
GOTERM_MF_DIRECT	GO:0019215~intermediate filament binding
GOTERM_BP_DIRECT	GO:0030336~negative regulation of cell migration
GOTERM_BP_DIRECT	GO:0008284~positive regulation of cell proliferation
GOTERM_BP_DIRECT	GO:0045110~intermediate filament bundle assembly
GOTERM_BP_DIRECT	GO:0010482~regulation of epidermal cell division
KEGG_PATHWAY	hsa05412:Arrhythmogenic right ventricular cardiomyopathy (ARVC)
GOTERM_BP_DIRECT	GO:0098911~regulation of ventricular cardiac muscle cell action potential
GOTERM_CC_DIRECT	GO:0031012~extracellular matrix
GOTERM_BP_DIRECT	GO:0031581~hemidesmosome assembly
GOTERM_MF_DIRECT	GO:0008236~serine-type peptidase activity
GOTERM_CC_DIRECT	GO:0014704~intercalated disc
GOTERM_BP_DIRECT	GO:0031069~hair follicle morphogenesis
GOTERM_CC_DIRECT	GO:0031225~anchored component of membrane
GOTERM_BP_DIRECT	GO:0002159~desmosome assembly
GOTERM_BP_DIRECT	GO:0006956~complement activation
GOTERM_BP_DIRECT	GO:0001501~skeletal system development
GOTERM_BP_DIRECT	GO:0002009~morphogenesis of an epithelium
GOTERM_BP_DIRECT	GO:0045165~cell fate commitment
GOTERM_CC_DIRECT	GO:0016324~apical plasma membrane
GOTERM_BP_DIRECT	GO:0048485~sympathetic nervous system development
GOTERM_BP_DIRECT	GO:0045104~intermediate filament cytoskeleton organization
GOTERM_BP_DIRECT	GO:0048701~embryonic cranial skeleton morphogenesis
GOTERM_CC_DIRECT	GO:0016328~lateral plasma membrane
KEGG_PATHWAY	hsa04512:ECM-receptor interaction
KEGG_PATHWAY	hsa04640:Hematopoietic cell lineage

GOTERM_MF_DIRECT	GO:0030674~protein binding, bridging
GOTERM_MF_DIRECT	GO:0003823~antigen binding
GOTERM_BP_DIRECT	GO:0030282~bone mineralization
GOTERM_BP_DIRECT	GO:0042475~odontogenesis of dentin-containing tooth
GOTERM_MF_DIRECT	GO:0008201~heparin binding
GOTERM_BP_DIRECT	GO:0006898~receptor-mediated endocytosis
GOTERM_BP_DIRECT	GO:0051216~cartilage development
GOTERM_CC_DIRECT	GO:0005922~connexon complex
GOTERM_BP_DIRECT	GO:0045606~positive regulation of epidermal cell differentiation
GOTERM_BP_DIRECT	GO:0032496~response to lipopolysaccharide
GOTERM_BP_DIRECT	GO:0007267~cell-cell signaling
GOTERM_BP_DIRECT	GO:0009967~positive regulation of signal transduction
GOTERM_CC_DIRECT	GO:0005911~cell-cell junction
GOTERM_BP_DIRECT	GO:0001895~retina homeostasis
GOTERM_BP_DIRECT	GO:0001822~kidney development
GOTERM_CC_DIRECT	GO:0030056~hemidesmosome
KEGG_PATHWAY	hsa04151:PI3K-Akt signaling pathway
GOTERM_BP_DIRECT	GO:0007155~cell adhesion
GOTERM_BP_DIRECT	GO:0016264~gap junction assembly
GOTERM_BP_DIRECT	GO:0043627~response to estrogen
GOTERM_MF_DIRECT	GO:0003810~protein-glutamine gamma-glutamyltransferase activity
GOTERM_CC_DIRECT	GO:0019897~extrinsic component of plasma membrane
GOTERM_BP_DIRECT	GO:0010838~positive regulation of keratinocyte proliferation
GOTERM_BP_DIRECT	GO:2000146~negative regulation of cell motility
GOTERM_MF_DIRECT	GO:0045294~alpha-catenin binding
GOTERM_BP_DIRECT	GO:0030335~positive regulation of cell migration
GOTERM_BP_DIRECT	GO:0030148~sphingolipid biosynthetic process
GOTERM_MF_DIRECT	GO:0050786~RAGE receptor binding
GOTERM_BP_DIRECT	GO:0061045~negative regulation of wound healing
GOTERM_BP_DIRECT	GO:0055078~sodium ion homeostasis
GOTERM_CC_DIRECT	GO:0045111~intermediate filament cytoskeleton
GOTERM_CC_DIRECT	GO:0009925~basal plasma membrane
GOTERM_BP_DIRECT	GO:0007010~cytoskeleton organization
GOTERM_CC_DIRECT	GO:0005604~basement membrane
GOTERM_BP_DIRECT	GO:0043616~keratinocyte proliferation
GOTERM_CC_DIRECT	GO:0031410~cytoplasmic vesicle
GOTERM_MF_DIRECT	GO:0005215~transporter activity
GOTERM_BP_DIRECT	GO:0030198~extracellular matrix organization
GOTERM_MF_DIRECT	GO:0001077~transcriptional activator activity, RNA polymerase II core pror
GOTERM_BP_DIRECT	GO:0030073~insulin secretion
GOTERM_MF_DIRECT	GO:0005070~SH3/SH2 adaptor activity
GOTERM_BP_DIRECT	GO:0042060~wound healing
GOTERM_BP_DIRECT	GO:0043410~positive regulation of MAPK cascade
GOTERM_BP_DIRECT	GO:0050680~negative regulation of epithelial cell proliferation
GOTERM_MF_DIRECT	GO:0005243~gap junction channel activity
GOTERM_MF_DIRECT	GO:0004869~cysteine-type endopeptidase inhibitor activity
GOTERM_BP_DIRECT	GO:0010839~negative regulation of keratinocyte proliferation
GOTERM_BP_DIRECT	GO:0010001~glial cell differentiation
GOTERM_BP_DIRECT	GO:0051897~positive regulation of protein kinase B signaling
GOTERM_CC_DIRECT	GO:0016323~basolateral plasma membrane
GOTERM_BP_DIRECT	GO:0048286~lung alveolus development
GOTERM_BP_DIRECT	GO:0086091~regulation of heart rate by cardiac conduction
GOTERM_MF_DIRECT	GO:0004872~receptor activity
GOTERM_BP_DIRECT	GO:0034220~ion transmembrane transport
GOTERM_BP_DIRECT	GO:0050776~regulation of immune response
GOTERM_BP_DIRECT	GO:0042633~hair cycle
GOTERM_BP_DIRECT	GO:0045669~positive regulation of osteoblast differentiation
GOTERM_MF_DIRECT	GO:0050839~cell adhesion molecule binding

GOTERM_BP_DIRECT	GO:0010043~response to zinc ion
GOTERM_BP_DIRECT	GO:0010623~programmed cell death involved in cell development
GOTERM_BP_DIRECT	GO:0001806~type IV hypersensitivity
GOTERM_BP_DIRECT	GO:0071624~positive regulation of granulocyte chemotaxis
GOTERM_BP_DIRECT	GO:0060529~squamous basal epithelial stem cell differentiation involved ir
GOTERM_BP_DIRECT	GO:0060512~prostate gland morphogenesis
GOTERM_BP_DIRECT	GO:0070488~neutrophil aggregation
GOTERM_BP_DIRECT	GO:0007160~cell-matrix adhesion
GOTERM_BP_DIRECT	GO:0007586~digestion
GOTERM_BP_DIRECT	GO:0009887~organ morphogenesis
GOTERM_CC_DIRECT	GO:0030425~dendrite
GOTERM_MF_DIRECT	GO:0004866~endopeptidase inhibitor activity
GOTERM_BP_DIRECT	GO:0010466~negative regulation of peptidase activity
GOTERM_BP_DIRECT	GO:0032570~response to progesterone
GOTERM_MF_DIRECT	GO:0005201~extracellular matrix structural constituent
GOTERM_BP_DIRECT	GO:0051384~response to glucocorticoid
GOTERM_MF_DIRECT	GO:0008083~growth factor activity
GOTERM_BP_DIRECT	GO:0001580~detection of chemical stimulus involved in sensory perceptior
GOTERM_BP_DIRECT	GO:0006366~transcription from RNA polymerase II promoter
GOTERM_BP_DIRECT	GO:0001709~cell fate determination
GOTERM_BP_DIRECT	GO:0033280~response to vitamin D
GOTERM_CC_DIRECT	GO:0009898~cytoplasmic side of plasma membrane
GOTERM_BP_DIRECT	GO:0038096~Fc-gamma receptor signaling pathway involved in phagocytc
KEGG_PATHWAY	hsa05200:Pathways in cancer
GOTERM_MF_DIRECT	GO:0004999~vasoactive intestinal polypeptide receptor activity
GOTERM_MF_DIRECT	GO:0047718~indanol dehydrogenase activity
GOTERM_MF_DIRECT	GO:0002020~protease binding
KEGG_PATHWAY	hsa04390:Hippo signaling pathway
GOTERM_BP_DIRECT	GO:0060348~bone development
GOTERM_CC_DIRECT	GO:0043005~neuron projection
GOTERM_BP_DIRECT	GO:0060197~cloacal septation
GOTERM_BP_DIRECT	GO:0032602~chemokine production
GOTERM_BP_DIRECT	GO:1902732~positive regulation of chondrocyte proliferation
GOTERM_BP_DIRECT	GO:0060675~ureteric bud morphogenesis
GOTERM_BP_DIRECT	GO:0060672~epithelial cell morphogenesis involved in placental branching
GOTERM_BP_DIRECT	GO:0043163~cell envelope organization
GOTERM_BP_DIRECT	GO:0098869~cellular oxidant detoxification
GOTERM_BP_DIRECT	GO:0071300~cellular response to retinoic acid
GOTERM_BP_DIRECT	GO:0014823~response to activity
KEGG_PATHWAY	hsa04614:Renin-angiotensin system
GOTERM_BP_DIRECT	GO:0042493~response to drug
GOTERM_MF_DIRECT	GO:0020037~heme binding
GOTERM_BP_DIRECT	GO:0006915~apoptotic process
GOTERM_MF_DIRECT	GO:0004601~peroxidase activity
KEGG_PATHWAY	hsa05217:Basal cell carcinoma
GOTERM_BP_DIRECT	GO:0006810~transport
GOTERM_BP_DIRECT	GO:0001764~neuron migration
GOTERM_BP_DIRECT	GO:0045471~response to ethanol
GOTERM_MF_DIRECT	GO:0097110~scaffold protein binding
GOTERM_CC_DIRECT	GO:0097209~epidermal lamellar body
GOTERM_MF_DIRECT	GO:0035662~Toll-like receptor 4 binding
GOTERM_BP_DIRECT	GO:0090023~positive regulation of neutrophil chemotaxis
GOTERM_MF_DIRECT	GO:0017134~fibroblast growth factor binding
GOTERM_BP_DIRECT	GO:0030324~lung development
GOTERM_BP_DIRECT	GO:0030509~BMP signaling pathway
GOTERM_MF_DIRECT	GO:0008009~chemokine activity
GOTERM_BP_DIRECT	GO:0046545~development of primary female sexual characteristics
GOTERM_BP_DIRECT	GO:0002070~epithelial cell maturation

GOTERM_BP_DIRECT	GO:0043587~tongue morphogenesis
GOTERM_BP_DIRECT	GO:0002793~positive regulation of peptide secretion
GOTERM_BP_DIRECT	GO:0048608~reproductive structure development
GOTERM_BP_DIRECT	GO:0010760~negative regulation of macrophage chemotaxis
GOTERM_BP_DIRECT	GO:0032119~sequestering of zinc ion
KEGG_PATHWAY	hsa04510:Focal adhesion
GOTERM_MF_DIRECT	GO:0004222~metalloendopeptidase activity
GOTERM_CC_DIRECT	GO:0005856~cytoskeleton
KEGG_PATHWAY	hsa00140:Steroid hormone biosynthesis
GOTERM_BP_DIRECT	GO:0050728~negative regulation of inflammatory response
GOTERM_BP_DIRECT	GO:0009954~proximal/distal pattern formation
GOTERM_BP_DIRECT	GO:0001503~ossification
GOTERM_BP_DIRECT	GO:0010923~negative regulation of phosphatase activity

Count	%	PValue	Genes	List Total	Pop Hits	Pop Total	Fold Enrich	Bonferroni
2.30E+01	5.386417	4.78E-18	S100A7, C	366	85	16792	12.41453	7.88E-15
1.80E+01	4.215457	8.26E-17	TCHH, LCE	366	48	16792	17.20492	1.83E-13
2.10E+01	4.918033	1.12E-16	S100A7, TI	366	76	16792	12.67731	1.83E-13
3.30E+01	7.728337	1.36E-16	KRT6C, KR	356	247	16881	6.33527	5.46E-14
7.50E+01	17.5644	1.19E-14	LYPD3, MA	383	1347	18224	2.649346	2.78E-12
2.20E+01	5.152225	1.96E-14	KRT6C, KR	383	113	18224	9.2638	4.60E-12
1.16E+02	27.16628	1.98E-13	S100A8, S1	383	2811	18224	1.963551	4.62E-11
7.80E+01	18.26698	4.07E-12	MTRNR2L8	383	1610	18224	2.305227	9.53E-10
1.40E+01	3.278689	6.70E-12	LCE2A, SPI	383	46	18224	14.48155	1.57E-09
1.10E+01	2.576112	2.20E-11	JUP, EVPL,	383	24	18224	21.80853	5.15E-09
1.40E+01	3.278689	3.36E-11	LCE2A, SPI	366	50	16792	12.84634	5.53E-08
1.00E+01	2.34192	4.02E-11	KDF1, CLD	366	18	16792	25.48877	6.63E-08
2.30E+01	5.386417	2.36E-08	IGLV1-40,	356	255	16881	4.276966	1.16E-05
2.30E+01	5.386417	5.63E-08	ELN, CHI3I	383	268	18224	4.083551	1.32E-05
3.20E+01	7.494145	1.71E-07	IGLV1-40,	366	500	16792	2.936306	2.83E-04
1.30E+01	3.044496	3.44E-06	KRT6A, KR	356	110	16881	5.604009	0.001691
1.25E+02	29.274	5.16E-06	F2RL2, IGL	383	4121	18224	1.443286	0.001206
1.20E+01	2.810304	1.22E-05	JUP, PKP1,	366	101	16792	5.451063	0.019887
1.00E+01	2.34192	1.98E-05	FGFR2, RH	366	70	16792	6.554254	0.032175
5.40E+01	12.64637	2.59E-05	F2RL2, CLD	383	1415	18224	1.81586	0.006051
1.10E+01	2.576112	3.70E-05	SERPINB5,	356	97	16881	5.37736	0.018034
1.10E+01	2.576112	4.71E-05	KRT6C, KR	383	100	18224	5.234047	0.010972
4.00E+00	0.936768	1.76E-04	JUP, PKP2,	356	6	16881	31.61236	0.082935
4.00E+00	0.936768	1.94E-04	JUP, PKP2,	366	6	16792	30.58652	0.273848
3.10E+01	7.259953	2.78E-04	S100A8, M	356	717	16881	2.050174	0.12793
1.00E+01	2.34192	3.04E-04	IGLV1-40,	366	99	16792	4.634321	0.394294
1.10E+01	2.576112	3.10E-04	SERPINB5,	366	121	16792	4.170889	0.400598
5.00E+00	1.17096	3.25E-04	DES, KRT1	366	16	16792	14.33743	0.414819
2.20E+01	5.152225	3.73E-04	IGLV1-40,	366	421	16792	2.397518	0.459767
2.50E+01	5.854801	4.99E-04	FGFR2, TF,	383	542	18224	2.194753	0.110264
6.00E+00	1.405152	7.82E-04	JUP, OVOL	366	34	16792	8.096432	0.724697
4.00E+00	0.936768	9.83E-04	JUP, DES, (	383	10	18224	19.0329	0.205527
4.00E+00	0.936768	9.91E-04	EVPL, EPPI	356	10	16881	18.96742	0.386182
9.00E+00	2.107728	0.001084	EPPK1, AL(	366	95	16792	4.346506	0.832687
2.20E+01	5.152225	0.001375	FGFR2, KR	366	466	16792	2.165998	0.896572
3.00E+00	0.702576	0.001393	PKP1, PKP	366	3	16792	45.87978	0.899649
3.00E+00	0.702576	0.001393	KDF1, TP6	366	3	16792	45.87978	0.899649
7.00E+00	1.639344	0.001456	JUP, DES, I	129	67	6879	5.571329	0.20674
4.00E+00	0.936768	0.001476	JUP, PKP2,	366	11	16792	16.68356	0.912483
1.60E+01	3.747073	0.001522	FGFR2, S1(	383	296	18224	2.572013	0.299895
4.00E+00	0.936768	0.001937	COL17A1,	366	12	16792	15.29326	0.959097
7.00E+00	1.639344	0.002054	PRSS8, EN	356	63	16881	5.268727	0.636449
6.00E+00	1.405152	0.002419	JUP, DES, I	383	45	18224	6.344299	0.432585
5.00E+00	1.17096	0.002594	FGFR2, KR	366	27	16792	8.496256	0.986211
9.00E+00	2.107728	0.002621	RGMA, AR	383	113	18224	3.789736	0.458833
3.00E+00	0.702576	0.002746	JUP, PKP2,	366	4	16792	34.40984	0.989274
8.00E+00	1.873536	0.002852	IGLV1-40,	366	87	16792	4.21883	0.990992
1.00E+01	2.34192	0.003068	BGLAP, TN	366	137	16792	3.348889	0.993703
4.00E+00	0.936768	0.003102	KRT6A, SEI	366	14	16792	13.10851	0.994047
6.00E+00	1.405152	0.003114	FGFR2, GD	366	46	16792	5.984319	0.994162
1.50E+01	3.512881	0.00349	F2RL2, TF,	383	291	18224	2.452693	0.558767
4.00E+00	0.936768	0.003816	NTRK1, GA	366	15	16792	12.23461	0.998172
4.00E+00	0.936768	0.003816	KRT6C, KR	366	15	16792	12.23461	0.998172
5.00E+00	1.17096	0.004345	FGFR2, DL	366	31	16792	7.399965	0.999239
6.00E+00	1.405152	0.004958	JUP, CLDN	383	53	18224	5.386669	0.687477
7.00E+00	1.639344	0.005448	LAMA3, IT	129	87	6879	4.290564	0.580486
7.00E+00	1.639344	0.005448	CD19, CR2	129	87	6879	4.290564	0.580486

7.00E+00	1.639344	0.005614	EVPL, SPRI	356	77	16881	4.310776	0.937335
8.00E+00	1.873536	0.006056	IGLV1-40,	356	103	16881	3.682993	0.949636
5.00E+00	1.17096	0.006086	FGFR2, BG	366	34	16792	6.747027	0.999957
6.00E+00	1.405152	0.006745	DLX3, DLX	366	55	16792	5.005067	0.999986
1.00E+01	2.34192	0.00682	FGFR2, SM	356	160	16881	2.963659	0.965504
1.10E+01	2.576112	0.007699	IGLV1-40,	366	186	16792	2.71332	0.999997
6.00E+00	1.405152	0.009049	DLX2, ITGE	366	59	16792	4.66574	1
4.00E+00	0.936768	0.009182	GJB3, GJA1	383	21	18224	9.063285	0.884505
3.00E+00	0.702576	0.009205	KDF1, PTC	366	7	16792	19.66276	1
1.00E+01	2.34192	0.009768	TNFRSF11	366	164	16792	2.797548	1
1.30E+01	3.044496	0.009886	FGFR2, FG	366	254	16792	2.348178	1
6.00E+00	1.405152	0.010383	SH2D3A, S	366	61	16792	4.512765	1
1.00E+01	2.34192	0.01052	JUP, COL1	383	172	18224	2.76641	0.91581
5.00E+00	1.17096	0.010834	ZG16B, TF,	366	40	16792	5.734973	1
7.00E+00	1.639344	0.011084	GATA3, OV	366	86	16792	3.734401	1
3.00E+00	0.702576	0.011287	JUP, COL1	383	8	18224	17.84334	0.929776
1.40E+01	3.278689	0.011588	FGFR2, FG	129	345	6879	2.163937	0.843274
1.90E+01	4.449649	0.011896	BGLAP, CL	366	459	16792	1.899163	1
3.00E+00	0.702576	0.012098	PKP2, GJA1	366	8	16792	17.20492	1
6.00E+00	1.405152	0.013442	BGLAP, TN	366	65	16792	4.235057	1
3.00E+00	0.702576	0.014398	TGM1, TGI	356	9	16881	15.80618	0.999204
4.00E+00	0.936768	0.01493	PRSS8, ST1	383	25	18224	7.613159	0.970401
3.00E+00	0.702576	0.015333	TGM1, TP6	366	9	16792	15.29326	1
3.00E+00	0.702576	0.015333	TACSTD2,	366	9	16792	15.29326	1
3.00E+00	0.702576	0.01775	JUP, PKP2,	356	10	16881	14.22556	0.999851
1.00E+01	2.34192	0.019477	FAM110C,	366	184	16792	2.493466	1
5.00E+00	1.17096	0.020158	FA2H, ELC	366	48	16792	4.779144	1
3.00E+00	0.702576	0.021396	S100A8, S1	356	11	16881	12.93233	0.999976
3.00E+00	0.702576	0.022764	EPPK1, GJA	366	11	16792	12.51267	1
3.00E+00	0.702576	0.022764	TFAP2B, SO	366	11	16792	12.51267	1
5.00E+00	1.17096	0.023356	DES, EVPL,	383	52	18224	4.575216	0.996035
4.00E+00	0.936768	0.024412	TF, CLCA2	383	30	18224	6.344299	0.996921
9.00E+00	2.107728	0.02463	DES, KRT6	366	161	16792	2.564708	1
6.00E+00	1.405152	0.025127	SMOC2, C	383	79	18224	3.613841	0.997407
3.00E+00	0.702576	0.026929	IRF6, FERM	366	12	16792	11.46995	1
1.10E+01	2.576112	0.026936	FGFR2, TF,	383	235	18224	2.227254	0.998321
1.00E+01	2.34192	0.027301	ABCA10, S	356	202	16881	2.347452	0.999999
1.00E+01	2.34192	0.027787	SMOC2, TI	366	196	16792	2.340805	1
1.10E+01	2.576112	0.027938	DLX3, EBF2	356	236	16881	2.210186	0.999999
4.00E+00	0.936768	0.029206	FAM3B, IL1	366	31	16792	5.919972	1
5.00E+00	1.17096	0.029996	SH2D3A, S	356	56	16881	4.233798	1
6.00E+00	1.405152	0.030148	KRT6A, S10	366	80	16792	3.440984	1
6.00E+00	1.405152	0.031581	FGFR2, TN	366	81	16792	3.398502	1
5.00E+00	1.17096	0.033284	FGFR2, EPI	366	56	16792	4.096409	1
3.00E+00	0.702576	0.033965	GJB3, GJA1	356	14	16881	10.16112	1
4.00E+00	0.936768	0.034119	CST2, CST1	356	34	16881	5.578652	1
3.00E+00	0.702576	0.036087	KDF1, EPP	366	14	16792	9.831382	1
3.00E+00	0.702576	0.036087	DNER, REL	366	14	16792	9.831382	1
6.00E+00	1.405152	0.036132	TNFAIP8L3	366	84	16792	3.277127	1
9.00E+00	2.107728	0.036446	PROM2, M	383	180	18224	2.379112	0.999831
4.00E+00	0.936768	0.037084	FGFR2, PD	366	34	16792	5.397621	1
4.00E+00	0.936768	0.039927	JUP, PKP2,	366	35	16792	5.243404	1
1.00E+01	2.34192	0.040293	TNFRSF11	356	217	16881	2.185186	1
1.00E+01	2.34192	0.040469	ATP2C2, C	366	210	16792	2.184751	1
9.00E+00	2.107728	0.041002	IGLV1-40,	366	178	16792	2.319764	1
3.00E+00	0.702576	0.041052	KRT16, KRT	366	15	16792	9.175956	1
5.00E+00	1.17096	0.041344	LTF, TP63,	366	60	16792	3.823315	1
5.00E+00	1.17096	0.041362	JUP, PKP3,	356	62	16881	3.824076	1

4.00E+00	0.936768	0.042877	BGLAP, S1	366	36	16792	5.097753	1
2.00E+00	0.468384	0.043002	NTRK1, DN	366	2	16792	45.87978	1
2.00E+00	0.468384	0.043002	EPHB6, GA	366	2	16792	45.87978	1
2.00E+00	0.468384	0.043002	S100A7, S1	366	2	16792	45.87978	1
2.00E+00	0.468384	0.043002	FGFR2, TP(	366	2	16792	45.87978	1
2.00E+00	0.468384	0.043002	FGFR2, SEI	366	2	16792	45.87978	1
2.00E+00	0.468384	0.043002	S100A8, S1	366	2	16792	45.87978	1
6.00E+00	1.405152	0.046392	COL17A1,	366	90	16792	3.058652	1
5.00E+00	1.17096	0.048052	AKR1B10,	366	63	16792	3.641252	1
6.00E+00	1.405152	0.05016	FGFR2, CC	366	92	16792	2.99216	1
1.30E+01	3.044496	0.050614	BGLAP, LY	383	335	18224	1.846475	0.999995
4.00E+00	0.936768	0.051426	SPINT2, PI	356	40	16881	4.741854	1
3.00E+00	0.702576	0.051687	SERPINB3,	366	17	16792	8.096432	1
4.00E+00	0.936768	0.05236	CLDN4, DS	366	39	16792	4.705619	1
5.00E+00	1.17096	0.052455	COMP, ELI	356	67	16881	3.538697	1
5.00E+00	1.17096	0.052838	BGLAP, IL1	366	65	16792	3.529214	1
8.00E+00	1.873536	0.055471	DKK1, GDI	356	162	16881	2.341656	1
4.00E+00	0.936768	0.055727	CA6, CST2	366	40	16792	4.587978	1
1.80E+01	4.215457	0.056849	TRIM29, SI	366	513	16792	1.609817	1
3.00E+00	0.702576	0.057333	EBF2, GAT.	366	18	16792	7.64663	1
3.00E+00	0.702576	0.057333	BGLAP, ST	366	18	16792	7.64663	1
4.00E+00	0.936768	0.057538	JUP, ATP2(	383	42	18224	4.531642	0.999999
7.00E+00	1.639344	0.058931	IGLV1-40,	366	127	16792	2.528807	1
1.30E+01	3.044496	0.061207	FGFR2, FG	129	393	6879	1.763951	0.999956
2.00E+00	0.468384	0.061775	ADCYAP1I	356	3	16881	31.61236	1
2.00E+00	0.468384	0.061775	AKR1B10, .	356	3	16881	31.61236	1
6.00E+00	1.405152	0.06194	COMP, CC	356	101	16881	2.816943	1
7.00E+00	1.639344	0.062029	FZD10, GD	129	151	6879	2.472047	0.999962
4.00E+00	0.936768	0.062762	FGFR2, BG	366	42	16792	4.369503	1
1.00E+01	2.34192	0.063028	RGMA, SYI	383	237	18224	2.00769	1
2.00E+00	0.468384	0.063806	TP63, WN	366	3	16792	30.58652	1
2.00E+00	0.468384	0.063806	S100A8, S1	366	3	16792	30.58652	1
2.00E+00	0.468384	0.063806	SIX2, LTF	366	3	16792	30.58652	1
2.00E+00	0.468384	0.063806	TACSTD2,	366	3	16792	30.58652	1
2.00E+00	0.468384	0.063806	SPINT2, ST	366	3	16792	30.58652	1
2.00E+00	0.468384	0.063806	TGM1, TGI	366	3	16792	30.58652	1
5.00E+00	1.17096	0.06589	GPX2, GPX	366	70	16792	3.277127	1
5.00E+00	1.17096	0.06589	FZD10, KR	366	70	16792	3.277127	1
4.00E+00	0.936768	0.066426	BGLAP, NT	366	43	16792	4.267887	1
3.00E+00	0.702576	0.067408	MME, ANF	129	23	6879	6.955511	0.999985
1.20E+01	2.810304	0.068995	BGLAP, TN	366	304	16792	1.811044	1
7.00E+00	1.639344	0.069515	CYP4X1, T	356	137	16881	2.422845	1
1.90E+01	4.449649	0.070959	FGFR2, S1(	366	567	16792	1.537418	1
3.00E+00	0.702576	0.077297	GPX3, PTG	356	22	16881	6.466164	1
4.00E+00	0.936768	0.078711	FZD10, WI	129	54	6879	3.950043	0.999998
1.30E+01	3.044496	0.078774	NPTX1, SY	366	348	16792	1.7139	1
6.00E+00	1.405152	0.078892	DNER, GA	366	105	16792	2.621702	1
6.00E+00	1.405152	0.078892	BGLAP, S1	366	105	16792	2.621702	1
4.00E+00	0.936768	0.079805	KRT5, KRT	356	48	16881	3.951545	1
2.00E+00	0.468384	0.081252	SPINK5, AI	383	4	18224	23.79112	1
2.00E+00	0.468384	0.081509	S100A8, S1	356	4	16881	23.70927	1
3.00E+00	0.702576	0.081831	CCL21, CX	366	22	16792	6.256334	1
3.00E+00	0.702576	0.083521	FGFR2, FG	356	23	16881	6.185027	1
5.00E+00	1.17096	0.08355	FGFR2, PD	366	76	16792	3.018407	1
5.00E+00	1.17096	0.08355	RGMA, CH	366	76	16792	3.018407	1
4.00E+00	0.936768	0.083746	CXCL5, CX	356	49	16881	3.870901	1
2.00E+00	0.468384	0.084159	ADCYAP1I	366	4	16792	22.93989	1
2.00E+00	0.468384	0.084159	TFCP2L1, (	366	4	16792	22.93989	1

2.00E+00	0.468384	0.084159	KRT13, TB)	366	4	16792	22.93989	1
2.00E+00	0.468384	0.084159	S100A8, S1	366	4	16792	22.93989	1
2.00E+00	0.468384	0.084159	FGFR2, GD	366	4	16792	22.93989	1
2.00E+00	0.468384	0.084159	STAP1, CY	366	4	16792	22.93989	1
2.00E+00	0.468384	0.084159	S100A8, S1	366	4	16792	22.93989	1
8.00E+00	1.873536	0.088924	LAMA3, IT	129	206	6879	2.070896	1
6.00E+00	1.405152	0.09011	MMP10, C	356	113	16881	2.517799	1
1.30E+01	3.044496	0.092275	TCHH, S10	383	371	18224	1.667302	1
4.00E+00	0.936768	0.092925	CYP11A1,	129	58	6879	3.677626	1
5.00E+00	1.17096	0.093164	GATA3, CN	366	79	16792	2.903784	1
3.00E+00	0.702576	0.095078	DLX2, TP6	366	24	16792	5.734973	1
5.00E+00	1.17096	0.096481	CHRDL1, L	366	80	16792	2.867486	1
4.00E+00	0.936768	0.099027	ELFN2, LM	366	51	16792	3.598414	1

Benjamini	FDR
7.88E-15	8.03E-15
9.15E-14	1.89E-13
6.11E-14	1.89E-13
5.46E-14	1.55E-13
2.78E-12	1.53E-11
2.30E-12	2.53E-11
1.54E-11	2.54E-10
2.38E-10	5.23E-09
3.14E-10	8.62E-09
8.58E-10	2.83E-08
1.38E-08	5.64E-08
1.33E-08	6.75E-08
5.81E-06	3.40E-05
1.88E-06	7.24E-05
4.71E-05	2.88E-04
5.64E-04	0.004942
1.51E-04	0.006631
0.002866	0.02047
0.00408	0.033324
6.74E-04	0.033338
0.004539	0.053131
0.001103	0.060592
0.017166	0.25251
0.03493	0.325591
0.022556	0.398947
0.0489	0.509655
0.045463	0.520262
0.043671	0.544603
0.046261	0.625576
0.010565	0.639796
0.088018	1.305971
0.01899	1.256056
0.067347	1.415153
0.112363	1.805613
0.13221	2.285787
0.126494	2.315856
0.126494	2.315856
0.20674	1.741636
0.126572	2.451991
0.027052	1.939648
0.154849	3.205256
0.118808	2.911611
0.039668	3.065229
0.192807	4.271919
0.040109	3.317137
0.194229	4.516712
0.192712	4.686417
0.197749	5.033593
0.192244	5.087959
0.18595	5.10678
0.049851	4.39547
0.215329	6.223354
0.215329	6.223354
0.233518	7.056591
0.066128	6.189949
0.352301	6.377324
0.352301	6.377324

0.264917	7.770498
0.258328	8.357222
0.301971	9.749888
0.319435	10.75077
0.263675	9.36441
0.34611	12.18031
0.383405	14.1671
0.113007	11.18256
0.379072	14.39375
0.387669	15.20702
0.382374	15.37773
0.388443	16.08836
0.122121	12.7119
0.392829	16.72805
0.391483	17.08122
0.124363	13.57743
0.460845	13.11551
0.405084	18.21897
0.402288	18.49895
0.427605	20.34315
0.448222	18.80942
0.154325	17.58358
0.462837	22.86964
0.462837	22.86964
0.492262	22.68739
0.538027	28.14737
0.542027	28.98181
0.532366	26.70997
0.578097	32.08951
0.578097	32.08951
0.222268	26.20091
0.222322	27.21947
0.599024	34.23658
0.219733	27.90263
0.624157	36.79368
0.225535	29.60413
0.596637	32.81401
0.62795	37.72416
0.581596	33.44311
0.63879	39.23392
0.585796	35.43943
0.643059	40.21727
0.652969	41.6844
0.665298	43.38451
0.611133	39.13338
0.592998	39.27271
0.688243	46.08049
0.688243	46.08049
0.681779	46.12323
0.284047	37.95375
0.684613	47.00984
0.705252	49.57892
0.63641	44.61741
0.703717	50.05492
0.702154	50.5193
0.696318	50.5625
0.692755	50.81552
0.628297	45.49714

0.700135	52.1207
0.695229	52.22526
0.695229	52.22526
0.695229	52.22526
0.695229	52.22526
0.695229	52.22526
0.695229	52.22526
0.717316	54.9915
0.724442	56.28976
0.734445	57.88814
0.362466	48.71264
0.692933	53.16529
0.739815	59.0119
0.739123	59.4981
0.684177	53.89001
0.737119	59.83974
0.689604	55.95454
0.751049	61.8498
0.753095	62.6043
0.751138	62.9254
0.751138	62.9254
0.390577	53.31868
0.756023	63.96782
0.918782	53.31919
0.714896	59.99494
0.714896	59.99494
0.701794	60.09613
0.869496	53.80969
0.773385	66.35485
0.408626	56.69632
0.774483	66.97955
0.774483	66.97955
0.774483	66.97955
0.774483	66.97955
0.774483	66.97955
0.774483	66.97955
0.781046	68.19339
0.781046	68.19339
0.779365	68.49837
0.842665	56.90562
0.787998	69.92419
0.730962	64.48406
0.793247	70.97259
0.756732	68.52142
0.844664	62.80003
0.823533	74.81461
0.820096	74.86863
0.820096	74.86863
0.756106	69.72854
0.483666	66.35954
0.752012	70.52386
0.827913	76.18262
0.749475	71.43803
0.830719	76.92123
0.830719	76.92123
0.739388	71.53877
0.82931	77.17778
0.82931	77.17778

0.82931	77.17778
0.82931	77.17778
0.82931	77.17778
0.82931	77.17778
0.82931	77.17778
0.842911	67.48011
0.75534	74.25078
0.518471	71.19466
0.821475	69.16134
0.856714	80.66957
0.859319	81.34388
0.860304	81.82397
0.864598	82.66586
