# Supplementary material for: Identification of tumor mutation burden-related hub genes and the underlying mechanism in melanoma
Source: J Cancer. 2021 Mar 1;12(8):2440–9. doi: 10.7150/jca.53697 (PMC7974884; doi:10.7150/jca.53697)
Supplement: Supplementary file 1 — Supplementary tables. [file jcav12p2440s1.zip › Table-S2-degOfTMB.pdf]

	logFC	PValue
ZG16B	-3.75708	5.27E-40
FGFR3	2.988588	3.94E-37
TCHH	2.867399	7.74E-37
TG	-3.36967	5.14E-36
CNFN	3.345955	1.41E-35
TGM1	3.214047	2.04E-31
SULT2B1	2.950871	7.30E-30
ENTPD3	2.687665	1.12E-29
SLC22A1	2.189735	2.33E-29
TBX1	2.157011	4.17E-27
VIPR1	2.085718	7.28E-26
TREX2	1.730211	1.07E-25
LYPD3	2.724212	4.44E-25
EPHX3	2.490928	1.65E-24
ASPRV1	2.567797	9.20E-24
CSTA	2.338223	9.82E-24
C6orf132	2.179441	9.90E-24
CYSRT1	1.784966	2.08E-23
MTND1P23	2.039616	2.33E-23
TUBA4A	1.773296	2.39E-23
LYPD5	1.768247	2.52E-23
S100A9	2.548583	2.90E-23
EPPK1	2.612878	7.31E-23
CLIC3	2.018211	1.39E-22
FAM107A	1.634543	1.93E-22
OVOL1	2.778937	2.63E-22
NPTX1	2.277836	3.14E-22
ADAMTS8	-2.21141	4.17E-22
CRYBG2	2.32877	8.47E-22
BNIPL	2.132562	8.48E-22
PI3	3.69688	9.04E-22
PIGR	-2.89418	3.58E-21
TMEM45B	1.915454	4.72E-21
MASP1	1.907091	5.44E-21
F5	2.298322	6.30E-21
GRHL3	2.21204	6.46E-21
DEGS2	2.000124	8.08E-21
KLK13	3.020499	9.71E-21
GPX2	2.189936	1.71E-20
SOX15	1.620579	1.95E-20
JUP	1.493668	2.68E-20
TPTEP1	1.67835	3.10E-20
KRT17	3.428787	4.10E-20
KLK10	3.122405	4.78E-20
AQP3	2.101088	1.28E-19
TNS4	2.479818	1.36E-19
HAS3	1.470278	1.65E-19
ESRP2	2.112245	1.87E-19
ALDH3A1	1.836215	1.88E-19
KCNK7	1.515736	1.89E-19
DLX3	2.215814	2.13E-19
TENM2	2.324726	2.50E-19
EVPL	2.606706	2.53E-19
NCCRP1	2.486916	2.63E-19
PPL	1.871944	2.70E-19
CHI3L1	2.094752	2.83E-19
S100A8	2.582032	3.24E-19

ZBTB7C	1.780002	4.17E-19
PLEKHG6	1.618337	4.38E-19
GSDMA	2.111344	4.46E-19
ENDOU	2.016118	5.05E-19
SUSD4	1.698951	7.23E-19
LYNX1	1.519991	8.88E-19
DSC2	1.891983	1.20E-18
TMEM184A	2.128506	1.36E-18
IL20RB	1.678755	1.43E-18
PROM2	2.305111	1.71E-18
TACSTD2	2.241155	2.12E-18
ATP12A	2.511182	2.23E-18
KRT15	2.855693	2.36E-18
TTC22	1.91926	2.65E-18
TGM3	2.762216	2.87E-18
RHOV	2.093266	3.48E-18
TP63	2.053258	3.74E-18
FGFR2	1.979085	5.30E-18
LAD1	1.912938	1.10E-17
SFN	3.073979	1.13E-17
MAP3K8	1.108305	1.28E-17
SGPP2	1.281346	2.31E-17
NECTIN4	1.804953	2.33E-17
SMPD3	1.203808	2.35E-17
THEM5	1.517216	2.67E-17
KLHL41	-1.31841	3.63E-17
BICDL2	2.236348	3.69E-17
ANKRD35	1.433382	4.56E-17
DES	-2.39439	6.43E-17
C1orf116	1.767154	6.64E-17
CA6	-3.36504	7.01E-17
MRGPRX4	-2.34198	8.24E-17
SBSN	2.957987	9.17E-17
A2ML1	2.545946	9.27E-17
IL1RN	1.448835	9.32E-17
PTCH2	1.240771	9.76E-17
RRAD	-1.67891	1.24E-16
CXCL14	1.968097	1.34E-16
S100A2	2.125399	1.35E-16
S100A14	2.645365	1.61E-16
KRT10	1.976515	1.98E-16
SMOC2	1.183301	3.59E-16
RAB25	2.700474	4.80E-16
ABCA12	2.100151	4.84E-16
SPINK5	2.221083	6.10E-16
FLG	2.063121	6.22E-16
NOTUM	1.927779	8.23E-16
CCDC3	1.250035	8.47E-16
SCX	-1.56843	1.04E-15
RHCG	2.111498	1.09E-15
EPHA1	1.277185	1.37E-15
PLEKHS1	-1.99669	1.54E-15
IGLV9-49	2.756909	2.36E-15
PRSS27	1.204405	2.52E-15
SH2D3A	1.068347	3.24E-15
TMPRSS9	1.922495	3.75E-15
PKP3	2.197479	3.82E-15
CXCL5	-2.52752	4.14E-15

ATP2C2	1.491682	4.33E-15
COL17A1	2.379483	4.71E-15
SEC14L6	2.011663	7.02E-15
DUOX1	1.632965	7.21E-15
PKP1	2.525927	7.42E-15
MYOZ2	-2.16345	7.45E-15
GJB2	1.948938	8.47E-15
CDA	1.554924	9.09E-15
SCNN1B	1.861924	9.69E-15
PTGS1	1.078339	9.75E-15
ZNF750	2.261348	1.00E-14
LRRC17	-1.67717	1.05E-14
TRIM29	2.823423	1.22E-14
ALX4	1.971085	1.25E-14
HTN1	-3.40419	1.27E-14
GJB3	2.318711	1.50E-14
RASSF10	1.692195	1.75E-14
ELOVL3	1.681971	1.88E-14
EPHB6	1.309673	2.00E-14
TGM5	2.138183	2.49E-14
GPX3	1.405522	2.87E-14
SPRR2D	2.918371	2.87E-14
ST14	1.181813	2.97E-14
MTRNR2L8	-1.24933	3.23E-14
ACKR2	1.016006	3.47E-14
NTRK1	1.31295	3.58E-14
GRB7	1.66797	3.63E-14
LAMA3	1.170438	3.82E-14
HGF	-1.49525	3.84E-14
FZD10	1.377469	4.29E-14
CCBE1	-1.58906	4.34E-14
DUOXA1	2.123116	4.38E-14
KLHL14	1.614103	4.54E-14
HOPX	1.342154	6.00E-14
CHAD	2.083148	6.48E-14
TNNI2	-1.25696	7.58E-14
KIRREL3	-1.18454	8.92E-14
SP6	1.192345	1.04E-13
SPINT2	1.124532	1.40E-13
SYTL1	1.096547	1.41E-13
REG3G	2.607743	1.62E-13
DSP	1.884077	2.19E-13
CYP11A1	-2.09559	2.29E-13
IRF6	1.582987	2.32E-13
FAM83A	2.668175	3.14E-13
CLCA2	2.176196	3.20E-13
VWA2	1.958447	4.52E-13
CLDN4	1.632694	5.22E-13
PALMD	1.017028	6.57E-13
GRHL1	1.243029	8.45E-13
MTCO1P40	1.181255	8.51E-13
WFDC21P	1.32146	9.92E-13
DNER	-2.2093	1.18E-12
WNT6	1.333962	1.57E-12
ALOX15B	1.418426	1.57E-12
ADCYAP1R1	1.169696	1.77E-12
ANO9	1.172397	1.86E-12
AP002761.4	1.380611	1.88E-12

NIPAL4	1.787727	1.99E-12
KRT14	2.936387	2.24E-12
LMTK3	1.068761	2.38E-12
TNFRSF11B	1.356754	2.41E-12
SLC5A9	1.156832	2.84E-12
SCNN1A	1.590001	2.84E-12
GRB14	-1.49814	3.20E-12
LGR6	1.303477	4.02E-12
KRT80	1.805482	4.35E-12
AC115522.1	1.243487	4.38E-12
RNF39	1.07994	4.72E-12
TMEM200C	1.462515	4.83E-12
EBF2	1.151627	5.87E-12
PDZK1IP1	2.405557	6.42E-12
SLPI	2.200183	6.86E-12
ADAM33	1.064663	7.37E-12
SLIT3	1.017246	7.37E-12
HOXA9	1.161533	8.32E-12
BMP6	1.16276	1.19E-11
CA2	1.012591	1.20E-11
PNMA3	1.341957	1.38E-11
TNFAIP8L3	1.01902	1.40E-11
MYPN	-1.57715	1.49E-11
CRABP2	1.356026	1.50E-11
P2RY2	1.161479	1.61E-11
LINC00926	1.131173	1.64E-11
GSDMC	1.543517	1.81E-11
AKR1B10	2.408622	1.81E-11
PRSS8	1.928383	1.93E-11
LRATD1	1.281308	1.93E-11
GDF7	1.123594	1.97E-11
MALL	1.025369	2.21E-11
ADGRD1	1.227265	2.45E-11
KRT5	2.575717	2.46E-11
FA2H	1.523375	2.65E-11
MLC1	1.690338	2.69E-11
CD177	1.596736	2.78E-11
BGLAP	-1.03969	3.03E-11
FUT2	1.004292	3.14E-11
SPRR2A	3.407875	3.14E-11
KRT6B	2.90262	3.16E-11
IGF2	-1.26315	3.81E-11
WNT11	1.198546	4.65E-11
RGMA	1.052155	5.23E-11
KDF1	1.625814	5.36E-11
ABCA10	1.069557	5.47E-11
ANPEP	-1.1869	5.92E-11
KRTDAP	2.925706	6.69E-11
SH3RF2	1.630142	6.81E-11
AP1M2	1.750108	7.86E-11
GBP6	1.527184	8.41E-11
AKR1C1	1.151328	8.95E-11
CYP4X1	1.283426	9.30E-11
GJA1	1.004183	1.05E-10
DSC3	2.423857	1.05E-10
CAMSAP3	1.357231	1.25E-10
PLCH2	1.061367	1.45E-10
WSCD1	1.109828	1.53E-10

G0S2	-1.17565	1.54E-10
IGHV1OR15-2	1.881849	1.58E-10
ASPG	1.590006	1.60E-10
CXCL3	-1.28057	1.63E-10
MMP10	1.797149	1.87E-10
ADARB2	1.388307	2.03E-10
CDHR1	1.441654	2.18E-10
LY6D	2.953719	2.28E-10
KLC3	1.521445	2.32E-10
PTN	1.72231	2.73E-10
S100A7	2.999425	2.74E-10
KRT27	1.669862	2.86E-10
SLC38A5	1.02137	3.37E-10
GATA3	1.048019	3.52E-10
SPRR2E	3.392569	3.67E-10
CAMK1G	-1.31877	3.72E-10
LARGE2	1.314295	3.81E-10
NEBL	1.232057	3.95E-10
RHOD	1.182982	4.04E-10
LINC02251	1.306648	4.46E-10
DSG3	2.530396	4.50E-10
FGFBP1	2.915932	5.06E-10
SPRR1A	3.373686	5.18E-10
NIBAN3	1.301008	5.95E-10
FAM3B	-1.1645	6.61E-10
KCP	-1.11002	9.08E-10
DACH1	1.142749	9.57E-10
MAL2	1.65935	1.12E-09
KRT16	2.944892	1.39E-09
LINC01537	-1.43616	1.51E-09
CNTN2	1.21915	1.72E-09
SIX2	-1.24153	2.00E-09
TDO2	-1.10642	2.26E-09
DMKN	1.452983	2.52E-09
NKPD1	1.209289	2.91E-09
IGKV6-21	2.303046	3.12E-09
FAM160A1	1.069337	3.14E-09
TFCP2L1	1.207035	3.15E-09
KRT86	1.434021	3.36E-09
PAEP	-2.09843	3.51E-09
BBOX1	1.689866	3.92E-09
KRT6A	2.8168	4.13E-09
MTCO3P12	1.326417	4.57E-09
PITX1	1.630616	5.26E-09
BSPRY	1.138272	5.72E-09
MYOM3	1.013242	6.01E-09
HCAR2	1.080798	6.11E-09
APCDD1	1.207765	6.36E-09
SNED1	1.010826	7.27E-09
GJB6	1.986534	1.16E-08
IGHV4-59	1.445422	1.25E-08
PDPN	-1.02743	1.40E-08
SLC26A4-AS1	1.356904	1.41E-08
ZBTB16	1.019774	1.43E-08
BANK1	1.275043	1.48E-08
CYP19A1	1.367277	1.53E-08
SPRR1B	3.021131	1.68E-08
LTF	-1.36531	1.78E-08

SLC6A11	1.703205	1.80E-08
RHEX	1.008121	1.88E-08
AREG	-1.49667	2.04E-08
CALML3	2.975643	2.18E-08
NAPSA	-1.10515	2.19E-08
ELN	1.006491	2.19E-08
CEND1	-1.07146	2.44E-08
IVL	2.554105	2.84E-08
KLK1	-1.28921	2.90E-08
APCDD1L	-1.46255	2.92E-08
KRT6C	2.995673	3.06E-08
SERPINB3	2.662093	3.11E-08
PRSS22	1.448653	3.52E-08
AL035665.1	-1.5114	3.80E-08
SERPINB5	2.354749	3.99E-08
ITGB8	-1.0481	4.09E-08
IGHV3-64	1.682092	4.15E-08
KRT23	1.570927	4.24E-08
COMP	1.279238	4.67E-08
CPXM2	1.106028	4.79E-08
ITIH5	1.407154	4.90E-08
IGKV1-27	1.591135	4.99E-08
CTSE	-1.15524	5.57E-08
LINC01215	1.281478	5.76E-08
HSPB7	1.087606	5.95E-08
VAT1L	-1.71192	6.08E-08
MMP1	-1.37933	6.26E-08
KRT1	2.598263	6.99E-08
SYNPR	1.84795	8.60E-08
SLC16A6	-1.18311	8.79E-08
WFDC1	-1.32069	8.94E-08
FAM181B	1.064015	9.56E-08
IGHV4-28	1.392597	1.02E-07
EHF	1.233562	1.05E-07
TNN	1.124812	1.34E-07
PPP2R2C	1.460984	1.37E-07
SCN3A	1.041676	1.54E-07
UTS2B	-1.01778	1.57E-07
LINC01502	-2.05814	1.64E-07
MME	-1.17052	1.67E-07
PKP2	1.044128	1.71E-07
TCN1	1.371974	1.82E-07
HIF3A	1.352371	1.94E-07
FAM110C	1.243666	2.04E-07
DNASE1L3	1.024074	2.11E-07
HOXC13-AS	1.077109	2.16E-07
AC005330.1	1.159382	2.49E-07
PLAAT5	1.380292	2.52E-07
IGSF1	-1.45749	2.52E-07
TMPRSS3	1.069435	2.65E-07
TPRXL	1.05797	2.74E-07
SEMA3D	1.076019	2.75E-07
MS4A1	1.352036	2.91E-07
STC1	-1.07415	3.00E-07
SDR42E1	1.031603	3.02E-07
COL4A6	1.147978	3.17E-07
CHRDL1	1.257622	3.49E-07
CD24	1.175398	3.87E-07

F2RL2	1.13067	4.34E-07
SPRR3	2.063779	4.61E-07
STAP1	1.034522	4.68E-07
KCNK3	-1.13919	5.55E-07
FSD1	-1.02313	5.69E-07
EMILIN3	1.185616	6.04E-07
KIRREL2	1.090315	7.70E-07
GLB1L3	1.20017	8.27E-07
PI16	1.278896	9.02E-07
DSG1	2.15233	9.22E-07
SCEL	1.42696	9.34E-07
CNTFR	1.260805	9.73E-07
TF	1.237253	9.76E-07
PYY2	1.094288	9.85E-07
GAL	1.30574	1.07E-06
IGKV1D-13	1.621316	1.11E-06
IGHV1-2	1.374601	1.34E-06
FUT3	1.232826	1.42E-06
FCRL1	1.342894	1.69E-06
SULT1B1	-1.00487	1.70E-06
IGLV4-60	1.61811	1.73E-06
CNR2	1.124784	2.28E-06
PAX5	1.19021	2.47E-06
TPSB2	1.004518	2.62E-06
IL22RA1	1.150604	2.77E-06
LCE2A	1.430419	2.97E-06
DKK1	-1.08599	3.11E-06
AL139241.1	1.010165	3.30E-06
RIMS2	1.093437	3.67E-06
NPY1R	1.204847	3.84E-06
IGLV10-54	1.429413	4.14E-06
BFSP2	1.045423	4.18E-06
KRT13	1.797828	4.29E-06
EPYC	-1.3551	4.73E-06
GAP43	-1.29872	5.53E-06
IGLV2-8	1.196388	5.65E-06
NUDT10	1.030721	6.71E-06
IGHV3-48	1.216675	6.92E-06
ART3	1.049622	7.23E-06
IGKV2D-29	1.390051	8.78E-06
IGHJ3	1.161369	8.87E-06
CST2	-1.30917	9.78E-06
IRX2	1.011787	1.03E-05
DLX2	-1.01901	1.06E-05
FAM30A	1.052032	1.13E-05
WDR72	-1.03105	1.16E-05
IGHV3-73	-1.43049	1.16E-05
TFPI2	-1.06305	1.27E-05
AL035425.3	1.282746	1.48E-05
CD19	1.008616	1.70E-05
GRIN2B	1.052846	1.76E-05
IGLV1-47	1.133819	1.90E-05
CST1	1.389598	1.91E-05
FCRL2	1.019394	1.94E-05
LINC01705	-1.15238	2.12E-05
LBP	1.017128	2.17E-05
SLC38A11	1.046004	2.52E-05
CHRM1	1.022853	2.66E-05

ASB11	1.077031	3.43E-05
IGKV1-17	1.164465	3.64E-05
IGHV4-39	1.046663	4.87E-05
ELFN2	1.102271	5.03E-05
NTSR1	-1.23471	5.89E-05
IGHM	1.054505	6.02E-05
IGHV4-61	1.165541	6.09E-05
TCL1A	1.049332	6.15E-05
CR2	1.296796	6.18E-05
IGLV8-61	1.231883	7.10E-05
AC104129.1	1.133348	7.69E-05
IGLV1-40	1.049821	7.72E-05
IGLV4-69	1.132727	7.92E-05
IGKV1OR2-108	1.058896	8.21E-05
LRRTM1	1.363618	8.65E-05
VIT	1.1242	8.72E-05
RELN	1.085155	8.81E-05
FCER2	1.052102	9.44E-05
CTNND2	1.159814	0.000109
CHIT1	1.040116	0.000111
NEFL	-1.23541	0.000122
AL390755.1	1.398261	0.000124
TEKT5	1.017707	0.000126
CCL21	1.011443	0.000164
NDST3	1.013218	0.000189
SERPINB7	-1.29874	0.000223
FERMT1	1.00138	0.000248
CRABP1	-1.32044	0.000252
AC124804.1	1.096844	0.000316
ALDH3B2	1.01275	0.000407
BPIFB1	1.140143	0.000791
IGLV3-16	1.084889	0.000853
TFAP2B	1.262123	0.000881
CEACAM6	1.119525	0.001181
IGKV1D-16	1.003849	0.001626
DSCR4	-1.02547	0.003457
MAGEA4	-1.32547	0.005063
KRT4	1.065725	0.007463
