# Supplementary material for: Identification of tumor mutation burden-related hub genes and the underlying mechanism in melanoma
Source: J Cancer. 2021 Mar 1;12(8):2440–9. doi: 10.7150/jca.53697 (PMC7974884; doi:10.7150/jca.53697)
Supplement: Supplementary file 1 — Supplementary tables. [file jcav12p2440s1.zip › Table-S1-TMB.pdf]

	TMB
TCGA-DA-A95X	11.63514
TCGA-W3-A825	23.43243
TCGA-EE-A29L	42.74324
TCGA-BF-A5ER	3.837838
TCGA-D3-A8GP	12.89189
TCGA-D3-A2J9	0.864865
TCGA-EB-A299	3.243243
TCGA-GN-A9SD	2.756757
TCGA-GF-A4EO	4.013514
TCGA-EE-A184	7.5
TCGA-FS-A1ZA	25.71622
TCGA-EE-A2MC	14.81081
TCGA-EE-A2GI	33.02703
TCGA-EE-A2MN	3.27027
TCGA-D3-A8GR	8.108108
TCGA-EB-A82B	0.864865
TCGA-FR-A69P	4.797297
TCGA-DA-A3F5	6.783784
TCGA-BF-AAP2	27.27027
TCGA-GF-A6C8	7.851351
TCGA-D3-A8GB	18.58108
TCGA-GN-A26C	37.95946
TCGA-FS-A1ZH	0.621622
TCGA-RP-A694	15.06757
TCGA-D9-A1JW	18.52703
TCGA-FR-A7U8	0.756757
TCGA-FS-A1ZD	5.391892
TCGA-EB-A3XD	2.635135
TCGA-EE-A180	11.81081
TCGA-BF-AAP1	17.85135
TCGA-FS-A1ZZ	32.21622
TCGA-ER-A19G	11.37838
TCGA-DA-A1I5	9.608108
TCGA-D3-A2JP	6.256757
TCGA-EE-A2GM	15.24324
TCGA-D3-A3MO	1.459459
TCGA-D3-A5GO	32.68919
TCGA-YG-AA3P	1.567568
TCGA-EE-A3J3	3.527027
TCGA-FS-A1ZC	14.12162
TCGA-EE-A2MH	2.472973
TCGA-EE-A2GE	4.648649
TCGA-EE-A2GH	6.77027
TCGA-D3-A2JH	11.52703
TCGA-D3-A2JE	6.743243
TCGA-EE-A2GP	12.90541
TCGA-FR-A726	144.4459
TCGA-D3-A2JB	0.324324
TCGA-EE-A3AF	22.52703
TCGA-XV-A9W2	0.797297
TCGA-D3-A1Q1	1.162162
TCGA-EB-A3XF	8.216216
TCGA-EB-A5VU	0.878378
TCGA-EE-A2GB	13.86486
TCGA-EE-A2MT	20.18919
TCGA-EE-A3AC	22.08108
TCGA-EB-A44O	10.55405

TCGA-FW-A5DX	4.297297
TCGA-ER-A196	0.445946
TCGA-ER-A19K	7.918919
TCGA-DA-A1HV	29.28378
TCGA-D3-A2JD	7.864865
TCGA-EE-A20H	13.12162
TCGA-EE-A29C	8.824324
TCGA-D3-A3MR	14.95946
TCGA-ER-A3EV	1.621622
TCGA-D9-A3Z3	2.797297
TCGA-QB-A6FS	23.58108
TCGA-WE-A8ZO	7.675676
TCGA-Z2-A8RT	62.95946
TCGA-XV-AAZW	3.243243
TCGA-EB-A44Q	0.986486
TCGA-D3-A2JC	4.513514
TCGA-GN-A4U7	4.905405
TCGA-EB-A5SE	5.756757
TCGA-D3-A8GN	3.959459
TCGA-FS-A4F0	12.04054
TCGA-ER-A42K	4.243243
TCGA-EE-A2M5	43.10811
TCGA-ER-A19N	9.608108
TCGA-ER-A2NE	2.837838
TCGA-W3-AA21	11.05405
TCGA-BF-AAP8	0.148649
TCGA-D3-A2JG	2.297297
TCGA-GN-A264	1.067568
TCGA-D3-A3BZ	0.418919
TCGA-EB-A44P	6.851351
TCGA-FS-A1ZY	0.648649
TCGA-GN-A8LN	5.581081
TCGA-BF-A3DM	9.594595
TCGA-FS-A1ZT	3.594595
TCGA-D3-A3C3	5.337838
TCGA-EE-A2M6	11.63514
TCGA-GN-A4U3	6.810811
TCGA-D3-A5GT	0.594595
TCGA-D3-A2JL	6.77027
TCGA-EB-A3XB	10.71622
TCGA-ER-A42L	10.09459
TCGA-FS-A1ZG	0.716216
TCGA-DA-A1I7	9.702703
TCGA-FS-A4F5	16.35135
TCGA-BF-A5EQ	12.22973
TCGA-GN-A269	13.86486
TCGA-EE-A3AA	26.75676
TCGA-FW-A3R5	379.6757
TCGA-GN-A4U5	6.972973
TCGA-EE-A20C	45.01351
TCGA-FW-A3TU	16.52703
TCGA-EE-A29P	6.378378
TCGA-D3-A3C7	21.33784
TCGA-EB-A44R	1.054054
TCGA-FR-A3YO	16.16216
TCGA-DA-A3F3	6.702703
TCGA-D9-A6E9	3.337838
TCGA-D3-A51E	6.162162

TCGA-EB-A42Y	1.405405
TCGA-D3-A51H	0.108108
TCGA-D3-A5GU	16.41892
TCGA-D3-A1Q3	0.797297
TCGA-DA-A960	18.02703
TCGA-FS-A1YX	0.608108
TCGA-EB-A6R0	8.162162
TCGA-EE-A3AB	13.13514
TCGA-W3-A828	29.17568
TCGA-D3-A8GI	109.9595
TCGA-EE-A3JD	22.44595
TCGA-D3-A2JA	7.310811
TCGA-EE-A3J7	20.72973
TCGA-FS-A1ZP	9.391892
TCGA-EE-A3J4	6.959459
TCGA-GN-A4U8	8.77027
TCGA-EE-A2GR	33.7973
TCGA-QB-AA9O	31.41892
TCGA-ER-A3ET	2.081081
TCGA-D3-A51N	2.027027
TCGA-EE-A29V	21.41892
TCGA-EE-A29B	12.2027
TCGA-EB-A5UN	14.67568
TCGA-DA-A1I2	2.486486
TCGA-D3-A2JF	31.40541
TCGA-EE-A2ML	11.68919
TCGA-D3-A1Q9	0.797297
TCGA-ER-A199	4.972973
TCGA-FS-A1ZN	2.337838
TCGA-D3-A3MU	10.27027
TCGA-XV-A9VZ	0.283784
TCGA-GN-A267	8.905405
TCGA-D9-A3Z4	3.283784
TCGA-D9-A4Z2	0.405405
TCGA-BF-A1PV	6.135135
TCGA-FS-A1ZR	3.202703
TCGA-3N-A9WD	18.37838
TCGA-BF-A3DL	9.77027
TCGA-FS-A1ZU	0.972973
TCGA-GN-A8LK	43.72973
TCGA-ER-A2NC	8.27027
TCGA-ER-A195	5.283784
TCGA-BF-A1PX	5.459459
TCGA-EB-A550	3.310811
TCGA-ER-A2NF	0.77027
TCGA-EE-A29R	13.60811
TCGA-BF-A5EP	3.472973
TCGA-YD-A9TB	6.513514
TCGA-D3-A3CC	0.662162
TCGA-WE-AAA4	4.081081
TCGA-ER-A2NH	6.675676
TCGA-DA-A3F8	22.97297
TCGA-FR-A7U9	37.27027
TCGA-EB-A24C	0.635135
TCGA-GN-A4U9	1.472973
TCGA-HR-A2OH	2.243243
TCGA-EB-A42Z	2.256757
TCGA-BF-AAOX	26.58108

TCGA-FR-A44A	5.554054
TCGA-WE-A8ZY	4.783784
TCGA-D9-A1X3	0.918919
TCGA-EB-A5UM	11.64865
TCGA-EE-A182	31.31081
TCGA-EB-A5SG	3.567568
TCGA-XV-AAZY	3.405405
TCGA-EE-A29A	5.418919
TCGA-FS-A1ZJ	1.337838
TCGA-EB-A6L9	0.162162
TCGA-EE-A17Z	0.594595
TCGA-GN-A266	80.52703
TCGA-EB-A85J	7.905405
TCGA-BF-A5ES	10.89189
TCGA-GF-A769	3.040541
TCGA-D3-A8GD	1.918919
TCGA-D3-A3MV	10.75676
TCGA-EE-A185	4.256757
TCGA-WE-A8ZN	3.459459
TCGA-DA-A95W	3.581081
TCGA-ER-A42H	1.364865
TCGA-FR-A729	17.86486
TCGA-FW-A3TV	6.959459
TCGA-GN-A265	4.162162
TCGA-EB-A82C	0.689189
TCGA-W3-AA1O	15.59459
TCGA-ER-A19Q	2.756757
TCGA-D3-A1QB	7.189189
TCGA-D3-A5GR	5.054054
TCGA-FR-A2OS	0.351351
TCGA-ER-A19M	9.878378
TCGA-D3-A8GK	12.27027
TCGA-EB-A3XE	0.459459
TCGA-EB-A51B	8.337838
TCGA-WE-A8K5	23.71622
TCGA-W3-AA1V	115.0811
TCGA-3N-A9WC	38.22973
TCGA-XV-AB01	3.432432
TCGA-BF-A5EO	13.36486
TCGA-D3-A3CB	3.918919
TCGA-EE-A2MF	16.64865
TCGA-WE-A8K4	17.2973
TCGA-BF-A1PZ	5.337838
TCGA-D3-A2JK	6.283784
TCGA-ER-A3ES	0.554054
TCGA-ER-A19F	21.35135
TCGA-YG-AA3O	23.67568
TCGA-EB-A3HV	0.878378
TCGA-DA-A1I8	3.256757
TCGA-ER-A19D	9.243243
TCGA-D9-A6EA	13.89189
TCGA-GF-A6C9	45.45946
TCGA-OD-A75X	22.60811
TCGA-EE-A29S	22.12162
TCGA-EB-A57M	0.756757
TCGA-D9-A6EC	88.58108
TCGA-EB-A4OY	1.148649
TCGA-D3-A51F	0.635135

TCGA-YD-A89C	35.87838
TCGA-WE-AAA0	30.98649
TCGA-EE-A3AD	9.540541
TCGA-D3-A8GV	8.662162
TCGA-BF-AAP7	9.256757
TCGA-ER-A198	10.64865
TCGA-DA-A95V	5.391892
TCGA-3N-A9WB	3.648649
TCGA-HR-A2OG	4.081081
TCGA-EB-A431	34.18919
TCGA-DA-A1I4	5.918919
TCGA-FS-A1ZF	4.243243
TCGA-WE-A8ZQ	3.716216
TCGA-D3-A51J	9.243243
TCGA-D3-A51T	24.83784
TCGA-FS-A1ZW	14.45946
TCGA-RP-A695	18.59459
TCGA-EB-A41B	8.702703
TCGA-D3-A2JO	12.82432
TCGA-EE-A2GU	12.75676
TCGA-FS-A1ZQ	10.31081
TCGA-D3-A2J8	8.878378
TCGA-EE-A29E	77.25676
TCGA-W3-AA1R	37.44595
TCGA-EE-A29N	12.87838
TCGA-EB-A44N	2.743243
TCGA-EE-A3JH	2.594595
TCGA-D3-A5GS	8.567568
TCGA-EE-A2A2	32.68919
TCGA-EE-A29G	6.567568
TCGA-D3-A51G	24.97297
TCGA-EE-A2MR	54.64865
TCGA-WE-A8ZM	1.878378
TCGA-EE-A2MQ	5.594595
TCGA-EE-A2M8	2.027027
TCGA-EE-A2GL	5.472973
TCGA-FW-A3I3	1.22973
TCGA-EB-A5FP	8.594595
TCGA-WE-A8JZ	4.432432
TCGA-ER-A19A	0.391892
TCGA-FS-A4FD	9.324324
TCGA-EE-A2GS	7.189189
TCGA-IH-A3EA	33.02703
TCGA-FW-A5DY	1.040541
TCGA-EE-A3J5	46.08108
TCGA-EB-A4XL	9.824324
TCGA-BF-AAP0	6.621622
TCGA-D3-A3ML	19.83784
TCGA-EE-A2MP	6.635135
TCGA-GF-A3OT	11.24324
TCGA-EE-A2MI	22.45946
TCGA-ER-A2NG	5.297297
TCGA-EB-A5KH	0.824324
TCGA-GN-A26A	6.905405
TCGA-D3-A51K	0.554054
TCGA-D3-A8GL	39.47297
TCGA-EE-A20I	0.391892
TCGA-DA-A1HW	7.297297

TCGA-D3-A8GM	82.51351
TCGA-EB-A6QY	12.36486
TCGA-FR-A8YE	3.22973
TCGA-BF-A3DN	1.675676
TCGA-EE-A3AE	30.64865
TCGA-D9-A148	6.040541
TCGA-ER-A2ND	2.432432
TCGA-EE-A3JA	21.40541
TCGA-D3-A3C1	3.067568
TCGA-RP-A693	15.95946
TCGA-W3-A824	31.67568
TCGA-EE-A3AG	35.78378
TCGA-EE-A2MS	60.81081
TCGA-EB-A5UL	9.351351
TCGA-FS-A1Z4	0.716216
TCGA-EE-A2MM	12.2973
TCGA-FS-A4FB	4.094595
TCGA-RP-A6K9	0.783784
TCGA-EE-A20F	10.55405
TCGA-EE-A29Q	6.243243
TCGA-Z2-AA3S	4.513514
TCGA-DA-A1HY	15.67568
TCGA-WE-A8ZR	0.581081
TCGA-BF-A9VF	1.824324
TCGA-FR-A3YN	11.91892
TCGA-BF-A1Q0	14.24324
TCGA-BF-AAOU	6.513514
TCGA-EE-A181	62.71622
TCGA-FS-A1Z0	5.608108
TCGA-ER-A19T	1.013514
TCGA-EB-A85I	4.364865
TCGA-FS-A4FC	15.71622
TCGA-ER-A3PL	6.72973
TCGA-EB-A5SH	2.040541
TCGA-RP-A690	0.364865
TCGA-DA-A1IB	0.054054
TCGA-FS-A1ZB	4.837838
TCGA-W3-AA1W	13.27027
TCGA-WE-AAA3	7.445946
TCGA-EE-A17Y	3.581081
TCGA-ER-A197	0.72973
TCGA-FS-A1ZS	5.297297
TCGA-GN-A4U4	15.43243
TCGA-D3-A1Q6	16.17568
TCGA-FS-A1ZK	32.43243
TCGA-EB-A5SF	0.635135
TCGA-D3-A1Q4	5.283784
TCGA-D3-A5GN	9.662162
TCGA-ER-A19P	4.662162
TCGA-EE-A29D	77.16216
TCGA-EB-A3Y7	23.2027
TCGA-FR-A8YC	63.12162
TCGA-DA-A95Y	9.445946
TCGA-D3-A8GQ	29.87838
TCGA-BF-AAP6	1.405405
TCGA-DA-A1IA	7.391892
TCGA-ER-A1A1	0.445946
TCGA-ER-A19J	4.648649

TCGA-FS-A1YY	4.256757
TCGA-FR-A728	7.540541
TCGA-DA-A1I0	10.83784
TCGA-D9-A6EG	1.702703
TCGA-D3-A8GO	11.31081
TCGA-D3-A2JN	7.094595
TCGA-EE-A2MU	12.86486
TCGA-EB-A1NK	2.864865
TCGA-EB-A3Y6	11.71622
TCGA-D3-A2J7	7.418919
TCGA-D9-A149	5.121622
TCGA-GN-A268	7.486486
TCGA-WE-A8K1	7.162162
TCGA-EB-A6QZ	5.878378
TCGA-EB-A551	6.716216
TCGA-WE-A8ZX	11.97297
TCGA-D3-A3C6	2.297297
TCGA-ER-A19C	1.581081
TCGA-EE-A29W	1.608108
TCGA-BF-A1PU	1.959459
TCGA-ER-A19B	2.202703
TCGA-EE-A2M7	1.162162
TCGA-W3-AA1Q	19.54054
TCGA-GN-A263	9.418919
TCGA-BF-AAP4	15.52703
TCGA-D3-A8GJ	3.783784
TCGA-EE-A29M	50.2973
TCGA-D3-A3CF	0.621622
TCGA-D3-A8GE	0.054054
TCGA-EE-A2A5	7.445946
TCGA-YG-AA3N	41.74324
TCGA-EE-A3JE	3.72973
TCGA-ER-A194	28.41892
TCGA-FR-A3R1	8.648649
TCGA-D3-A3CE	0.891892
TCGA-EB-A97M	6.486486
TCGA-EB-A4OZ	0.162162
TCGA-EE-A20B	3.837838
TCGA-EE-A2ME	0.22973
TCGA-EE-A2MG	4.216216
TCGA-EB-A4P0	3.783784
TCGA-ER-A19O	3.756757
TCGA-EE-A29T	7.756757
TCGA-EE-A29X	2.824324
TCGA-FS-A4F8	2.864865
TCGA-EE-A2GT	7.756757
TCGA-D3-A8GS	4.175676
TCGA-D9-A3Z1	9.459459
TCGA-EE-A2MD	36.78378
TCGA-EE-A3AH	8.824324
TCGA-EE-A2GJ	23.63514
TCGA-EE-A2GC	50.2027
TCGA-EE-A3JB	13.02703
TCGA-D3-A1Q7	1.716216
TCGA-EE-A183	16.12162
TCGA-EB-A430	9.040541
TCGA-Z2-AA3V	8.472973
TCGA-ER-A193	42.54054

TCGA-ER-A19H	5.824324
TCGA-FS-A1Z7	3.675676
TCGA-EE-A2GO	41.43243
TCGA-D3-A8GC	17.90541
TCGA-FS-A1ZM	5.445946
TCGA-HR-A5NC	1.364865
TCGA-EE-A2A0	8.716216
TCGA-EE-A2A1	6.891892
TCGA-ER-A19S	3.216216
TCGA-EE-A2MJ	31.95946
TCGA-EE-A2GN	9.878378
TCGA-FR-A7UA	2.067568
TCGA-D3-A3C8	8.689189
TCGA-EB-A3XC	8.175676
TCGA-LH-A9QB	5.391892
TCGA-D3-A1Q8	4.851351
TCGA-EE-A29H	6.689189
TCGA-D3-A1Q5	4.864865
TCGA-EB-A4IQ	0.189189
TCGA-D9-A4Z5	1.418919
TCGA-DA-A95Z	42.08108
TCGA-FS-A1ZE	3.054054
TCGA-DA-A1IC	21.24324
TCGA-ER-A19L	1.135135
TCGA-XV-A9W5	1.716216
TCGA-FS-A4F9	10.10811
TCGA-FS-A1YW	3.472973
TCGA-D9-A1JX	3.77027
TCGA-GN-A262	7.486486
TCGA-EE-A2GD	11.2027
TCGA-EE-A17X	17.7973
TCGA-D9-A4Z6	5.972973
TCGA-D9-A4Z3	16.95946
TCGA-WE-AA9Y	1.013514
TCGA-EB-A41A	39.60811
TCGA-GF-A2C7	1.756757
TCGA-ER-A2NB	1.054054
TCGA-EB-A553	7.243243
TCGA-D3-A5GL	9.310811
TCGA-FS-A1Z3	22.51351
TCGA-WE-A8K6	5.540541
TCGA-EE-A3JI	26.32432
TCGA-EE-A2A6	6.5
TCGA-GN-A26D	1
TCGA-WE-A8ZT	9.702703
TCGA-FS-A4F4	0.72973
TCGA-EE-A2MK	6.121622
TCGA-D3-A1QA	9.027027
TCGA-EE-A3J8	5.702703
TCGA-XV-AAZV	2.081081
TCGA-EB-A4IS	10.01351
TCGA-FR-A8YD	2.27027
TCGA-ER-A19E	9.932432
TCGA-D3-A51R	15.13514
TCGA-ER-A19W	6.175676
TCGA-BF-A3DJ	2.986486
TCGA-EB-A24D	12.35135
TCGA-DA-A1I1	8.824324

TCGA-D3-A2J6	0.445946
TCGA-GN-A8LL	1.081081
TCGA-YD-A9TA	68.7973
