# Supplementary material for: Identification of tumor mutation burden-related hub genes and the underlying mechanism in melanoma
Source: J Cancer. 2021 Mar 1;12(8):2440–9. doi: 10.7150/jca.53697 (PMC7974884; doi:10.7150/jca.53697)
Supplement: Supplementary file 1 — Supplementary tables. [file jcav12p2440s1.zip › Table-S6-DrugResponse.pdf]

	A101D	A2058	A375	C32	COLO679	COLO792	COLO829
Acetalax	6.748141	4.367728	5.995185	5.256814	6.450382	5.864025	6.348575
Nilotinib	2.78148	3.486873	3.641679	4.379581	1.874636	4.20476	4.376769
SCH772984	0.69328	1.362171	-0.85567	-1.22073	-1.74444	-0.0032	0.132018
Trametinib	-2.56579	-0.79806	-5.20496	-5.18675	-5.65388	-2.68138	-3.30278
Ulixertinib	1.312144	1.764589	0.486499	0.915725	0.079185	0.812744	2.292828
VX-11e	0.979316	2.544676	0.449677	1.496226	0.34158	1.575496	3.004053
AZD5363	1.741831	1.692763	3.602777	3.052322	4.598307	3.436943	3.676368
AZD6738	0.947284	1.142406	1.852143	2.209753	1.632645	4.487373	1.707747
Camptothecin	-2.8641	-3.75743	-4.51742	-3.0154	-2.2607	0.553206	-1.20671
Irinotecan	2.401777	0.895518	0.640628	2.031857	2.831332	4.823112	3.749066
Teniposide	0.231042	-1.1973	-1.67172	-0.30733	1.669093	1.969751	2.632559
Topotecan	0.10339	-0.88427	-2.02882	-0.79759	-0.05486	2.616996	1.310606
AZD2014	0.590959	0.928172	1.242036	2.178177	2.050083	5.048172	3.042668
Daporinad	0	0	-4.89857	-1.26033	-4.02801	0	0
KRAS (G12C) Inhibitor-12	3.391938	4.002662	2.627035	5.22741	4.858645	4.970905	7.862018
WIKI4	3.603683	3.457485	4.073927	4.79209	5.121813	3.034728	4.415374

G361	HT144	IGR1	IGR37	LOXIMVI	MELJUSO	RPMI7951	SH4	SKMEL1
5.514343	5.638207	6.054968	5.271387	4.052807	6.579754	3.3704	5.015104	0
2.817506	2.067063	3.773441	2.827403	3.755415	4.387748	3.133235	2.385086	5.168694
-0.46262	-1.88003	2.11756	-0.80736	-0.96114	1.454761	2.13008	-1.80471	0.753115
-4.17347	-5.41452	-0.4073	-5.35159	-2.52548	-0.5613	-0.25882	-5.7302	-3.33534
1.376268	0.356796	1.93786	0.277155	0.815171	3.073174	2.295696	-0.4822	1.671585
1.07917	-0.26133	2.819302	0.287017	-0.2579	3.400151	2.161534	-0.70949	1.853234
3.818979	2.314148	2.845634	3.023253	1.500829	5.644303	1.734715	4.549999	6.392918
2.232735	1.928152	2.875719	1.483834	2.049236	4.416934	0.697875	4.130809	4.696301
-1.22583	-1.8592	-0.77629	-3.34717	-3.28591	0.292833	-3.92653	-2.06546	-1.36098
2.949863	2.703452	3.266794	2.249649	1.491349	4.817541	1.752883	3.192098	4.063197
1.361855	0.48797	1.92814	-0.38516	-0.4705	2.393496	-1.82751	1.442475	0
1.599063	-0.68644	1.624372	-0.13059	-2.07728	2.096456	-1.63376	1.188161	0
2.42015	1.04768	2.381387	0.318235	0.250608	2.780394	1.158102	1.47501	0
0	0	0	-3.40589	-3.13418	0	0	-3.06321	-3.3396
5.854955	3.95977	5.154903	3.119719	2.988826	6.369626	3.455614	4.965996	0
2.575439	3.412586	3.323396	4.393708	4.679402	5.17971	2.392846	4.246433	6.380172

SKMEL24	SKMEL5	UACC257	UACC62	WM115
7.033156	6.965028	6.516062	6.475672	3.918762
3.983855	0	3.795696	2.067012	3.992258
1.813172	1.079998	-0.30929	-1.4454	1.51448
-0.66578	0	-3.32282	-5.46389	-1.64116
3.35995	0	0.652169	-0.36988	3.074345
2.139178	0	1.720994	0.672338	1.915763
2.717208	0	4.670823	1.84929	1.940649
4.419381	0	6.070318	3.128401	2.980984
-2.96794	-2.56759	0.832252	-3.64115	-2.06218
1.891882	2.564183	4.663011	1.196294	3.627539
0.38412	0.706671	3.288792	0.340891	0.957396
-0.75564	0.384208	3.252761	-1.00565	1.770198
2.707302	1.186843	3.149322	1.854657	1.876349
-0.55402	0	0	-1.48183	-1.64364
6.600555	6.431342	5.201972	5.083737	5.098548
3.449778	0	4.340165	3.484792	4.837081
