# Supplementary material for: Identification of tumor mutation burden-related hub genes and the underlying mechanism in melanoma
Source: J Cancer. 2021 Mar 1;12(8):2440–9. doi: 10.7150/jca.53697 (PMC7974884; doi:10.7150/jca.53697)
Supplement: Supplementary file 1 — Supplementary tables. [file jcav12p2440s1.zip › Table-S5-ceRNA.pdf]

	lnc	miR	mRNA
1	HCP5	hsa-miR-1291	TNNT3
2	AC0794661	hsa-miR-1291	TNNT3
3	H19	hsa-miR-1913	NEXN
4	TEX26AS1	hsa-miR-1913	NEXN
5	TCL6	hsa-miR-1913	NEXN
6	MIAT	hsa-miR-1913	NEXN
7	KCNH1IT1	hsa-miR-1913	NEXN
8	HIPK1AS1	hsa-miR-1913	NEXN
9	MIR99AHG	hsa-miR-1913	NEXN
10	H19	hsa-miR-1913	NEXN
11	TPTEP1	hsa-miR-3127-5p	FLNC
12	BHLHE40AS1	hsa-miR-3127-5p	FLNC
13	FAM66C	hsa-miR-374b-5p	NEXN
14	FAM66B	hsa-miR-374b-5p	NEXN
15	DNAJC19P9	hsa-miR-374b-5p	NEXN
16	MTATP6P1	hsa-miR-374b-5p	NEXN
17	RNF217AS1	hsa-miR-590-3p	NEXN
18	TPTEP1	hsa-miR-590-3p	NEXN
19	NR2F2AS1	hsa-miR-590-3p	NEXN
20	FAM66C	hsa-miR-590-3p	NEXN
21	LINC00861	hsa-miR-590-3p	NEXN
22	LINC00943	hsa-miR-590-3p	NEXN
23	THRBAS1	hsa-miR-590-3p	NEXN
24	MIR99AHG	hsa-miR-590-3p	NEXN
25	ERICH2	hsa-miR-590-3p	NEXN
26	CHRM3AS2	hsa-miR-590-3p	NEXN
27	C1orf143	hsa-miR-590-3p	NEXN
28	LINC01146	hsa-miR-590-3p	NEXN
29	AC0055921	hsa-miR-590-3p	NEXN
30	KCNA3	hsa-miR-590-3p	NEXN
31	MAGI2AS3	hsa-miR-590-3p	NEXN
32	DIAPH2AS1	hsa-miR-590-3p	NEXN
33	EEF1A1P26	hsa-miR-590-3p	NEXN
34	HSPA9P1	hsa-miR-590-3p	NEXN
35	MORF4	hsa-miR-590-3p	NEXN
36	MTATP6P1	hsa-miR-590-3p	NEXN
