# Supplementary material for: Identification of tumor mutation burden-related hub genes and the underlying mechanism in melanoma
Source: J Cancer. 2021 Mar 1;12(8):2440–9. doi: 10.7150/jca.53697 (PMC7974884; doi:10.7150/jca.53697)
Supplement: Supplementary file 1 — Supplementary tables. [file jcav12p2440s1.zip › Table-S4-TIICs.pdf]

	B.cell.naive	B.cell.mem	B.cell.plasr	T.cell.CD8.	T.cell.CD4.	T.cell.CD4.	T.cell.CD4.
TCGA-DA-A95X	0	0.004873	0.005844	0.085071	0	0.045524	0
TCGA-W3-A825	0	0	0.012037	0.228998	0	0	0.062071
TCGA-EE-A29L	0.003278	0.006613	0.00858	0.001382	7.40E-05	0	0
TCGA-BF-A5ER	0	0.000583	0	0.002044	0	0	0
TCGA-D3-A8GP	0	0.004263	1.88E-05	0.00749	0	0	0
TCGA-D3-A2J9	0.036284	0.023671	0	0.573845	0	0	0
TCGA-EB-A299	0	0	0.002141	0.12005	0	0	0
TCGA-GN-A9SD	0.182211	0.001811	0.027591	0.195388	0.007004	0.082305	0.003002
TCGA-GF-A4EO	0	0.047194	0.164114	0.289448	0	0.020019	0.052822
TCGA-EE-A184	0.011037	0	0.043571	0.123907	0	0	0
TCGA-FS-A1ZA	0.000976	0.004231	0.045573	0.067587	0	0.000681	0
TCGA-EE-A2MC	0.028713	0	0.033274	0.366788	0	0	0.095442
TCGA-EE-A2GI	0.020499	0	0.044951	0.222082	0	0	0.025656
TCGA-EE-A2MN	0.009574	0	0.029682	0.199003	0	0	0.020913
TCGA-D3-A8GR	0	0.007538	0.002857	0.003565	0	0	0.00066
TCGA-EB-A82B	0	0	0.058845	0.100379	0	0.010203	0.004266
TCGA-FR-A69P	0.011755	0	0.003701	0.028643	0	0.030623	0
TCGA-DA-A3F5	0	0.001066	7.39E-05	0.025433	0	0	0
TCGA-BF-AAP2	0	0.010568	0	0.058336	0	0.022966	0
TCGA-GF-A6C8	0.007521	0	0.00282	0.1052	0	0.003383	0
TCGA-D3-A8GB	0.03265	0.021175	0.04888	0.191056	0	0.00975	0
TCGA-GN-A26C	0.002112	0.00282	0.027807	0.242473	0	0	0.008044
TCGA-FS-A1ZH	0	0.106816	0.052941	0.26324	0	0.046812	0.076716
TCGA-RP-A694	0.000203	0	0.047727	0.083681	0	0	0.007611
TCGA-D9-A1JW	0.027399	0	0.010525	0.128615	0	0.018547	0.004944
TCGA-FR-A7U8	0.000297	0	0	0.003562	0	0.001136	0
TCGA-FS-A1ZD	0.001422	0	0.00026	0.021819	0	0.049083	0
TCGA-EB-A3XD	0.011887	0	0	0.135892	0	0.012748	0
TCGA-EE-A180	0.002609	0	0	0.008006	0	0.011668	0
TCGA-BF-AAP1	0	0.002565	0	0.007801	0	0.014954	0
TCGA-FS-A1ZZ	0	0.009942	0.002138	0.007164	0	0.008823	0
TCGA-ER-A19G	0.005769	0	0.03321	0.078737	0	0.067455	0.008436
TCGA-DA-A1I5	0	0.014441	0.007366	0.090765	0	0.031379	0
TCGA-D3-A2JP	0	0.046149	0.024021	0.128267	0	0.002791	0.001715
TCGA-EE-A2GM	0.004471	0	0.008968	0.041648	0	0	0
TCGA-D3-A3MO	0	0.014768	0.006196	0	0	0.026286	0.000222
TCGA-D3-A5GO	0	0.002471	5.00E-05	0.045744	0	0	0
TCGA-YG-AA3P	0.000649	0.000448	0.000261	0.010716	0	0	0
TCGA-EE-A3J3	0.003641	0.007448	0.016728	0.034981	0	0.054602	0
TCGA-FS-A1ZC	0	0.001234	0.008931	0	0	0.001417	0
TCGA-EE-A2MH	0.080471	0.082814	6.58E-05	0.153943	0	0.018038	0.007397
TCGA-EE-A2GE	0	0.000145	0.107017	0.342157	0	0	0.03997
TCGA-EE-A2GH	0	0.024358	0.045909	0.275894	0	0	0.018437
TCGA-D3-A2JH	0.066112	0	0.030913	0.508923	0	0	0.059376
TCGA-D3-A2JE	0.015112	0.000429	0.008368	0.005053	0	0.01165	0
TCGA-EE-A2GP	0.001422	0.005677	0	0.034143	0	0.052486	0
TCGA-FR-A726	0.004623	0	0.013189	0.005315	0	0.006472	0
TCGA-D3-A2JB	0.107033	0.284757	0.101877	0.186875	0	0.171646	0
TCGA-EE-A3AF	0	0.032694	0.004345	0.075866	0	0	0
TCGA-XV-A9W2	0	0.000462	0.035138	0.019057	0	0.00451	0
TCGA-D3-A1Q1	0	0.00135	0.001124	0.014979	0	0	0
TCGA-EB-A3XF	0	0.020036	0.000811	0.00465	0	0.039609	0
TCGA-EB-A5VU	0.004325	0	0.004795	0.022861	0	0.014813	0
TCGA-EE-A2GB	0.03023	0	0.042462	0.042584	0	0.002055	0
TCGA-EE-A2MT	0.000482	0	0.0422	0.013366	0	0	0
TCGA-EE-A3AC	0	0.025278	0	0.126627	0	0.050705	0.000769
TCGA-EB-A44O	0	0.008995	0	0.027662	0	0	0

TCGA-FW-A5DX	0.023245	0	0.024384	0.012746	0	0.005059	0
TCGA-ER-A196	0.000575	0.001717	0.006655	0.001215	0	0.009903	0
TCGA-ER-A19K	0	0	0	0.047581	0	0.011586	0
TCGA-DA-A1HV	0	0.026921	0.032671	0.199373	0	0.011231	0.016229
TCGA-D3-A2JD	0.063361	0.071043	0.021888	0.070645	0.024575	0.028665	0
TCGA-EE-A20H	0.003717	0	0.010895	0.020954	0	0	0
TCGA-EE-A29C	0.014652	0	0.014819	0.011681	0	0.010425	0
TCGA-D3-A3MR	0	0.091507	0.012816	0.230066	0	0	0.002611
TCGA-ER-A3EV	0.006191	0	0.033172	0.071746	0	0	0
TCGA-D9-A3Z3	0.080583	0.013819	0.087186	0.086069	0	0	0
TCGA-QB-A6FS	0.012044	0.011262	0.005223	0.10923	0	0	0
TCGA-WE-A8ZO	0	0	0.02908	0.158743	0	0	0
TCGA-Z2-A8RT	0.022368	0.019169	0	0.126962	0	0.043706	0
TCGA-XV-AAZW	0	0.000119	0	0.005887	0	0.008154	0
TCGA-EB-A44Q	0.002473	0.039621	0.018843	0.241267	0	0	0
TCGA-D3-A2JC	0	0.040285	0	0.142886	0	0.034286	0.002734
TCGA-GN-A4U7	0.003496	0.002894	0.003199	0.000606	0	0	0
TCGA-EB-A5SE	0	0.020581	0.010136	0.027691	0	0	0.001857
TCGA-D3-A8GN	0.001091	0	0.143043	0.163158	0	0	0.024242
TCGA-FS-A4F0	0	0	0.010486	0.003068	0	0.002963	0
TCGA-ER-A42K	0	0.007969	0.062752	0.01015	0	0.022341	0
TCGA-EE-A2M5	0.00465	0	0.007754	0.105396	0	0.003073	0.014167
TCGA-ER-A19N	0	0.003919	0.037198	0.180188	0	0.094621	0
TCGA-ER-A2NE	0	0.010164	0.005971	0.006662	0	0.013323	0
TCGA-W3-AA21	0	0.012207	0	0.028678	0	0.001292	0
TCGA-BF-AAP8	0	0	0.007271	0.039075	0	0.007137	0
TCGA-D3-A2JG	0	0.010605	0.019213	0.035736	0	0	0.000602
TCGA-GN-A264	0	0.003543	0	0.017708	0	0.031701	0
TCGA-D3-A3BZ	0.030866	0.002142	0.054819	0.277534	0	0	0.025008
TCGA-EB-A44P	0	0.007615	0	0.103756	0.000323	0	0.000174
TCGA-FS-A1ZY	4.91E-05	0.001022	0.001424	0.010036	0	0	6.55E-06
TCGA-GN-A8LN	0	0.035404	0	0	0	0.059094	0
TCGA-BF-A3DM	0	0.004035	0.018915	0	0	0.008767	0
TCGA-FS-A1ZT	0.011583	0.023714	0.098272	0.246428	0	0.058083	0.023937
TCGA-D3-A3C3	0.024252	0	0	0.084961	0	0.030482	0
TCGA-EE-A2M6	0	0.016866	0.0045	0.017739	0	0	0.000203
TCGA-GN-A4U3	0.005846	0	0.021983	0.032014	0	0	0
TCGA-D3-A5GT	0	0.000931	0.000148	0.002802	0.003487	0	0
TCGA-D3-A2JL	0	0.029337	0.006268	0.097603	0	0	0
TCGA-EB-A3XB	0	0.008251	0.018038	0.054494	0	0	0.0045
TCGA-ER-A42L	0.008688	0	0.069495	0.290983	0	0	0
TCGA-FS-A1ZG	0.00666	0	0.007645	0.007209	0	0.003386	0
TCGA-DA-A1I7	8.23E-05	0	0.004716	0.006586	0	0	0
TCGA-FS-A4F5	0	0.011818	0	0	0.004597	0.007833	0
TCGA-BF-A5EQ	0.023783	0.006961	0.031101	0.110552	0	0.022219	0
TCGA-EE-A3AA	0.089594	0.071161	0	0.108918	0	0.125039	0
TCGA-FW-A3R5	0.040526	0.016141	0.012922	0.04491	0	0.015386	0
TCGA-GN-A4U5	0.010065	0	0.015677	0.245546	0	0	0.046391
TCGA-EE-A20C	0	0.001675	0.001485	0.001732	0	0	0
TCGA-FW-A3TU	0	0.009399	0.006201	0.024946	0	0	0
TCGA-EE-A29P	0	0.03116	0.022936	0.364787	0	0	0.019921
TCGA-D3-A3C7	0	0.00114	0.010584	0.098716	0	0	0.032244
TCGA-EB-A44R	0.003731	0	0.020989	0.154906	0	0	0.001969
TCGA-FR-A3YO	0.000512	0	0	0.001293	0	0	0
TCGA-DA-A3F3	0.002837	0	0.016696	0.031677	0	0.014651	0
TCGA-D9-A6E9	0	0	0.003576	0.413496	0	0	0.039532
TCGA-D3-A51E	0	0.070642	0.045002	0.119334	0	0.050435	0.007394
TCGA-EB-A42Y	0.000132	0	0.004223	0.030013	0	0	0.004183

TCGA-D3-A51H	0.483965	0.413993	0	0.129796	0	0	0.046092
TCGA-D3-A5GU	0.002927	0	0.002576	0.102216	0	0.022985	0
TCGA-D3-A1Q3	0.064579	0.016307	0	0.16458	0	0.135003	0
TCGA-DA-A960	0.00416	0	0.003406	0.00352	0	0	0
TCGA-FS-A1YX	0.002568	0	0	0.021355	0	0.032067	0
TCGA-EB-A6R0	0	0.006266	0.008011	0.18908	0	0	0.020948
TCGA-EE-A3AB	0	0.040184	0.007806	0.042903	0	0	0.003848
TCGA-W3-A828	0.007378	0	0	0.028303	0	0.060711	0
TCGA-D3-A8GI	0.000101	0.00042	0	0.001342	0.001798	0	0
TCGA-EE-A3JD	0.018191	0.398816	0.08695	0.220546	0	0.102299	0.046311
TCGA-D3-A2JA	0	0.044465	0	0.045106	0	0.059813	0.007068
TCGA-EE-A3J7	0.011207	0	0.008757	0.066732	0	0	0.000432
TCGA-FS-A1ZP	0.037539	0	0.017536	0.057272	0	0.015117	0
TCGA-EE-A3J4	0	0.008928	0.001999	0.034678	0	0.009089	0
TCGA-GN-A4U8	0.124753	0.12575	0	0.336421	0	0	0.036556
TCGA-EE-A2GR	0.005407	0	0.009214	0.001408	0	0	0
TCGA-QB-AA9O	0	0.014643	0.005742	0.119124	0	0	0.010286
TCGA-ER-A3ET	0.027098	0.004135	0.016569	0.029008	0	0.061689	0
TCGA-D3-A51N	0	0.065539	0	0.031093	0	0	0
TCGA-EE-A29V	0.000403	0.000259	0.001164	0	0	0.006221	0
TCGA-EE-A29B	1.01E-05	0	0	0.01109	0	0	0.001673
TCGA-EB-A5UN	0	0	0	0.053574	0	0	0
TCGA-DA-A1I2	0	0.030858	0.006437	0.062263	0	0	0
TCGA-D3-A2JF	0.001998	0.034142	0.006906	0.1049	0	0.045111	0
TCGA-EE-A2ML	0.040202	0	0.001569	0.255254	0	0	0.018219
TCGA-D3-A1Q9	0.006021	0	0.02369	0.040947	0	0.062727	0.002619
TCGA-ER-A199	0.107951	0.06123	0.014775	0.238981	0	0.02923	0
TCGA-FS-A1ZN	0	0.000642	0.002847	0	0.009731	0.019777	0
TCGA-D3-A3MU	0.008159	0.007935	0.010456	0.02728	0	0	0
TCGA-XV-A9VZ	0	0.001294	0	0.009321	0	0.045383	0
TCGA-GN-A267	0	0.039458	0.001493	0.075049	0	0	0
TCGA-D9-A3Z4	0.005525	0.003494	0.004453	0.112038	0	0.026277	0
TCGA-D9-A4Z2	0	0.056881	0	0.017047	0.003723	0	0.000249
TCGA-BF-A1PV	0	0.000772	0.003842	0.009588	0	0.007181	0
TCGA-FS-A1ZR	0.004598	0	0.000781	0.059887	0	0.0323	0
TCGA-3N-A9WD	0.022619	0.029427	0	0.080288	0	0	0
TCGA-BF-A3DL	7.82E-05	0	0	0.010544	0	0.003053	0
TCGA-FS-A1ZU	0.000255	0	0.00036	0.000501	0	0.005763	0
TCGA-GN-A8LK	0.004844	0	0.012855	0.008081	0	0	0
TCGA-ER-A2NC	0.001618	0	0.007099	0.030991	0	0.062858	0
TCGA-ER-A195	0.008052	0	0	0.209575	0	0.068342	0.022499
TCGA-BF-A1PX	0	0.009254	0	0.318899	0	0	0.011844
TCGA-EB-A550	0.005092	0	0.001915	0.036274	0	0.036193	0.001298
TCGA-ER-A2NF	0	0.007738	0.051876	0.100735	0	0.005576	0
TCGA-EE-A29R	0	0.002005	0.018932	0.026448	0	0.027396	0
TCGA-BF-A5EP	0.002856	0	0.000487	0.005062	0	0.015892	0
TCGA-YD-A9TB	0	0.039589	0	0.061306	0	0.06075	0
TCGA-D3-A3CC	0.000429	0.001165	0.007939	0	0	0.049438	0
TCGA-WE-AAA4	0	0.021713	0	0.244043	0	0	0.026054
TCGA-ER-A2NH	0	0	0.045321	0.513016	0	0	0.064486
TCGA-DA-A3F8	0.022897	0	0.027746	0.559836	0	0	0.161961
TCGA-FR-A7U9	0	0.0071	0.010127	0.042952	0	0.057807	0
TCGA-EB-A24C	0	0	0.001453	0.008895	0	0.022145	0
TCGA-GN-A4U9	0.002043	0	0.000119	0.016515	0	0.038611	0
TCGA-HR-A2OH	0	0.047344	0.052515	0.538483	0	0	0.072473
TCGA-EB-A42Z	0	0	0	0.03003	0	0.007254	0.000986
TCGA-BF-AAOX	0	0.012981	0	0.013283	0	0.00614	0
TCGA-FR-A44A	0	0	0.011166	0.318927	0	0	0.028064

TCGA-WE-A8ZY	0.01525	0	0.004629	0.02873	0	0	0
TCGA-D9-A1X3	0.000437	0.000503	0.000979	0	0	0.020557	0
TCGA-EB-A5UM	0.020514	0	0	0.102684	0	0.016525	0
TCGA-EE-A182	0.000203	0.000959	0.002422	0.019221	0	0	0
TCGA-EB-A5SG	0.001883	0	0.067502	0.353866	0	0	0.09058
TCGA-XV-AAZY	0	0.008657	0	0.052908	0.008387	0	0
TCGA-EE-A29A	0	0.005633	0.015259	0	0	0.007283	0
TCGA-FS-A1ZJ	0.009083	0	0.003816	0.068698	0	0	0.012887
TCGA-EB-A6L9	0.044015	0.126094	0.002649	0.223328	0.024491	0	0
TCGA-EE-A17Z	0	0.001636	0.004062	0.001077	0	0.015123	0
TCGA-GN-A266	0	0.010608	0.054538	0.10704	0	0.140833	0.005736
TCGA-EB-A85J	0.007875	0	0.000863	0.208903	0	0	0
TCGA-BF-A5ES	0.002544	0.000181	0	0.042547	0	0.020512	0
TCGA-GF-A769	0.001425	0	0.003537	0	0	0.027378	0
TCGA-D3-A8GD	0.144276	0	0	0.284828	0	0.088762	0.050434
TCGA-D3-A3MV	0.033835	0	0.014746	0.029441	0	0	0
TCGA-EE-A185	0	0.00082	0.011631	0	0	0	0
TCGA-WE-A8ZN	0	0.017263	0	0.089879	0	0	0.004982
TCGA-DA-A95W	0	0.003839	0.00797	0.062564	0	0	0
TCGA-ER-A42H	9.87E-05	5.10E-05	0.001287	0.006885	0	0.00987	0
TCGA-FR-A729	0	0.041904	0.013531	0.221201	0	0.079299	0.014616
TCGA-FW-A3TV	0.079867	0	0.021796	0.085066	0	0.026136	0.007001
TCGA-GN-A265	0.001331	0.007954	0.09295	0.106182	0	0.190443	0.014018
TCGA-EB-A82C	0	0	0.010152	0.012098	0	0	0
TCGA-W3-AA1O	0.004348	0	0.006248	0.006268	0.002166	0	0
TCGA-ER-A19Q	0.008426	0	0.013215	0.105529	0	0	0
TCGA-D3-A1QB	0.007665	0	0.029505	0.408609	0	0	0.019164
TCGA-D3-A5GR	0	0.249543	0	0.10056	0	0.145813	0
TCGA-FR-A2OS	0	0.00149	0	0	0	0.024645	0
TCGA-ER-A19M	0	0	0	0.123938	0	0.075596	0.007966
TCGA-D3-A8GK	0.001684	0	0.006614	0.005137	0	0	0
TCGA-EB-A3XE	0	0.027831	0	0.077883	0	0.009002	0
TCGA-EB-A51B	0	0.004089	0.000613	0.044027	0.002201	0	0.000422
TCGA-WE-A8K5	0.001137	1.46E-05	0.004548	0.051401	0	0	0
TCGA-W3-AA1V	0.00255	0	0.002057	0.056203	0	0.077814	0
TCGA-3N-A9WC	0.017517	0	0.031406	0.220262	0	0.136726	0.01452
TCGA-XV-AB01	0	0.013991	0.00633	0.04332	0	0.008468	0
TCGA-BF-A5EO	0	0	0.015101	0.092389	0	0	0
TCGA-D3-A3CB	0.067075	0.01685	0.039959	0.428653	0	0	0.04787
TCGA-EE-A2MF	0.0135	0	0.007172	0.04997	0	0.033812	0
TCGA-WE-A8K4	0	0.064465	0	0.24309	0	0	0
TCGA-BF-A1PZ	0.002407	0	0.006604	0.042384	0	0	0
TCGA-D3-A2JK	0	0.005512	0.000711	0	0	0.006551	0
TCGA-ER-A3ES	0.001116	0	0.003615	0.002983	0	0.0048	0
TCGA-ER-A19F	0	0.00086	0.013909	0.028071	0	0.007665	0
TCGA-YG-AA3O	0.005681	0.004497	0	0.062293	0	0.0024	0.000323
TCGA-EB-A3HV	0.000996	0	0	0.001665	0	0.030037	0
TCGA-DA-A1I8	0	0.025323	0.005255	0	0	0.030186	0.000636
TCGA-ER-A19D	0.01964	0	0.020287	0.085449	0	0.103229	0
TCGA-D9-A6EA	0	0.021995	0	0.00536	0.008834	0	0
TCGA-GF-A6C9	0.037044	0.151929	0.00732	0.081793	0	0	0.014299
TCGA-OD-A75X	0.003556	0	0.002192	0.017791	0	0.039503	0
TCGA-EE-A29S	0	0.006396	0.007466	0.088109	0	0.056604	0
TCGA-EB-A57M	0.006144	0	0.018224	0.023373	0	0.053492	0
TCGA-D9-A6EC	0.000638	0	0.000948	0	0.003564	0	0
TCGA-EB-A4OY	0.001083	0.001401	0	0.02262	0	0	0.000767
TCGA-D3-A51F	0	0.005341	0.019139	0.683652	0	0	0.009509
TCGA-YD-A89C	0	0	0.000346	0.005404	0	0.005647	0

TCGA-WE-AAA0	0.126436	0.008034	0.015823	0.057663	0	0.04431	0
TCGA-EE-A3AD	0.00924	0	0.007155	0.005415	0	0	0
TCGA-D3-A8GV	0	0.000291	0	0.015008	0	0	0
TCGA-BF-AAP7	0	0.041328	0.004487	0.178865	0	0	0.009796
TCGA-ER-A198	0	0.010952	0.006415	0.049192	0	0	0
TCGA-DA-A95V	0.004206	0.002065	0.035942	0.079242	0	0	0
TCGA-3N-A9WB	0	0.000475	0	0	0.008964	0.006191	0
TCGA-HR-A2OG	0	0.009262	0.149418	0.041033	0	0.067792	0
TCGA-EB-A431	0.011703	0	0	0.023956	0	0	0.017331
TCGA-DA-A1I4	0	0.029528	0.003024	0	0	0	0.004307
TCGA-FS-A1ZF	0	0.004179	0	0.019109	0	0	0
TCGA-WE-A8ZQ	0	0.010753	0.018774	0.0065	0	0	0
TCGA-D3-A51J	0	0.092606	0.032652	0.270687	0	0.02511	0
TCGA-D3-A51T	0.059684	0	0.014822	0.061739	0	0.027937	0
TCGA-FS-A1ZW	0	0.004664	0.3632	0.077255	0	0.053396	0.007741
TCGA-RP-A695	0.004293	0	0.017997	0.05508	0	0	0
TCGA-EB-A41B	0.008715	0	0.001415	0.047001	0	0	0
TCGA-D3-A2JO	0	0	0.05795	0.296147	0	0	0.098013
TCGA-EE-A2GU	0.034824	0	0.034307	0.410747	0	0	0.075809
TCGA-FS-A1ZQ	0.010712	0	0.000923	0.027294	0	0.023742	0
TCGA-D3-A2J8	0.040143	0	0.034526	0.331838	0	0	0.058216
TCGA-EE-A29E	0.002027	0	0.02917	0.001152	5.88E-06	0	0
TCGA-W3-AA1R	0	0.006203	0.010686	0.081305	0	0	0.009523
TCGA-EE-A29N	0	0.171643	0	0.159787	0	0.033988	0.048787
TCGA-EB-A44N	0.076973	0.014583	0.074406	0.077698	0	0.082802	0
TCGA-EE-A3JH	0	0.055506	0.077784	0.255938	0	0	0.04096
TCGA-D3-A5GS	0.00229	0	0	0.11471	0	0.012687	0.002387
TCGA-EE-A2A2	0.053879	0.04918	0.044021	0.080148	0	0.073496	0
TCGA-EE-A29G	0.001999	0.004232	0.002333	0.047062	0	0.062764	0
TCGA-D3-A51G	0	0.034444	0	0.044118	0	0	0.001987
TCGA-EE-A2MR	0.039878	0.184143	0.011354	0.302798	0	0.324701	0
TCGA-WE-A8ZM	0.023769	0	0.020126	0.032532	0	0	0
TCGA-EE-A2MQ	0.018491	0	0.009064	0.038781	0	0.038964	0
TCGA-EE-A2M8	0	0.057379	0.029157	0.625999	0	0	0.027713
TCGA-EE-A2GL	0.113673	0.32752	0	0.156281	0.030023	0.194585	0
TCGA-FW-A3I3	0.016776	0	0.044497	0.039932	0	0.000188	0
TCGA-EB-A5FP	0.002762	0	0.000927	0	0	0.026388	0
TCGA-WE-A8JZ	0.009936	0	0.013224	0.032256	0	0.00812	0
TCGA-ER-A19A	0	0.003459	0.004968	0.30979	0	0.260611	0
TCGA-FS-A4FD	0.001951	0	0.002074	0.026933	0	0	0.000526
TCGA-EE-A2GS	0	0.077789	0	0.012526	0	0	0
TCGA-IH-A3EA	0	0.001451	0	0.01394	0	0	0
TCGA-FW-A5DY	0.192817	0.30526	0.022154	0.156549	0	0.13688	0
TCGA-EE-A3J5	0	0.014478	0.006398	0.084187	0	0.086132	0.007945
TCGA-EB-A4XL	0.000149	0.000683	0	0.117627	0	0	0
TCGA-BF-AAP0	0.031653	0.071067	0	0.299938	0	0	0
TCGA-D3-A3ML	0.003699	0	0.003432	0.003936	0	0.001466	0
TCGA-EE-A2MP	0.000634	0	0.028326	0.181942	0	0	0
TCGA-GF-A3OT	0.004858	0	0.002314	0.047864	0	0.019742	0.005558
TCGA-EE-A2MI	0	0.026749	0	0.071007	0	0.057489	0.005337
TCGA-ER-A2NG	0	0	0	0.297288	0	0	0.068432
TCGA-EB-A5KH	0.005444	0.001154	0	0.003829	0	0.022515	0
TCGA-GN-A26A	0.001452	0	0.00916	0.063122	0	0.02577	0.041219
TCGA-D3-A51K	0	0.020273	0.03028	0.048721	0	0	0.0029
TCGA-D3-A8GL	0.003528	0	0.008845	0.000224	0	0	0
TCGA-EE-A20I	0.003226	0	0.015786	0.062478	0	0	0.022517
TCGA-DA-A1HW	0	0.029293	0.061444	0.311729	0	0.11696	0.009249
TCGA-D3-A8GM	0.048451	0	0.017565	0.077024	0	0.027069	0.013594

TCGA-EB-A6QY	0	0	0.038892	0.037219	0	0	0
TCGA-FR-A8YE	0.132496	0.324263	0	0.138991	0.142038	0.327957	0
TCGA-BF-A3DN	0	0.026196	0	0.033705	0	0.023618	0
TCGA-EE-A3AE	0.012773	0	0.04223	0.183893	0	0	0.009857
TCGA-D9-A148	0.001379	0	0.018014	0.060907	0	0	0
TCGA-ER-A2ND	0.005933	0	0.000401	0.036558	0	0	0
TCGA-EE-A3JA	0	0.005593	0.077336	0.084446	0	0	0
TCGA-D3-A3C1	0.020395	0	0.00578	0.06264	0	0	0
TCGA-RP-A693	0.000699	0.01829	0.039679	0.117147	0	0	0.009367
TCGA-W3-A824	0	0.001101	0	0.15203	0	0	0.007139
TCGA-EE-A3AG	0.001319	0	0.00484	0.014505	0	0.012089	0
TCGA-EE-A2MS	0.005398	0	0.066071	0.122331	0	0	0
TCGA-EB-A5UL	0	0.084539	0.059146	0.209103	0	0	0.036766
TCGA-FS-A1Z4	0.150358	0.216086	0.058681	0.208353	0	0.035184	0.005711
TCGA-EE-A2MM	0	0.031801	0.011213	0.035678	0	0.015622	0
TCGA-FS-A4FB	0	0	0.02315	0	0.004824	0	0
TCGA-RP-A6K9	0.001728	0	0.020068	0.096479	0	0.001833	0
TCGA-EE-A20F	0.000256	0	0.048134	0.137226	0	0	0.041112
TCGA-EE-A29Q	0.019342	0	0.003061	0.007957	0	0.015208	0
TCGA-Z2-AA3S	0.003779	0	0.005119	0.063732	0	0	0
TCGA-DA-A1HY	0.000415	0.000741	0.00102	0	0	0.020657	0
TCGA-WE-A8ZR	0.019139	0.038744	0.022527	0.038944	0	0.001172	0
TCGA-BF-A9VF	0.00244	0	0.000875	0.013702	0	0	0
TCGA-FR-A3YN	0.007429	0	0.013597	0.150793	0	0	0
TCGA-BF-A1Q0	0.003788	0.005805	0.003372	0.081075	0	0	0
TCGA-BF-AAOU	0	0.002744	0.125654	0.036133	0	0	0
TCGA-EE-A181	0	0.061058	0.048202	0.188106	0	0	0.010705
TCGA-FS-A1Z0	0.007181	0	0.038188	0.140233	0	0.071146	0
TCGA-ER-A19T	0	0.005082	0	0.022452	0	0.048357	0
TCGA-EB-A85I	0.003443	0	0	0.1307	0	0	0.025667
TCGA-FS-A4FC	0.01942	0	0.00382	0.050901	0	0.022648	0
TCGA-ER-A3PL	0.010657	0	0.008168	0.012625	0	0.014261	0
TCGA-EB-A5SH	0.028162	0.000676	0	0.04161	0	0.024285	0
TCGA-RP-A690	0.01064	0	0.006324	0.005326	0	0.014968	0
TCGA-DA-A1IB	0.163883	0.171531	0.040255	0.289821	0	0.228929	0.016547
TCGA-FS-A1ZB	7.95E-05	0	0	0.024627	0	0.077636	0
TCGA-W3-AA1W	0	0.01345	0	0.559082	0	0	0.0626
TCGA-WE-AAA3	0	0.011996	0.010399	0.103177	0	0.01985	0
TCGA-EE-A17Y	0.000843	0	0.009274	0.002545	0	0	0
TCGA-ER-A197	0	0.00477	0.017756	0.243094	0	0	0
TCGA-FS-A1ZS	0.00868	0	0.003107	0	0	0.025375	0
TCGA-GN-A4U4	0.011363	0.015975	0	0.03412	0	0.011054	0
TCGA-D3-A1Q6	0.006785	0	0	0.031935	0	0.088071	0.006593
TCGA-FS-A1ZK	0.017904	0	0.012377	0	0	0.005558	0
TCGA-EB-A5SF	0.000458	0	0.000254	0	0	0	0
TCGA-D3-A1Q4	0.001616	0.015302	0.024554	0.016953	0	0.009911	0
TCGA-D3-A5GN	0	0.056086	0.038559	0.077926	0	0	0.020268
TCGA-ER-A19P	0.045739	0.124178	0.051613	0.631251	0	0.12973	0
TCGA-EE-A29D	0.000466	0.001199	0.001344	0	0.009846	0	0
TCGA-EB-A3Y7	0.002249	0.003186	0	0.007835	0	0	0.000116
TCGA-FR-A8YC	0	0.002301	0.027454	0.097602	0	0	0.000699
TCGA-DA-A95Y	0.005425	0	1.73E-05	0	0.00253	0	0.000133
TCGA-D3-A8GQ	0.025553	0	0.042707	0.042576	0	0	0.000722
TCGA-BF-AAP6	0.002611	0	8.94E-05	0.004014	0	0	0
TCGA-DA-A1IA	0.002849	0.004675	0	0.00716	0.005368	0	0
TCGA-ER-A1A1	0.442574	0.341417	0.101958	0.203279	0.054444	0.127021	0.061146
TCGA-ER-A19J	0.006897	0	0.01051	0.033384	0	0.005269	0
TCGA-FS-A1YY	0	0.000703	0.000301	0	0	0.008483	0.000298

TCGA-FR-A728	0	0.011192	0	0.16916	0	0.002486	0
TCGA-DA-A1I0	0.004567	0.025823	0.005244	0.033177	0	0.041412	0
TCGA-D9-A6EG	0.000173	0.00061	0.000521	0	0.00604	0.001177	0
TCGA-D3-A8GO	0.076452	0.05096	0.052763	0.064795	0	0.006394	0
TCGA-D3-A2JN	0	0.031933	0.058946	0.135662	0	0	0.043127
TCGA-EE-A2MU	0.010577	0	0	0.195838	0	0	0.02242
TCGA-EB-A1NK	0	0	0.020773	0.039909	0	0.013454	0
TCGA-EB-A3Y6	0	0.033483	0	0.037068	0	0	1.19E-06
TCGA-D3-A2J7	0.00185	0.00356	0.04417	0.151037	0	0.027869	0.004371
TCGA-D9-A149	0.029379	0	0	0.120733	0	0.035775	0
TCGA-GN-A268	0	0.000146	0.000881	0	0.006437	0.022413	0
TCGA-WE-A8K1	0	0.049366	0	0.227639	0	0.012328	0
TCGA-EB-A6QZ	0	0.017538	0.003263	0	0	0	0
TCGA-EB-A551	0.006253	0	0.054114	0.26371	0	0.00083	0.051432
TCGA-WE-A8ZX	0.002975	8.26E-05	0.029033	0.181887	0	0	0.027646
TCGA-D3-A3C6	0.000566	0	0.000502	0	0.001608	0	0
TCGA-ER-A19C	0.000105	0	0.003388	0.003276	0	0.000696	0
TCGA-EE-A29W	0.007812	0	0.004013	0.03272	0	0.018137	0
TCGA-BF-A1PU	0	0.00113	0	0	0	0.011738	0
TCGA-ER-A19B	0	0.005773	0.001518	0	0	0.056197	0.001469
TCGA-EE-A2M7	0.03416	0.120899	0.019479	0.236164	0	0.048338	0
TCGA-W3-AA1Q	0	0.00511	0	0.050972	0	0.033335	0
TCGA-GN-A263	0.004553	0	0	0	0	0.037908	0.001264
TCGA-BF-AAP4	0	0.001526	0.0007	0.175504	0	0	0.069771
TCGA-D3-A8GJ	0	0.015278	0.012224	0.242396	0	0	0.042731
TCGA-EE-A29M	0.020455	0.024103	0.011321	0.177803	0	0	0
TCGA-D3-A3CF	0.08939	0.104011	0	0.130123	0	0.115602	0
TCGA-D3-A8GE	0.003026	0	0.063768	0.202597	0	0.122448	0
TCGA-EE-A2A5	0.000847	0.002169	0	0	0	0.060987	0
TCGA-YG-AA3N	0	0.003934	0.006979	0.244003	0	0	0
TCGA-EE-A3JE	0	0.01826	0.108517	0.439296	0	0	0.092566
TCGA-ER-A194	0	0.008936	0	0.048034	0	0	0
TCGA-FR-A3R1	0.001686	0	0.022329	0.086794	0	0	0.014262
TCGA-D3-A3CE	0	0	0.172067	0.281468	0	0	0.007223
TCGA-EB-A97M	0	0.000595	0.002702	0.052261	0	0	0.005377
TCGA-EB-A4OZ	0	0.030923	0	0.109086	0	0	0
TCGA-EE-A20B	0.001957	0.00253	0.025257	0.141145	0	0.013284	0
TCGA-EE-A2ME	0.107033	0.100117	0	0.607353	0	0.212989	0
TCGA-EE-A2MG	0.153822	0.238482	0.02438	0.202327	0.005531	0.246524	0
TCGA-EB-A4P0	0	0	0.00677	0.049644	0	0	0
TCGA-ER-A19O	0.00622	0.017271	0	0.121328	0	0	0
TCGA-EE-A29T	0	0.013802	0.060341	0.0907	0	0	0.013762
TCGA-EE-A29X	0.003222	0.031952	0.006081	0.088441	0	0.002236	0
TCGA-FS-A4F8	0.008402	0.218195	0	0.302847	0	0.095744	0
TCGA-EE-A2GT	0.163199	0.007837	0	0.116302	0.045232	0.052447	0
TCGA-D3-A8GS	0	0.009264	0.089765	0.20899	0	0	0.042641
TCGA-D9-A3Z1	0	0.045753	0.066468	0.177323	0	0.02742	0.050695
TCGA-EE-A2MD	0.049467	0	0.055878	0.064336	0	0.018896	0
TCGA-EE-A3AH	0.005349	0	0.047022	0.00569	0	0.007749	0
TCGA-EE-A2GJ	0.00362	0	0	0.19806	0	0	0
TCGA-EE-A2GC	0.003593	0	0.102366	0.199854	0	0.0264	0.014757
TCGA-EE-A3JB	0	0.044725	0	0.024386	0	0.037384	0
TCGA-D3-A1Q7	0.02981	0	0.103246	0.402886	0	0.012278	0.064621
TCGA-EE-A183	0.037982	0.079528	0.018614	0.128254	0.02471	0	0
TCGA-EB-A430	0	0.000815	0.012751	0.058743	0	0	0.009855
TCGA-Z2-AA3V	0	0.019442	0	0.061955	0	0	0
TCGA-ER-A193	0.014746	0	0	0.090959	0	0	0.02044
TCGA-ER-A19H	0.011084	0	0.025702	0.133101	0	0.042008	0.001354

TCGA-FS-A1Z7	0	0.0126	0.003035	0	0	0.019858	0
TCGA-EE-A2GO	0	0	0.006331	0.003301	0.000699	0	0.005414
TCGA-D3-A8GC	0.002796	0.001208	0.054151	0.042521	0	0	0.000454
TCGA-FS-A1ZM	0	0	0.020321	0.243818	0	0	0.066677
TCGA-HR-A5NC	0	0.000766	0	0.006538	0	0.011462	0
TCGA-EE-A2A0	0.011933	0.024289	0.123255	0.102867	0	0	0.04001
TCGA-EE-A2A1	0.130793	0	0.021368	0.20688	0	0.158449	0
TCGA-ER-A19S	0	0.057313	0.049114	0.407472	0	0	0.021049
TCGA-EE-A2MJ	0.103493	0.016977	0	0.375631	0	0	0.021274
TCGA-EE-A2GN	0.006931	0	0.014372	0.012471	0	0.004792	0
TCGA-FR-A7UA	0	0	0.043251	0.726066	0	0	0.049492
TCGA-D3-A3C8	0	0	0.001893	0.636221	0	0	0.130322
TCGA-EB-A3XC	0.001233	0	0.007247	0.070567	0	0.000667	0
TCGA-LH-A9QB	0.006297	0	0.000482	0.013549	0	0.013967	0
TCGA-D3-A1Q8	0.006035	0.03158	0.026588	0.050417	0	0.088142	0.002458
TCGA-EE-A29H	0.030877	0.041117	0	0.048833	0	0.029631	0
TCGA-D3-A1Q5	0	0.004499	0.004954	0.019139	0	0.073243	0
TCGA-EB-A4IQ	0.0006	0.000305	0	0	0.004242	0	0
TCGA-D9-A4Z5	0	0.002336	0.000342	0.000924	0	0.027777	0
TCGA-DA-A95Z	0.001198	0	0	0.021944	0	0.006628	0
TCGA-FS-A1ZE	0	0.004998	0	0.016447	0	0.020259	0
TCGA-DA-A1IC	0.006222	0.001458	0.003756	0.05758	0	0.035464	0
TCGA-ER-A19L	0.000625	0	0.01249	0.010575	0	0.023607	0
TCGA-XV-A9W5	0	0	0.011544	0.067111	0	0.008956	0
TCGA-FS-A4F9	0.009092	0	0.005077	0.007338	0	0.00844	0
TCGA-FS-A1YW	0.016313	0.021945	0.011519	0.019298	0	0.037472	0
TCGA-D9-A1JX	0	0.000343	0.042503	0.262568	0	0	0.051819
TCGA-GN-A262	0.000633	0	0.003871	0	0	0.001238	0
TCGA-EE-A2GD	0	0.015192	0	0.025645	0	0.107743	0
TCGA-EE-A17X	0.007141	0	0.001854	0.016096	0	0	0
TCGA-D9-A4Z6	0.00497	0	0.016324	0.018703	0	0	0
TCGA-D9-A4Z3	0	0.004872	0.002284	0.050695	0	0.000432	0.0003
TCGA-WE-AA9Y	0.026079	0.029515	0.033698	0.055971	0	0.097553	0
TCGA-EB-A41A	0.002132	0	0.00868	0.03449	0	0	0
TCGA-GF-A2C7	0.001021	0	0	0.004416	0	0.021329	0
TCGA-ER-A2NB	0	0	0.001164	0.102845	0	0	0
TCGA-EB-A553	0	0.007759	0	0.015093	0	0.000817	0.000998
TCGA-D3-A5GL	0	0.001233	0.009956	0.010668	0	0	0
TCGA-FS-A1Z3	0.004097	0	0	0	0	0.110805	0
TCGA-WE-A8K6	0	0.000514	9.84E-06	0.004152	0	0.004086	0
TCGA-EE-A3JI	0	0.000838	0.002872	0.033573	0	0.016261	0
TCGA-EE-A2A6	0.024033	0.064605	0.023908	0.28446	0	0	0.042238
TCGA-GN-A26D	0.021189	0	0.04015	0.015622	0	0.009659	0
TCGA-WE-A8ZT	0.030631	0	0.026316	0.026004	0.003454	0	0
TCGA-FS-A4F4	0	0.007494	0	0.059111	0	0.002946	0
TCGA-EE-A2MK	0	0.084263	0.015434	0.145663	0	0.054993	0
TCGA-D3-A1QA	0	0.023483	0	0.064097	0	0	0
TCGA-EE-A3J8	0.027688	0	0.004643	0.001992	0	0.002134	0
TCGA-XV-AAZV	0	0.025667	0	0.094629	0	0.001193	0.001752
TCGA-EB-A4IS	0	0.169919	0	0.255458	0	0.081683	0
TCGA-FR-A8YD	0.000989	0.00083	0.000363	0.011008	0	0.001693	0
TCGA-ER-A19E	0	0.007504	0.036615	0.027146	0	0.03564	0
TCGA-D3-A51R	0.032798	0	0.003753	0	0	0.018064	0
TCGA-ER-A19W	0.012337	0	0	0.367163	0	0.064369	0.137552
TCGA-BF-A3DJ	0	0	0.021696	0.042439	0	0.042398	0.004702
TCGA-EB-A24D	0.008311	0	0.078046	0.021312	0	0.051629	0
TCGA-DA-A1I1	0	0.039056	0.008168	0.103825	0	0.120855	0.009129
TCGA-D3-A2J6	0	0	0.004372	0.090437	0	0	0.003826

TCGA-GN-A8LL	0.005721	0	0.017647	0.018435	0	0	0
TCGA-YD-A9TA	0.038968	0	0.012494	0.092296	0	0.047862	0.011599

T.cell.follic	T.cell.regu	T.cell.gamı	NK.cell.res	NK.cell.act	Monocyte	Macropha	Macropha	Macropha
0.032945	0.014605	0	0	0.010409	0.074226	0.012281	0.030817	0.188317
0.104745	0.025469	0	0	0.036611	0.028763	0.18205	0.036182	0.230414
0.009109	0.001372	0	0	0.000196	0	0.011094	0.002667	0.00771
0.00288	0.000105	0	0.001017	0.000225	0.002245	0.023857	0.000596	0.023347
0.018357	0	0	0.007577	0	0	0.032613	0.001928	0.017754
0.213	0.050478	0.198471	0	0.125525	0	0.179524	0.221157	0.259193
0.087152	0.009216	0	0	0.040054	0.018532	0	0.055755	0.128903
0.113258	0.044341	0	0	0.028588	0.021369	0.035765	0.062001	0.15993
0.079957	0.03894	0.061846	0	0.026855	0.004569	0.243216	0.148709	0.210338
0.050825	0.00386	0	0	0.035109	0.015449	0.032183	0.026033	0.072277
0.063045	0.004024	0	0	0.035073	0.013134	0.066219	0.059994	0.114523
0.060955	0.001218	0.003196	0	0.088753	0.0067	0.099779	0.123758	0.239099
0.082599	0.010943	0.012815	0	0.065103	0.0009	0.004491	0.069412	0.098028
0.034463	0.016086	0	0	0.026967	0.025094	0	0.05736	0.150364
0.004534	0.000523	0.001035	0.000568	0	0.003763	0.024956	7.49E-05	0.027377
0.029488	0.013199	0	0	0.028603	0.00738	0.033546	0.053707	0.107659
0.022614	0	0	0	0.011359	0.012098	0.001962	0.021673	0.110172
0.013238	0.006681	0	0.014058	0	0	0.075289	0	0.053825
0.017131	0.035406	0	0	0.026215	0.024616	0.013712	0.046429	0.065141
0.049924	0.02067	0	0.010495	0.023157	0.025943	0.029783	0.070714	0.109114
0.108508	0.063353	0	0	0.070442	0.008233	0.017339	0.088145	0.106398
0.054766	0.004359	0.027931	0	0.019146	0	0.016792	0.081284	0.262606
0.05035	0.020113	0	0	0.074841	0.020614	0.115458	0.130566	0.516561
0.044236	0.004768	0.002343	0	0.024851	0.017498	0.010784	0.018853	0.132237
0.027662	0.049556	0	0	0.059488	0.013097	0.067083	0.067559	0.10034
0.00494	0.00194	0	0.001509	0.001595	0	0.075942	0.003376	0.020725
0.00427	0	0	0.009381	0.040287	0	0.088207	0	0.094236
0.032602	0.06886	0	0	0.032209	0	0.090676	0.054914	0.174124
0.007781	0.00065	0.009765	0	0.001721	0	0.021138	0.022947	0.052594
0.011488	0.001939	0	0.004171	0.005095	0.063967	0	0	0.093646
0.009168	0.002718	0	0.007015	0	0.002592	0.012852	0.008069	0.010534
0.032421	0.007525	0.046027	0	0.044834	0.026012	0.260325	0.136828	0.134986
0.02916	0.033342	0	0	0.013986	0.014044	0.461387	0.062038	0.140831
0.020656	0	0.033846	0	0.028459	0.015911	0	0.076385	0.058003
0.016846	0	0	0	0.013026	0.009261	0	0.010314	0.068054
0.00041	0.004358	0	0.010684	0	0	0.151101	0.002602	0.013228
0.025647	0.0102	0	0	0.006277	0.000122	0.142334	0.019645	0.040259
0.004904	0.002649	0	0	0.000166	0.005573	0.001549	0.006399	0.030273
0.021301	0.009701	0.013149	0	0.002761	0.001247	0.218463	0.054737	0.099527
0.010947	0.001296	0.007348	0.003727	0	0	0.085351	0	0.032052
0.085863	0.069821	0.023805	0	0.049672	0.013392	0	0.050519	0.177861
0.093108	0.01223	0.032769	0	0.045226	0.06752	0.166304	0.078524	0.371533
0.064738	0.021792	0.021939	0	0.093283	0.008111	0.053806	0.07095	0.123219
0.074111	0.006387	0	0	0.125709	0.015978	0	0.055215	0.173182
0.018654	0.004751	0	0	0.008608	0.001348	0.017195	0.006669	0.013192
0	0.003951	0.001692	0	0.023795	0.053707	0.001046	0.03771	0.459802
0.02949	0	0	0.004313	0.005551	0.010992	0.072294	0.003821	0.117552
0.198648	0.004218	0.023893	0	0.072556	0	0.113988	0.101666	0.129207
0.028146	0.004468	0	0	0.01814	0.017035	0.028234	0.042867	0.152176
0.032339	0	0	0	0.019187	0.004806	0	0.047528	0.055158
0.003929	0.0033	0	0.002177	0	0.009875	0.019728	0.000965	0.019212
0.014173	0	0.004099	0	0.034244	0.045144	0.011422	0.067388	0.088228
0.015166	0	0	0	0.008612	0.030698	0.003175	0.012233	0.110338
0.037675	0	0	0	0.00852	0.004284	0.011902	0.038133	0.158415
0.053928	6.54E-05	0.02593	0.004505	0	0	0.043867	0.017879	0.064345
0.05826	0.004677	0	0	0.046416	0.029734	0.023826	0.072891	0.154884
0.036452	0.011702	0	0	0	0	0.027372	0.008756	0.060831

0.015046	0	0.005014	0	0.011214	0	0.019246	0.013346	0.057144
0.01193	0.012918	0	0	0.009443	0	0.158612	0	0.042872
0.047124	0.005166	0.001422	0	0.041595	0.002724	0	0.028777	0.12889
0.03634	0.044522	0	0	0.068012	0.029137	0.036159	0.119841	0.183475
0.009135	0.089315	0	0	0.02256	0.014258	0.038611	0.00273	0.046573
0.027767	0.004374	0.002195	0	0.012131	0.001016	0.061579	0.014582	0.060723
0.009563	0.026414	0.004766	0	0.00605	0	0.163647	0.005784	0.088921
0.046315	0.020737	0.039288	0	0.073096	0	0.158577	0.09358	0.105523
0.033707	0	0	0	0.025581	0.001452	0.025949	0.007421	0.051431
0.077539	0.017235	0	0	0.059399	0.016751	0.054343	0.07614	0.159811
0.028937	0.047324	0	0	0.048884	0	0.044021	0.01708	0.07438
0.026011	0.009828	0	0	0.037512	0.013831	0	0.05108	0.182258
0.061446	0.037528	0	0.005104	0.034491	0.053965	0.027438	0.105756	0.133254
0.025622	0	0	0	0.01025	0	0.037356	0.021537	0.078247
0.059539	0.109065	0.016784	0	0.078027	0.0076	0.017221	0.09306	0.16514
0.045509	0.01917	0.004474	0	0.059027	0.011336	0.114198	0.061681	0.115314
0.003242	0.002955	0	0.002954	0	0	0.038026	0.001335	0.013701
0.016024	0	0.01199	0.004628	0	0.005703	0.016077	0.011667	0.030072
0.057447	0.012166	0.021854	0	0.010879	0.05142	0.058985	0.056609	0.059606
0.00454	0	0	0	0.001468	0	0	0.01006	0.056285
0.010784	0.005152	0	0	0.010345	0.00936	0.011959	0.019701	0.079055
0.016387	0.007833	0.011751	0	0.020249	0.00248	0.043543	0.018927	0.077335
0.022954	0.020496	0	0	0.030124	0.051514	0.083676	0.042966	0.355892
0.010532	0.005018	0	0.009623	0	0.023329	0.023246	0	0
0.006791	0.016408	0	0	0.014144	0.004425	0.008728	0.011633	0.003901
0.012488	0.009472	0	0	0.022314	0.000537	0	0.003554	0.095442
0.007093	0.008994	0	0	0.001453	0.00754	0.016485	0.00722	0.037819
0.004413	0.005374	0	0	0	0.004163	0.027088	0.002098	0.076149
0.083964	0.061346	0	0	0.128502	0.052686	0.094035	0.111853	0.155638
0.029877	0.000757	0	0.008055	0.011339	0.015668	0.003189	0.02222	0.065792
0.0084	0.000681	0	0.007339	0	0	0.015658	0	0.00555
0.028918	0.006675	0	0.016644	0.01288	0.025648	0.031096	0.020647	0.13229
0.009186	0.000562	0	0	0.003597	0.010513	0.025214	0.011338	0.082689
0.08607	0.033316	0	0.001544	0.013325	0.035521	0.026561	0.063722	0.115314
0.051732	0.007132	0.027788	0	0.035619	0	0	0.045275	0.118781
0.012409	0.003647	0	0	0.007127	0.019463	0.196219	0.010223	0.10285
0.011957	0	0	0	0.005131	0.003317	0.002806	0.002507	0.070644
0.004854	0.000318	0	0.002188	0	0.001961	0.00172	0.000394	0.014623
0.033458	0.035169	0.045005	0	0.043017	0	0.101525	0.041269	0.030955
0.043047	0.005002	0	0	0.013713	0.010137	0.00553	0.010866	0.170216
0.134567	0.011703	0.0035	0	0.087863	0.087415	0	0.052625	0.317458
0.007653	0.000528	0	0	0.004846	0.007391	0.002797	0.008484	0.048817
0.023418	0.010839	0.004384	0	0.009858	0	0.14786	0.019396	0.069109
0.008101	8.19E-05	0	0.008217	0.001161	0	0.081212	0.017284	0.042569
0.074225	0.015942	0	0	0.037016	0.01477	0.031376	0.039655	0.18286
0.119944	0.015216	0	0	0.040345	0.023381	0.143967	0.090095	0.144765
0.050661	0	0	0	0.02788	0.000916	0.0359	0.050691	0.164002
0.0931	0.014642	0	0	0.044888	0.019363	0.004014	0.077966	0.068443
0.002971	0.006857	0	0.004387	0	0.003333	0.026018	0	0.017996
0.004226	0.008505	0	0	0.007726	0.007303	0.06828	0.001405	0.016391
0.060756	0.018776	0.036436	0	0.094604	0.04022	0.03142	0.09443	0.144224
0.030548	0.014334	0.00398	0	0.02084	0.018534	0.20532	0.028924	0.13863
0.046476	0.004419	0.033534	0	0.032624	0.001672	0.090958	0.030893	0.101099
0.00359	0.005036	0	0.001773	0.001336	0.010461	0.087332	0	0.018013
0.019661	0.005219	0	0	0.004544	0.00322	0.071245	0.016716	0.075931
0.069671	0.004664	0	0	0.107818	0.016172	0.105808	0.058366	0.106699
0.075693	0	0.011224	0	0.056643	0.008696	0.059196	0.066678	0.091488
0.023473	0	0	0	0.00778	0.009969	0	0.003434	0.116245

0.106062	0.11416	0.038468	0	0	0.003403	0.174384	0.07876	0.121148
0.041135	0.006594	0.008578	0	0.024323	0.00686	0	0.029031	0.149754
0.040711	0.037505	0.011161	0	0.033921	0.011096	0.039678	0.070325	0.129213
0.002704	0	0	0	0.001599	0.000912	0.000909	0.000239	0.010282
0.012961	0.00259	0	0	0.008417	0.026292	0.016822	0.01511	0.203203
0.047874	0	0.006937	0	0.0592	0.007853	0	0.075693	0.074118
0.00734	0.012735	0.00276	0.021921	0	0.064331	0.148339	0.008455	0.193056
0.020437	0.006051	0	0.003808	0.02222	0.036231	0.007937	0.02692	0.27351
0.003021	0.002945	0	0.004213	0	0	0.074903	0.000958	0.01697
0.227627	0.00301	0.185764	0	0.085014	0.010853	0	0.197833	0.182966
0.0238	0.010974	0.000668	0	0	0	0.163882	0.043361	0.398544
0.028178	0	0	0	0.026121	0.004306	0.000622	0.023192	0.071873
0.031007	0.01757	0.005259	0	0.012884	0.006019	0	0.021376	0.253011
0.02258	0	0	0	0.013772	0.00864	0.003916	0.033662	0.06791
0.144594	0.13804	0.066004	0	0.12876	0.017168	0.12036	0.164479	0.217117
0.005524	0	0.001122	0	0.000267	0	0.013263	0.005816	0.017202
0.036263	0.007998	0.017957	0	0.050671	0	0.080552	0.028364	0.042111
0.015589	0.041985	0	0	0.030113	0.058772	0.007205	0.033125	0.392841
0.038371	0.031858	0.011545	0	0.001762	0	0.209158	0.081088	0.067612
0.015935	0.011146	0	0	0	0.007449	0.025996	0.001322	0.023183
0.004984	0.00364	0	0.001949	0.003221	0.017768	0.017175	0.000869	0.080645
0.037295	0	0	0	0.015442	0	0	0.01303	0.038911
0.037991	0.004237	0	0	0.015304	0.018745	0.036149	0.118858	0.04113
0.074497	0.009629	0.017182	0	0.035744	0	0.071683	0.102133	0.102789
0.079016	0.033292	0.016014	0	0.013145	0.006796	0	0.096037	0.171818
0.027999	0.002489	0	0	0.019917	0	0.100358	0.018508	0.097588
0.117905	0.037723	0	0	0.059797	0.042612	0.018129	0.075088	0.166014
0.006472	0.012857	0.0009	0.017204	0	0	0.065776	0.000217	0.028939
0.023177	0.014407	0	0	0.013402	0.005849	0.173972	0.03557	0.056171
0.001704	0.00659	0	0	0.023181	0	0.068023	0.006877	0.025317
0.017526	0.007483	0.001137	0	0.019064	0.002338	0.007409	0.016127	0.036915
0.032986	0.001475	0	0	0.018747	0.021011	0.037843	0.091097	0.181635
0.005827	0.000331	0.010015	0.003312	0	0.010162	0.041063	0.020352	0.09977
0.012421	0.015029	0	0.009382	0	0.007099	0.009788	0.001177	0.033721
0.033439	0.013149	0	0	0.01943	0.032803	0.01081	0.035371	0.127493
0.04709	0.0455	0	0	0.047103	0	0.132714	0.0552	0.024627
0.005372	0.004026	0	0.009457	0.000678	0.003476	0.012764	0.00488	0.027109
0.002112	0.001525	0	0	0.00529	0.00548	0.026064	0	0.027548
0.006521	0	0	0	0.00443	0.001458	0	0	0.007583
0.005671	0.005392	0	0.007363	0.015092	0.056055	0	0.000159	0.41898
0.032792	0.062325	0	0	0.051091	0.049328	0.059413	0.122633	0.403719
0.059126	0.036869	0	0	0.071551	0	0.051415	0.086752	0.156512
0.045394	0.00598	0	0.003541	0	0	0.085937	0.01432	0.091038
0.021079	0.024907	0	0	0.018642	0.016957	0.008948	0.007532	0.021532
0.030352	0	0	0	0.003316	0.005711	0.016823	0.01932	0.069996
0.006502	0.009341	0	0.016556	0	0.005283	0.030446	0	0.013323
0.012554	0	0	0	0.027118	0.034921	0	0.061506	0.155433
0.002291	0.004935	0	0.000563	0.013402	0.014703	0	0	0.154587
0.034335	0	0	0.02718	0.056042	0.063508	0.016449	0.037355	0.09257
0.085151	0.018693	0	0	0.120466	0.07512	0	0.105676	0.167531
0.100462	0	0	0	0.095291	0.05447	0	0.105783	0.138569
0.034853	0.021143	0	0	0.015107	0.021006	0.113125	0.070696	0.26428
0.000718	0.007045	0	0.003832	0.003744	0.010357	0.014104	0.006743	0.03124
0.045131	0.005812	0	0	0.02611	0.034269	0.02986	0.015643	0.116081
0.111182	0.013321	0	0	0.084737	0.099554	0.090036	0.1174	0.104602
0.026908	0.002312	0.000295	0	0.006469	0	0.073247	0.012803	0.078299
0.0142	0.01084	0	0.004208	0	0	0.033431	0.00638	0.009626
0.062154	0	0.1494	0	0.099961	0	0.089247	0.086188	0.201293

0.025665	0	0	0	0.00892	0.012646	0.005298	0.025295	0.164129
0.007833	0.003016	0	0.009279	0	0	0.034702	0.001525	0.024801
0.057739	0.012252	0	0	0.027314	0	0.009056	0.025298	0.047046
0.004107	0.007653	0	0	0.009764	0	0.055048	0.000753	0.048583
0.072212	0	0.027698	0	0.119575	0.010743	0.028653	0.105065	0.143073
0.016256	0.02265	0	0	0.01096	0.005356	0.028213	0.006502	0.038984
0.014595	0.003785	0	0	0.004458	0	0.03638	0	0.0351
0.029911	0	0.000292	0	0.01922	4.56E-05	0	0.015039	0.100393
0.068951	0.064196	0	0	0.011891	0.010797	0.083086	0.051554	0.108714
0.008138	3.56E-05	0	0.005659	0	0.001914	0.009773	0	0.016015
0.041403	0.009999	0	0.001964	0	0.017105	0.201959	0.056521	0.324867
0.103842	0.015527	0	0	0.058741	0.008132	0.214803	0.027743	0.062201
0.024198	0.023888	0	0.004308	0.002153	0.005905	0.018599	0.015763	0.068689
0.017094	0.012532	0	0.005805	0.002878	0.017892	0.045013	0.00029	0.018279
0.162828	0.059674	0.073351	0	0.096553	0.024715	0.200848	0.16666	0.185733
0.004004	0.004015	0	0.004416	0	0.005654	0.079769	0.001239	0.118335
0.004416	0.000713	0.000568	0	0.00042	0	0.0181	0.000187	0.012993
0.019509	0.006002	0	0	0.018775	0.002483	0.032866	0.022975	0.048532
0.019848	0.006378	0.002638	0	0.020804	0.0079	0	0.017046	0.081366
0.003504	0.006374	0	0.009965	0	0	0	0	0.103382
0.111042	0.041116	0.018024	0	0.135387	0.039782	0.057647	0.176778	0.186326
0.036691	0.003449	0	0	0.007524	0.00323	0	0.028161	0.137107
0.057254	0.024204	0.008572	0	0.018679	0.020172	0.092105	0.158656	0.421294
0.004128	0.009019	0	0.009008	0	0.006628	0.022207	0.006406	0.024121
0.002846	0.00179	0	0	0.00253	0	0.081779	0.004943	0.071807
0.060768	0.012118	0.013411	0	0.05765	0.039055	0.211458	0.054171	0.244902
0.105203	0.015739	0.011	0	0.096051	0.008017	0	0.069293	0.094822
0.041666	0.063329	0	0	0.068864	0	0.267709	0.101483	0.111547
0.003333	0.004523	0	0.019341	0	0	0.09534	0	0.065973
0.03235	0	0.05529	0	0.071133	0.015276	0.065574	0.088104	0.105093
0.003641	0	0	0.002736	0	0	0.02315	0.000686	0.018571
0.009691	0.005467	0.002101	0	0.003688	0	0.02496	0.052125	0.103836
0.008892	0.002641	0	0	0.016763	0.002254	0	0.014797	0.040072
0.019835	0.008121	0	0	0.010342	0.005364	0.082369	0.03892	0.081465
0.01087	0.001918	0.020525	0	0.028647	0.023062	0.021952	0.029774	0.264373
0.064322	0.029203	0.012982	0	0.072443	0.006312	0.017656	0.135998	0.061809
0.004168	0.016278	0	0	0.015498	0.003302	0.001076	0.005789	0.034289
0.037109	0.016209	0	0.005428	0.01468	0.039418	0.030091	0.004891	0.084183
0.094941	0.092608	0	0	0.005578	0.028515	0.592117	0.114846	0.400075
0.024002	0.006013	0	0	0.015381	0.017039	0	0.021198	0.192287
0.067299	0.016231	0.012783	0	0.074285	0.004809	0.00306	0.040317	0.021851
0.031967	0.000349	0	0	0.012663	0.00601	0	0.023951	0.055083
0.00237	0.004109	0	0.00384	0	0	0.10187	0.001357	0.026845
0.001029	0	0.000746	0	0.003065	0.000788	0	0.000104	0.01274
0.028412	0.000641	0.002971	0	0.008513	0.005074	0.027443	0.016071	0.101769
0.030576	0.001615	0	0.01225	0.004077	0.014757	0.02882	0.044	0.096865
0.008193	0.006053	8.45E-05	0.002071	0.005191	0.0115	0.019956	0.007265	0.163489
0.001615	0.011434	0	0.015433	0	0	0.169344	0	0.047288
0.045063	0.024467	0	0	0.015612	0.029727	0.165541	0.066545	0.340291
0.013842	9.38E-05	0.012706	0	0	0.004897	0.03583	0.012979	0.049105
0.077788	0.083103	0	0.0028	0.027404	0	0.272035	0.07972	0.273496
0.034006	0	0	0	0.026133	0.003206	0.03095	0.036045	0.113467
0.0328	0.016778	0	0	0.014648	0.015999	0.113973	0.025484	0.110328
0.022513	0.034468	0	0.000947	0	0.017903	0	0.019968	0.181374
0.0037	0.002029	0.00819	0.002717	0	0	0.085505	0.00091	0.049605
0.003186	0.00242	0	0	0.004548	0	0.022482	0.003914	0.027904
0.143293	0.033602	0.135535	0	0.217421	0.004969	0	0.113894	0.198888
0.01786	0.002026	0	0.007242	0	0.014789	0.00328	0.000494	0.039423

0.036703	0.018504	0	0	0.027379	0	0.048671	0.032124	0.106184
0.018343	0	0	0.011309	0	0.003242	0.01983	0.002462	0.041836
0.015564	0.002599	0	0.002124	0.005424	0	0.07287	0.006521	0.034231
0.031913	0.007607	0	0	0.081696	0.02816	0	0.04137	0.130122
0.016506	0.002805	0.036612	0	0.047652	0.029697	0.049334	0.031887	0.21023
0.015442	0.030216	0	0	0.009295	0	0.051745	0.02296	0.069918
0.003055	0.004519	0	0.005114	0.000448	0	0.016861	0	0.023832
0.026043	0.029228	0	0	0.028087	0.009413	0.280271	0.045831	0.236045
0.02893	0.007514	0	0.019131	0	0.004904	0.022844	0.018567	0.081924
0.006486	0.004038	0.017705	0.002146	0	0.009616	0.122675	0.002539	0.159606
0.013645	0.006079	0.007196	0	0	0	0.084336	0.010495	0.043046
0.008387	0.005861	0	0.023697	0	0.007398	0.07249	0.000649	0.034025
0.086902	0.080139	0.049019	0	0.116179	0.055254	0.104195	0.114159	0.105343
0.053277	0.017826	0	0	0.017202	0.024103	0.044811	0.037308	0.122037
0	0	0.019024	0	0.045817	0.005574	0.000656	0.07288	0.123266
0.019342	0.001563	0.003889	0	0.009476	0	0.013311	0.006751	0.069442
0.026273	0	0.003394	0	0.02493	0.005885	0	0.01703	0.025366
0.058312	0	0.068543	0	0.068254	0	0.150153	0.123563	0.194785
0.054811	0.010268	0	0.008933	0.05212	0.044578	0	0.05173	0.177696
0.035121	0.046217	0	0.010644	0.00376	0	0.048037	0.015141	0.1103
0.069626	0.026643	0	0	0.097558	0	0.072238	0.25295	0.269195
0.006555	0	0	0.004428	0	0	0.004779	0.002845	0.019163
0.052057	0.000971	0	0	0.024841	0.003275	0.027061	0.018871	0.036337
0.070425	0.095038	0.019066	0	0.068348	0.023808	0.171023	0.096892	0.1977
0.082157	0.025456	0	0	0.036304	1.20E-05	0.163305	0.059549	0.12066
0.054688	0.07714	0	0	0.085582	0.060954	0.238082	0.137664	0.18668
0.016843	0.013886	0	0	0.039412	0.01478	0.00332	0.046363	0.115927
0.067989	0.017616	0	0	0.023616	0.008894	0.03019	0.024567	0.171402
0.024464	0.015152	0	0	0.04034	0.019041	0.179976	0.039199	0.153667
0.048099	0	0.001869	0	0.012146	0	0.137947	0.044172	0.051029
0.069093	0.061255	0	0	0.039996	0.020903	0.14666	0.110793	0.390849
0.021001	0	0	0.00025	0.003884	0.002405	0.006022	0.001503	0.079504
0.02466	3.87E-05	0	0	0	0.001162	0.040488	0.025653	0.139336
0.118275	0.028564	0.06402	0	0.10721	0.016555	0	0.12534	0.248767
0.117662	0.042156	0	0	0.048808	0.028476	0.015735	0.038968	0.198542
0.052229	0	0	0	0.025496	0.001443	0	0.008732	0.088771
0.014296	0.013407	0	0.005895	0.0016	0	0.033382	0	0.005988
0.015199	0.005231	0	0	0.008212	0	0.11862	0.015047	0.028466
0.110385	0.035155	0	0	0.041674	0.029622	0.299268	0.101397	0.621295
0.012601	0.002372	0.012677	0	0.002608	0	0.080596	0.012966	0.080335
0.024901	0.007398	0.019484	0	0	0	0.15117	0.074418	0.075711
0.008612	0.002239	0	0	0.010297	0.008401	0.018807	0.000997	0.043195
0.050091	0.058337	0	0	0.017076	0.012483	0.056124	0.035051	0.139561
0.05033	0.016944	0	0	0.031771	0.01726	0.050612	0.105062	0.090023
0.072382	0.00854	0	0	0.042638	0.015676	0.009868	0.08347	0.12137
0.079429	0.096775	0	0	0.176951	0	0.034346	0.121496	0.138449
0.003168	0.000134	0	0	0.001897	0	0.000849	0.002716	0.021269
0.041051	0.031	0.007766	0	0.057246	0.026491	0.013291	0.023759	0.168869
0.027569	0	0.009253	0	0.004285	0	0.101407	0.031844	0.125505
0.036122	0.014664	0	0	0.025341	0.043545	0	0.069141	0.194705
0.110868	0	0	0	0.094739	0.030534	0.040446	0.100609	0.122854
0.002656	0.008091	0	0.009461	0	0	0.008052	0.004725	0.017701
0.014549	0	0.025838	0.029091	0	0	0.365239	0.03407	0.134405
0.024479	0.004649	0	0	0.020158	0.002951	0.002291	0.003751	0.047441
0.003787	0	0.000678	0	0.000709	0	0.017471	0.000913	0.032662
0.028846	0.01548	0	0.010038	0.009566	0.00045	0.074053	0.021548	0.078583
0.084655	0.039946	0.018122	0	0.074439	0.009663	0.011804	0.139082	0.117582
0.057324	0.01658	0	0	0.035722	0	0.132267	0.078589	0.149591

0.055924	0	0	0	0.016013	0	0.031624	0.024574	0.053075
0.111217	0.217112	0	0.014708	0.061306	0.043591	0.144432	0.070107	0.334855
0.024678	0.002606	0	0	0	0.008641	0	0.016645	0.080365
0.031334	0.028532	0	0	0.046122	0.044374	0.000526	0.100778	0.126664
0.034915	0.005158	0.000292	0	0.008654	0	0.115275	0.040612	0.031678
0.022117	0	0	0	0.014008	0.004147	0	0.022794	0.078216
0.018553	0	0.05015	0	0.02258	0.008353	0	0.030745	0.185715
0.007834	0.025169	0	0	0.018435	0.001138	0	0.000872	0.103023
0.03334	0.007764	0	0	0.036633	0.02627	0.082709	0.038508	0.335717
0.038592	0	0	0	0.054323	0.019609	0.017572	0.050429	0.0593
0	0.002558	0	0.007396	0	0	0.147531	0.003052	0.015162
0.045435	0.00703	0.016003	0	0.021223	0	0	0.031839	0.187149
0.066539	0.005631	0	0	0.04455	0.012707	0.040582	0.082915	0.02865
0.138075	0.132552	0	0	0.104756	0.052657	0.182279	0.199604	0.244953
0.009127	0.002735	0	4.74E-05	0.001205	0.024452	0	0.010197	0.150831
0.006286	0.001135	0.004229	0.006673	0	0	0.026841	0.003643	0.03277
0.009033	0.00144	0.020867	0	0.024322	0.013579	0	0.010149	0.184255
0.039565	0.007147	0	0	0.040614	0.029871	0.02092	0.06851	0.109408
0.016545	0.002935	0	0	0.009679	0.039673	0.051246	0.002032	0.070563
0.011682	0.005382	0	0	0.023834	0.025028	0	0.004327	0.108142
0.008903	0.00809	0.00159	0.012934	0	0	0.098568	0	0.018137
0.041411	0.019796	0	0	0.015755	0	0.144512	0.020264	0.058891
0.016842	0	0	0	0.005601	6.37E-06	0.004351	0.002694	0.020383
0.04773	0.021808	0.018372	0	0.010839	0	0.015326	0.036529	0.120662
0.048016	0.010804	0	0	0.027974	0.010253	0.011991	0.08558	0.146361
0.045111	0	0	0	0.00876	0.009027	0.018711	0.002594	0.101371
0.056763	0.041491	0	0	0.056235	0.047191	0.194684	0.126208	0.102962
0.080544	0.009517	0	0	0.061298	0.039377	0.121821	0.092845	0.196499
0.000295	0.001172	0	0	0.01393	0.009552	0	0.01495	0.197836
0.050237	0.005005	0	0	0.037923	0.011291	0.008277	0.011094	0.039062
0.047821	0.015015	0	0	0.023277	0.010358	0.066943	0.049516	0.107606
0.021526	0	0.002902	0	0.004872	0.00351	0.096007	0.021438	0.091424
0.020591	0.00919	0	0	0.010908	0.005028	0.013289	0.01016	0.035774
0.003378	0.000512	0	0.001727	0	0.001203	0	0.00141	0.068125
0.112578	0.074585	0.054342	0	0.115301	0	0.358567	0.14174	0.292811
0.007213	0.016573	0	0	0.01689	0.083821	0.074887	0.025586	0.246476
0.15409	0.053006	0.039316	0	0.08047	0.000146	0.046022	0.169018	0.217197
0.011958	0.001498	0	0	0.033228	0.017345	0	0.038545	0.160453
0.013587	0.003862	0	0.003023	0.001532	0	0.109707	0.005671	0.009526
0.084667	0.002086	0.012116	0	0.031216	0.025776	0	0.035602	0.281479
0.015177	0.002461	0	0.004676	0.002219	0	0.087184	0	0.143116
0.021919	0.012663	0	0.011645	0	0.013135	0.011982	0.013104	0.017262
0.023296	0.001531	0	0	0	0.014982	0.019515	0.010816	0.09863
0.002337	0.003211	0	0.002464	0.000203	0	0.04599	0.002674	0.027524
0.001101	0.003942	0	0.004932	0	0	0.041232	0	0.011758
0.019012	0.018318	0	0.005606	0	0.00251	0.129673	0.023048	0.142453
0.059769	0	0	0	0.043954	0.016065	0.165081	0.049054	0.058348
0.138714	0.217693	0.022532	0	0.151723	0.030789	0.103582	0.196657	0.322483
0.00748	0.006456	0	0.015339	0	0	0.05982	0	0.079234
0.005135	0.008221	0	0.002733	0	0	0.044105	0.000667	0.011574
0.019323	0	0.005201	0	0.017655	0.016865	0	0.020133	0.078196
0.005918	0.009013	0	0.009364	0	0.005372	0.017282	0.004895	0.03246
0.019905	0	0.007414	0	0.011583	0	0.130682	0.008423	0.064393
0.034558	0.010488	0	0.000279	0	0.01022	0.096052	0.003343	0.076077
0.019917	0.002959	0	0.003247	0	0.005221	0.002586	0	0.038456
0.111993	0.153672	0	0.01702	0	0	0.163441	0.014923	0.235752
0.007955	0.003687	0	0	0.005603	0	0.072683	0.008194	0.14726
0.003733	0.003033	0	0.011601	0	0	0.047789	0	0.055438

0.065678	0.040293	0	0	0.075152	0.012854	0.01205	0.027167	0.128608
0.015821	0.057889	0	0.006505	0.004668	0	0.049857	0.004306	0.050368
0.007516	0.000997	0	0.003429	7.84E-05	0	0.050083	0	0.037935
0.019255	0.049784	0	0.007314	0	0.001729	0.067561	0.035099	0.052904
0.033395	0.029168	0	0	0.051683	0.0078	0.023064	0.055198	0.047386
0.06078	0.004011	0.00225	0	0.067839	0.00864	0.029835	0.113254	0.290413
0.027533	0.001464	0	0	0.015076	0.003528	0.01455	0.012675	0.099604
0.021303	0.004761	0.011835	0	0.016213	0	0	0.050755	0.084416
0.036923	0.037366	0	0	0.028549	0.014172	0.193023	0.063966	0.098302
0.045329	0.006564	0	0.018706	0.036182	0.029947	0.019588	0.080529	0.175794
0.028492	0.00356	0	0.005624	0.009587	0.006804	0.005155	0.000243	0.043749
0.056232	0.04773	0	0	0.072244	0.020872	0.039211	0.074368	0.07426
0.01573	0.00186	0.006544	0	0	0	0.031009	0.004956	0.062882
0.076056	0.012009	0	0	0.086991	0.005747	0	0.122699	0.183481
0.071175	0	0	0	0.056745	0.032817	0.001182	0.077058	0.065272
0.002234	0.005871	0.007194	0.009142	0	0	0.07595	0.002861	0.064699
0.005666	0.007433	0.000448	0.000601	0	0	0.161138	0	0.03135
0.001411	0.004665	0	0	0.009693	0.008504	0	0.002194	0.152069
0.007868	0.006136	0	0.008804	0	5.37E-05	0.009103	0	0.010855
0.014465	0.017423	0	0.024336	0	0	0.128167	0.000583	0
0.013131	0.068285	0	0	0.033952	0.030231	0.011426	0.011725	0.134578
0.01031	0.009252	0.014072	0	0.017754	0.010355	0.012283	0.042128	0.034574
0.011708	0	0.006944	0.000849	0.002086	0.005256	0	0.02276	0.066319
0.052375	0.002729	0	0	0.072795	0.007315	0.005143	0.028957	0.086146
0.077264	0.003565	0.062713	0	0.060402	0.006175	0.146312	0.053614	0.125273
0.05895	0.043889	0	0	0.074818	0.011318	0.024022	0.103642	0.175571
0.052639	0.081767	0	0	0.111998	0.031854	0.039265	0.086651	0.295566
0.094916	0.024433	0.000673	0	0.000764	0.043675	0	0.061599	0.305007
0.001661	0.018963	0	0.009253	0	0	0.038737	0.0162	0.123437
0.056708	0.022009	0	0	0.051433	0.016604	0.0112	0.013205	0.04107
0.101999	0	0.089788	0	0.074107	0	0.130048	0.087331	0.201334
0.007727	0.013965	0.00023	0	0.012171	0.022358	0.013241	0.008973	0.013569
0.033128	0.003511	0	0	0.036121	0.001714	0	0.009834	0.086347
0.050577	0.010122	0	0	0.086195	0.057192	0	0.079334	0.151035
0.018163	0.007505	0	0	0.015236	0.004142	0	0.007605	0.072016
0.016154	0.026886	0	0	0.047454	0	0.025122	0.038411	0.045933
0.030975	0.010204	0	0	0.019474	0.008027	0.028522	0.050152	0.196642
0.126456	0.169905	0	0	0.120555	0.042094	0.187303	0.211165	0.25907
0.128836	0.02778	0	0	0.032636	0.012942	0.087532	0.113248	0.287955
0.018743	0.003891	0	0	0.003333	0.003523	0	0.012235	0.072087
0.020024	0.049156	0	0	0.035496	0.016302	0.080432	0.017084	0.11762
0.02693	0.003464	0.022631	0	0.015776	0	0.038933	0.017827	0.352576
0.044343	0.013359	0	0	0.041062	0.005994	0.093491	0.032289	0.181252
0.068572	0.117405	0	0	0.122473	0.049796	0.091982	0.114547	0.143695
0.118156	0.033094	0	0	0.019529	0.010095	0.088976	0.013465	0.209958
0.02909	0.014899	0	0	0.041047	0.026998	0.021568	0.069116	0.117933
0.041891	0	0.031728	0	0.043021	0	0.017547	0.03606	0.197093
0.091317	0.002041	0.001891	0	0.014182	2.26E-05	0.082564	0.054585	0.118711
0.010914	0	0	0	0.007189	0.022133	0.013167	0.012564	0.059935
0.047349	0.018817	0.00199	0	0.050278	0.110581	0	0.066506	0.289466
0.059934	0.007739	0	0	0.058724	0.005145	0	0.063882	0.216564
0.016373	0.007155	0.001304	0	0.013401	0.061939	0.120016	0.032705	0.297486
0.098079	0.016997	0.025362	0	0.104512	0.02863	0.194641	0.199599	0.293828
0.03815	0.030643	0	0	0.024532	0.005177	0.107928	0.011544	0.102757
0.03723	0	0	0	0.021431	0.003641	0.007502	0.014977	0.025763
0.02016	0.029813	0	0.004525	0.031935	0	0.138259	0.023077	0.056742
0.061047	0.012736	0.014091	0	0.04349	0	0.257463	0.08861	0.115234
0.035736	0.023079	0	0	0.054806	0.027541	0.018984	0.083365	0.195648

0.007429	0.002838	0.009968	0.013661	0	0	0.275591	0.012617	0.076094
0.006324	0.000302	0.004169	0	0	0	0.076125	0.000435	0.034894
0.029043	0.004351	0	0	0.00441	0.018157	0.079533	0.013183	0.117338
0.061201	0.009216	0	0.009501	0.042914	0.001312	0.096647	0.031462	0.253966
0.006192	0	0	0	0.003563	0.0191	0	0.004028	0.159131
0.01453	0	0.141288	0	0.000203	0.000685	0.007322	0.046109	0.488613
0.076439	0.047047	0	0	0.09533	0.025461	0.035377	0.119317	0.320182
0.081447	0.008399	0.14656	0	0.070701	0	0.172485	0.087299	0.124345
0.101382	0.130229	0	0	0.125935	0.048846	0.0553	0.15634	0.161975
0.009504	0.004115	0	0	0.001918	0	0.111896	0.017716	0.053461
0.13181	0.0381	0.207867	0	0.121064	0	0.191556	0.090377	0.239733
0.078338	0.010507	0.068126	0	0.087592	0.021718	0	0.064379	0.130173
0.043063	0.00136	0	0	0.028574	0.014259	0	0.055763	0.175786
0.014471	0.000426	0.004435	0	0.014686	0.000624	0	0.008813	0.069606
0.011649	0.004227	0	0.004558	0	0.042113	0	0.016753	0.360251
0.031197	0.039349	0	0.016542	0.009432	0	0.115005	0.030631	0.08036
0.010952	0.019468	0.002495	0.00138	0.008276	0	0.141913	0.012913	0.017067
0.016849	0.00351	0	0.022561	0	0	0.099773	0.001578	0.060046
0.004335	0.010754	0	0.012282	0.008155	0	0.032348	0.000159	0.145024
0.013828	0	0.005525	0	0.006898	0	0.016499	0.009008	0.052868
0.011323	0.013683	0	0.003754	0	0	0.010361	0.004848	0.029583
0.01819	0	0	0	0.015873	0.00621	0.010567	0.019469	0.098552
0.005824	0.005179	0	0	0.005813	0.003466	0	0.00685	0.113721
0.068404	0	0	0	0.042256	0.018077	0	0.033391	0.089056
0.010065	0	0	0.002322	0.000126	0.000331	0.005758	0.006032	0.048307
0.021403	0.012905	0	0	0.012158	0.008951	0	0.012564	0.086933
0.047076	0.002695	0	0	0.042907	0.043078	0	0.056011	0.144953
0.002228	0	0	0	0.00052	0	0.003858	0.002085	0.006137
0.024255	0.003658	0	0.023022	0	0.027889	0.016468	0.006363	0.294416
0.00775	0.006474	0	0.012545	0	0.002731	0.053642	0.00281	0.026963
0.017399	0	0	0	0.009717	0.001061	0.091459	0.003602	0.019759
0.01356	0.008444	0	0	0.006543	0.004968	0	0.004405	0.060173
0.031827	0.035485	0	0	0.025295	0.040446	0.021548	0	0.145211
0.022566	0.002638	0	0	0.00703	0.001584	0	0.006064	0.062883
0.007973	0.000921	0	0.011501	0	0.004863	0.036191	0	0.082332
0.024727	0.007186	0	0	0.020873	0.007602	0.00863	0.001504	0.033232
0.003069	0.002405	0	0.000874	0.010522	0	0.029342	0.009518	0.016636
0.013859	0	0	0	0.006423	0.009135	0.018918	0.00316	0.047218
0.013872	0.021774	0	0.00922	0.005578	0.026082	0.000734	0.019486	0.056733
0.006338	0.001604	0	0	0.002941	0	0.093592	0.009963	0.033114
0.02851	0.002145	0	0.001062	0.001682	0.078014	0	0.00707	0.102438
0.096347	0.025019	0	0	0.095753	0.02282	0.018189	0.079596	0.08863
0.023135	0.001971	0	0	0.009762	0	0.039488	0.013418	0.083948
0.014156	0.010293	0	0	0.005285	0.007722	0.000635	0.018019	0.038755
0.014631	0.006104	0.000414	0	0.01762	0.000968	0.080875	0.012632	0.045343
0.031309	0.02832	0.015909	0	0.049395	0.055852	0.010312	0.042141	0.182949
0.034661	0.007634	0	0	0.020925	0.002773	0.054755	0.032228	0.067029
0.005248	0	0.000859	0	0.006994	0	0.006507	0.001351	0.043544
0.045297	0.019244	0	0	0.063559	0.029924	0	0.01759	0.206441
0.105063	0.08137	0	0	0.12326	0.022009	0.065903	0.136092	0.182055
0.006086	0.006105	0	0	0.002081	0	0.071966	0.003113	0.032242
0.007722	0.001252	0	0	0.005955	0.005484	0.07981	0.020871	0.078875
0.006615	0.007358	0	0.000859	0.001381	0	0.01052	0.00082	0.010517
0.08983	0.019862	0.034566	0	0.090188	0.086887	0.027926	0.13725	0.290038
0.019208	0.005167	0	0.004579	0.005547	0.069221	0.048718	0.025703	0.226944
0.030919	0	0.024715	0	0.015589	0.00264	0	0.019181	0.046706
0.049212	0.011917	0	0	0.015214	0.006823	0.161654	0.12293	0.158945
0.028575	0	0.031312	0	0.036338	0	0.025252	0.016081	0.024917

0.00909	0.004528	0	0.0002	0.004332	0	0.024559	0.001141	0.013486
0.061099	0.004788	0	0	0.053219	0.02692	0.255031	0.080273	0.172618

Myeloid.de	Myeloid.de	Mast.cell.a	Mast.cell.re	Eosinophil	Neutrophil
0	0	0.022125	0	0	0
0	0	0	0.012664	0	0
0	0	0	0	0	0
0	0	0.000422	0	0	0
0	0	0.007309	0	0	0
0	0	0	0.001813	0	0
0.0006	0	0.018547	0	0	0
0	0	0	0.003095	0	0
0	0	0	0.012387	0	0
0.001523	0	0.001016	0	0	0
0	0	0.003317	0	0	0
0	0	0	0.067218	0	0.011193
0	0	0	0.003501	0	0
0	0	0	0.000562	0	0
9.68E-05	0	0.023534	0	0.000312	0
0.00264	0	0	0.06737	0	0.000417
0.010815	0	0	0.016341	0	0
0	0.000415	0	0.029892	0	0.048891
0.010279	0	0.02542	0	0	0
0	0	0.00202	0	0	0
0	0	0.009955	0	0	0
0	0	0	0	0	0
0	0	0	0	0	0.005841
0	0	0	0.001372	0	0
0.00241	0	0.052211	0	0	0
0	0	0	0	0	0
0.000797	0	0.013919	0	0	0
0.025581	0	0.041463	0	0	0
0.001349	0	0.034013	0	0	0
0.001569	5.60E-05	0.026427	0	0	0
0	0	0.021055	0	0	0
0	0	0	0.019468	0	0
0	0	0	0.013103	0	0
0.009798	0	0.00499	0	0	0
0.001534	0	0	0.000154	0	0
0	0	0.003837	0	0	0
0	0	0	0.012912	0	0
0.000166	0	0.008474	0	0.000856	0
0	0	0.016677	0	0	0
0	0	0	0.001179	0	0
0.006297	0	0.016282	0	0	0
0	0	0	0.010439	0	0
0.014357	0	0.029721	0	0	0
0.011723	0	0	0.021655	0	0.005946
0	0	0	0.000991	0	0
0.006072	0	0.056673	0	0	0
0	0	0	0.018184	0	0.008388
0	0	0.017768	0	0	0
0.006585	0	0.005301	0	0	0
0.002319	0	0.021524	0	0	0.004807
0	0	0.009647	0	0	0
0.003729	0	0.033309	0	0	0
0.004628	0	0.04837	0	0	0
0	0	0	0	0	0
0	0.00051	0	0.00471	0	0
0.008381	0	0.02827	0	0	0
0.000933	0.001428	0.028867	0	0	0

0	0	0.001561	0	0	0
0	0.024015	0	0.056549	0	0.011888
0.022293	0.00269	0.005395	0	0	0.015676
0.010556	0	0.002297	0	0	0
0	0	0.003475	0	0	0
0	0	0.00248	0	0	0
0	0	0.017162	0	0	0
0	0	0	0	0	0
0.002247	0	0	0.009011	0	0
0	0	0.05821	0	0	0
0.003434	0	0.004197	0	0	0.001747
0	0	0	0.000906	0	0.000339
0.011752	0	0.037085	0	0	0
0	0	0	0.001182	0	0.002593
0	0	0.001854	0	0	0
0.001954	0	0	0	0.009309	0
0	0	0	0.000701	0	0
0.002206	0	0.008865	0	0	0
0	0	0	0.065694	0	0
0	0	0.001849	0	0	0
0.000718	0	0.007399	0	0	0
0	0	0	0.062932	0	0.027205
0	0	0	0.006208	0	0
0.000216	0	0.012122	0	0.003303	0
0.001638	0	0.010446	0	0	0
0	0	0	0.001175	0	0
0.000811	0	0.022156	0	0.000344	0
0	0	0.019687	0	1.11E-05	0
0.017406	0	0.050696	0	0	0
0.001258	0	0	0.034445	0	0
0.000415	0	0.019086	0	0	0
0	0	0	0	0	0
0.001029	0	0.0394	0	0.004153	0
0.002541	0	0	0.001155	0	0
0.004799	0	0	0.000469	0	0
0.033232	0	0	0.022364	0	0
0	0	0	0.001406	0	0
0.000827	0	0.007018	0	6.73E-05	0.00017
0	0	0.011401	0	0	0
0.002162	0	0	0.015876	0	0.000551
0.016807	0	0	0	0	0
0.001413	0	0.001361	0	0	0
0	0	0	0.001383	0	0
0	0	0.00402	0	0	0
0	0	0	0.0061	0	0
0	0	0.014669	0	0	0
0	0	0	0.008647	0	0
0.009855	0	0	0.013672	0	0
0	0	0.004303	0	0.000103	0
0	0	0	0.005686	0	0
0.008011	0	0	0.022061	0	0
0.005204	0	0	0.047067	0	0
0	0	0	0.012921	0	0
0	0	0	0.017994	0	0
0	0	0	0.002478	0	0
0	0	0	0.016077	0	0
0	0	0	0.004788	0	0
0	0	0	0.002714	0	7.05E-05

0	0	0.000936	0	0	0
0.000144	0	0	0.002252	0	0
0.000315	0	0.043278	0	0	0
0	0	0.000547	0	0	0
0.000158	0	0	0.009342	0	0
0	0	0	0.00576	0	0.00018
0.000771	0	0	0.05389	0	0
5.75E-05	0	0.058731	0	0	0.000148
0	0	0.001902	0	0.000726	0
0	0	0	0	0	0.003831
0	0	0	0.007612	0	0
0	0	0	0	0	0
0.004748	0	0.004578	0	0	0
0.002753	0	0.005148	0	0	0
0.007626	0	0.018213	0	0	0
0	0	0.002198	0	0	0
0.00316	0	0	0.007937	0	0
0	0	0.003543	0	0	0.000583
0	0	0	0.035354	0	0
0	0.000629	0.025734	0	0.000524	0
0.000503	0	0.003008	0	0	0
0.022333	0	0	0.087837	0	0.063225
0.009219	0	0.028075	0	0.004429	0
0	0	0.01833	0	0	0
0.005975	0	0	0.010338	0	0
0	0	0.006954	0	0	0
0.018353	0	0.001811	0	0	0
0	0.000909	0	0.042323	0	0
0	0	0.002048	0	0	0
0.0075	0	0.045207	0	0	0
0	0	0.001237	0	0	0
0.012893	0	0.043152	0	0	0
0.011981	0	0.028945	0	0	0
0.001136	0	0.02145	0	0	0
0.001481	0	0.016981	0	0	0
0.002363	0	0.008818	0	0	0
0	0	0	0.001609	0	0
0.001215	0	0	0.009045	0.000674	0
0	0.000191	0	0.000867	0	0
0	0.002625	0.027554	0	0	0
0.006206	0	0.016376	0	0	0
0.014194	0	0.009493	0	0	0
0.0012	0.007242	0	0.015727	0	0.005391
0	0.000122	0.00011	0	0	0
0	0	0.004896	0.007663	0	0
0.000167	0	0	0.005104	0.000736	0
0.01028	0	0.022004	0	0	0
0.000807	0.001429	0.055131	0	0	0
0.010085	0	0	0.030891	0	0
0.015864	0	0	0.024394	0	0
0.019556	0	0	0.035851	0	0
0	0	0.01886	0	0	0
0	0	0.056731	0	0	0
0	0	0.001057	0	0	0
0.014013	0	0	0.023767	0	0
0	0	0.015422	0.002958	0	0
0	0	0	0.001477	0	0
0	0	0	0.020792	0	0

0	0	0.002549	0	0	0
0	0	0	0.003799	0	0
0.000992	0	0.004941	0	0	0
0	0	0.005342	0	0	0
0.014726	0	0	0.028124	0	0
0	0	0.002225	0	0	0
0	0	0.004515	0	0.000785	0
0	0	0	0.001772	0	0
0	0	0.007206	0	0	0
3.62E-05	0	0.018836	0	0.00078	0
0	0	0.006885	0	0	0
0	0	0	0.0149	0	0
0.000966	0	0.005557	0	0	0.001659
0	0	0.004898	0	0	0.000187
0	0	0	0	0	0
0	0	0	0	0	0
0	0	0	0.001404	0	0.000524
0.004152	0	0.008638	0	0	0
0	0	0	0	0	0
0.000206	0	0.002042	0	0.001742	0
0.020102	0	0.065462	0	0	0
0.000588	0	0	0	0	6.11E-05
0	0	0.015984	0	0	0
0	0.003363	0.004317	0	0	0
0	0	0.002583	0	0	0
0.012924	0	0.032041	0	0	0
0.009375	0	0	0.023681	0	0
0	0	0.016055	0	0	0
0	0	0	0.031621	0	0
0.009195	0	0.003058	0	0	0
0	0	0	0.002361	0	0
0.015202	0	0.015841	0	0	0
0.002876	0	0.028999	0	0	0
0	0	0.010019	0	0	0
0	0	0	0.018592	0	0
0.009593	0	0	0.014189	0	0.00188
0.000191	0	0	0.000347	0	0.000879
0	0	0	0.024466	0	0
0.004679	0	0.011052	0	0	0
0	0	0.010388	0	0	0
0.005151	0	0	0.014073	0	0
0	0	0	0.002999	0	0.000301
0	0	0.005338	0	0	0
0	1.55E-06	0	0.001273	0	0
0	0	0	0.005346	0	0
0.006221	0	0.010109	0	0	0
0.007854	0	0.030534	0	0	0.003252
0	0	0.001711	0	0	0
0	0	0.011026	0	0	0
0.001986	0	0.025964	0	0	0
0	0	0.034937	0	0	0
0	0	0	0.009409	0	0
0.00057	0	0.008362	0	0	0
0.009038	0	0.03937	0	0	0.006894
0	0	0.01281	0	0	0
0	0	0.003491	0	0	0
0.023997	0	0	0.046707	0	0.00221
0	0	0	0.007636	0	0

0	0	0	0	0	0
0	0	0	0.00546	0	0
0	0	0	0	0	0
0.010458	0	0.015385	0	0	0
0.005867	0	0.009537	0	0	0
0	0	0.005317	0	0	0
0	0	0.00432	0	0	0
0	0	0.026297	0	0	0.005389
0	0.010565	0	0.008998	0	0.008504
0.003997	0	0.012002	0	0	0
0	0	0.011931	0	0.002403	0
0.003614	0	0.005987	0	0	0
0.008419	0	0.014984	0	0	0
0	0	0.001126	0	0	0
0.006137	0	0	0	0	0
0	0	0	0.000126	0	0
0.00014	0	0	0.001723	0	0
0.015295	0	0	0	0	0
0.004978	0	0	0	0	0
0	0	0.024331	0	0	0
0	0	0	0.017833	0	0
0	0	0	0.002139	0	0.009587
0.002846	0	0.000756	0	0	0
0	0	0.023339	0	0	0
0.008141	0	0.036917	0	0	0.000998
0.031734	0	0.028022	0	0	0
0.003701	0	0	0.014298	0	0
0	0	0.003217	0	0	0
0.038864	0	0.061622	0	0	0
0	0	0.008445	0	0	0
0	0	0.022525	0	0	0
0	0	0.004349	0	0	0
0.000481	0	0	0	0	0
0.017093	0	0	0.024237	0	0
0.015017	0	0.017122	0	0	0
0	0	0	0.016552	0	0
0.000256	0	0.00186	0	0.001149	0
0	0	0.003387	0	0	0
0	0	0.016479	0	0	0
0	0	5.25E-06	0	0	0
0.00061	0	0.003707	0	0	0
0.002859	0	0	0.024721	0	0
0	0	0	0	0	0
0.011656	0	0.004597	0	0	0
0.011313	0	0.045642	0	0	0.001401
0.013785	0	0.059554	0	0	0.004828
0	0	0.000776	0	0	0
0	0	0	0	0	0
0	0	0	0.038736	0	0.000975
0.008458	0	0.021033	0	0.00166	0
0.016701	0	0	0	0	0.001524
0	0	0.010156	0	0	0
0	0	0	0.038283	0	0
0.00026	0	0.010924	0	0	0
0	0	0.001874	0	0	0
0	0	0	0.002282	0	0
0.015124	0	0.002631	0	0	0
0.013914	0	0.005177	0	0	0

0.010384	0	0	0.008727	0	0
0.009204	0	0.013988	0	0	0
0.003299	0	0.017952	0	0	0
0	0	0	0	0	0
0	0	0	0.000965	0	0
0	0	0	0.004901	0	0.004331
0	0	0	0.005584	0	0
0	0	0	0.011881	0	0.001148
0	0	0	0.016018	0	0
0.006761	0	0.016705	0	0	0
0	0	0	0.012334	0	0
0	0	0	0.003783	0	0
0.008216	0	0	0.00137	0	0
0	0	0.042252	0	0	0
0.000907	0.001261	0.017176	0	0	0
0	0	0.001483	0	0	0
0.000433	0	0	0.002033	0	0
0.020757	0	0	0.006891	0	0
0	0	0.01277	0	0	0
0	0	0.016927	0	0	0
0	0	0	0.022653	0	0
0	0	0	0	0	0
0	0	0	0.00562	0	0
0	0	0	0	0	0
0.006219	0	0.020906	0	0	0
0	0.005585	0	0.004968	0	0.004578
0	0	0	0.026759	0	0
0.005865	0	0	0.004166	0	0
0.000701	0	0.015112	0	0	0
0	0	0	0.022837	0	0
0.001763	0	0.003186	0.001482	0	0
0	0	0	0.011297	0	0
0	0	0.000207	0	0	0
0	0	0	0	0	0
0.011649	0	0.056127	0	0	0
0.009672	0	0.027718	0	0	0
0.015277	0	0	0	0	0
0.003892	0	0	0.01149	0	0
0	0	0	0.013756	0	0
0	0	0	0.026993	0	0.004535
0	0	0.000909	0	0.000369	0
0.001324	0	0.013365	0	0	0
0	0.015864	0	0.025367	0	0.022039
0	0	0	0	0	0
0	0	0	0.001492	0	0
0	0	0.02669	0	0	0
0	0	0.013568	0	0	0
0.006142	0	0	0	0	0
0	0	0.008548	0	0	0
0	0.000812	0	0.002876	0	0
0.001358	0	0	0.01719	0.007242	0
0.001132	0	0.024499	0	0	0
0	0	0	0.01115	0	0
0	0	0	0.016919	0	0
0	0.003183	0	0	0	0
0	0.035094	0	0	0	0
0	0	0	0.000927	0	0
0	0	0.009879	0	0.000292	0

0	0.001276	0	0.026387	0	0.010069
0.000366	0	0.008552	0	0	0.001192
0	0	0.002271	0	0	0
0.000564	0	0	0.010611	0	0.001972
0.029407	0	0.004014	0	0	0
0	0	5.09E-05	0	0	0
0.005567	0	0.004403	0.007666	0	0.002461
0.008167	0	0.024042	0	0	0
0	0	0.009618	0	0	0
0.003358	0	0.01866	0	0	0
0.002023	0	0.020128	0	0.003573	0
0.005914	0	0	0.007602	0	0
0.000808	0	0.008066	0	0	0
0.011181	0	0	0.02783	0	0
0.004794	0	0	0.003978	0	0
0	0	0.005485	0	0	0
0	0	0.011512	0	0	0
0	0	0.019859	0	0	0
0	0	0.006953	0	0.000753	0
0.000557	0	0.016708	0	0	0
0	0	0.003161	0	0	0
0.002167	0	0.01861	0	0	0
0.00036	0	0.013246	0	0	0
0.001987	0	0	0.002953	0	0
0	0	0	0.01225	0	0
0.011638	0	0.013985	0	0	0
0.001823	0	0.071122	0	0	0
0.0019	0	0	0	0	0
0.00189	0	0.017597	0	0	0
0.006293	0	0	0.010225	0.001088	0
0.012048	0	0	0.060938	0.001232	0
0.001432	0	0.002535	0	0	0
0.000401	0	0	0.006226	0	0.000878
0.004319	0	0	0.009138	0	0
0.000134	0	0	0.012467	0	0
0.007836	0	0.003957	0	0	0.004274
0.002339	0	0.010722	0	0	0
0.005898	0	0.02283	0	0	0
0	0	0.034572	0	0	0
0.000529	0	0.000691	0	0	0
0.002746	0	0.011255	0	0	0.001752
0	0	0	0	0	0.000238
0.00828	0	0	0.005263	0	0
0	0	0.079152	0	0	0
0	0	0.010059	0	0	0
0.001974	0	0	0.001485	0	0
0.002883	0	0	0.003755	0	0
0	0	0.000321	0	0	0
0.001703	0	0.003561	0	0	0
0	0	0	5.89E-06	0	0
0	0	0	0	0	0.000517
0.002435	0	0	0.032635	0	0
0	0	0.01494	0	0	0
0	0	0.010381	0	0	0
0	0	0	0.007259	0	0
0	0	0.015828	0	0	0
0	0	0.033855	0	0	0
0.005438	0	0.039322	0	0	0

0	0	0.003146	0	0	0
0	0	0.000275	0.000489	0	0
0.000318	0	0	0.00132	0	0
0	0	0	0	0	0
0.009672	0.001741	0.042039	0	0	6.65E-05
0	0	0.003557	0	0	0.006444
0	0	0.039941	0	0	0
0	0	0	0.04388	0	0
0.003305	0	0.064901	0	0	0
0	0	0.000665	1.92E-05	0	0
0.007621	0	0	0.061266	0	0
0	0	0	0.011723	0	0
0.002891	0	0	0.023425	0	0.001516
0.003286	0	0	0.010678	0	0
0.000515	0	0.073452	0	0	0.000558
0	0	0.015385	0	0	0
0	0	0.036959	0	0	0.000709
0	0.00092	0	0.006241	0.002946	0
0	0	0.001454	0	0	0.001062
0	0	0.002665	0	0	0
0.001114	0	0.019335	0	0	0
0.008701	0	0.044545	0	0	0
0	0	0.006318	0	0	0.00031
0	0	0	0.013837	0	0
0	0	0.000997	0	0	0
0	0	0.008902	0	0	0
0.004665	0	0	0.008686	0	0
0	0	0	0	0	0
0	0.014135	0	0.069405	0	0.003592
0	0	0.001384	0	0	0
0	0	0	0.010772	0	0
0	0	0.000949	0	0	0
0	0	0.011163	0	0	0
0	0.000261	0.001476	0	0	0
0.000104	0.001988	0	0.004609	0	0
0.001846	0.000316	0	0.018661	0	0
0.000408	0	0.010339	0	0	0
0	0	0.000793	0	0	0
0.001339	0	0.006145	0	0	0
0	0	0.000138	0	0	0
0	0	0.026039	0	0	0
0.001205	0	0	0.029883	0	0
0	0	0	0.015081	0	0
0	0	0.004146	0	0	0
0	0	0.001863	0	0	0
0.001865	0	0	0	0	0
0.005629	0	0.008306	0	0	0
0	0	0	0.002339	0	0
0.001044	0	0.009348	0.00514	0	0.000567
0.004007	0	0.042763	0	0	0
0	0	0.005716	0	0	0
0	0	0	0.000389	0	0
0	0	0	0.002867	0	0
0.009033	0	0.040664	0	0	0
0	0	0.005731	0.017228	0	0.004331
0.000652	0	0.004471	0	0	0
0.005578	0	0.021114	0	0	0.001953
0	0	0	0.009718	0	0

0	0	0	0.00016	0	0
0	0	0	0.021465	0	0
